# Efficacy and safety of combining commercial Chinese polyherbal preparation with conventional medicine in the treatment of coronary microvascular disease: a systematic review and network meta-analysis

**DOI:** 10.3389/fphar.2026.1846574

**Published:** 2026-06-04

**Authors:** Xiaoying Guo, Qianrong Li, Hugang Jiang, Chunzhen Ren, Chang Shu, Xinke Zhao, Yingdong Li

**Affiliations:** 1 Teaching Experiment and Training Center, Gansu University of Chinese Medicine, Lanzhou, China; 2 School of Traditional Chinese and Western Medicine, Gansu University of Chinese Medicine, Lanzhou, China; 3 Gansu Province Key Laboratory of Chinese Medicine for the Prevention and Treatment of Chronic Diseases, Lanzhou, China; 4 Key Clinical Specialty of the National Health Commission of the People’s Republic of China, Key Specialized Cardiovascular Laboratory, National Administration of Traditional Chinese Medicine, Lanzhou, China; 5 Affiliated Hospital of Gansu University of Chinese Medicine, Lanzhou, China

**Keywords:** commercial Chinese polyherbal preparation, conventional medicine therapy, coronary microvascular disease, network meta-analysis, randomized controlled trial

## Abstract

**Objective:**

To systematically assess and compare the efficacy and safety of 19 commercial Chinese polyherbal preparation for coronary microvascular disease (CMVD) through a frequentist network meta-analysis (NMA) of randomized controlled trials.

**Methods:**

A comprehensive search was performed across multiple databases through November 2025 to identify studies evaluating the effectiveness of CCPPs in treating CMVD. Two independent reviewers screened studies, extracted data, and assessed risk of bias. The NMA was conducted using a frequentist random-effects model, and statistical analyses were performed in Stata 18.0. Network diagrams, league tables, and SUCRA (surface under the cumulative ranking curve) plots were generated to compare and rank the treatments.

**Results:**

Sixty-seven randomized controlled trials (RCTs) involving 6,139 patients were included. All CCPPs combined with conventional medicine (CM) demonstrated potential advantages in efficacy compared to CM alone, but the optimal agent is highly outcome-specific. Yixinshu Tablets may be more favorable for improving microvascular function by reducing the index of microcirculatory resistance (IMR) and increasing coronary flow reserve (CFR). Tongxinluo Capsule showed a relatively greater reduction in corrected thrombolysis in myocardial infarction frame count (cTFC) and a higher total effective rate in the included studies. Shexiang Tongxin Dripping Pills was associated with a more pronounced elevation in left ventricular ejection fraction (LVEF). Danshen Dripping Pills ranked highest for reducing endothelin-1 (ET-1) and increasing nitric oxide (NO) levels. Yindan Xinnao Tong Capsules exhibited a relatively greater decrease in high-sensitivity C-reactive protein (hs-CRP). Additionally, Shexiang Baoxin Pills was associated with the lowest risk of adverse events.

**Conclusion:**

The combination of CCPPs with CM for CMVD may be associated with improved clinical outcomes compared to CM alone. However, these initial results require confirmation through high-quality, rigorously designed clinical trials.

**Systematic Review Registration:**

https://www.crd.york.ac.uk/PROSPERO/view/CRD420251245224, identifier CRD420251245224.

## 1 Introduction

Coronary microvascular disease (CMVD) is defined as a clinical syndrome involving structural or functional abnormalities of the coronary arterioles (typically less than 500 μm in diameter), resulting in impaired coronary flow reserve and myocardial ischemia (Chen et al., 2023). Pathophysiologically, CMVD encompasses a range of alterations, including endothelial dysfunction, vascular smooth muscle cell abnormalities, microvascular rarefaction, perivascular fibrosis, and increased microvascular resistance, all of which contribute to inadequate myocardial perfusion (Gurgoglione et al., 2025). CMVD can be classified into four primary types: those associated with myocardial ischemia, myocardial infarction, coronary revascularization, and non-atherosclerotic heart disease. This multifactorial condition is prevalent in various cardiovascular diseases, including both obstructive and non-obstructive coronary artery disease, heart failure with preserved ejection fraction, and cardiomyopathies such as diabetic, dilated, or hypertrophic forms. In these settings, CMVD significantly influences disease progression and patient outcomes (Rigattieri et al., 2023).

CMVD is highly prevalent. A meta-analysis of 56 studies found that among 14,427 patients with non-obstructive coronary artery disease who met inclusion criteria, 41% had CMVD (Mileva et al., 2022). Additionally, 20%–30% of patients undergoing coronary angiography for stable angina show microvascular dysfunction despite having no coronary blockages (Godo et al., 2021). The condition is particularly common in postmenopausal women: a study of over 2,000 patients with low-to-moderate risk chest pain reported that 82% of those diagnosed with CMVD were female, with an average age of 51 years (Safdar et al., 2020; Thakker et al., 2022). CMVD is strongly linked to a higher risk of major adverse cardiovascular events (MACE). Its risk factors largely overlap with traditional cardiovascular risks, including older age, hypertension, smoking, faster heart rate, and lower high-density lipoprotein cholesterol (HDL-C) levels (Thakker et al., 2022). Importantly, it significantly raises the likelihood of cardiovascular events (such as myocardial ischemia, angina, and heart attacks) and all-cause death in people with ischemic heart disease, making it a key factor affecting their clinical outcomes (Crea et al., 2014; Bajaj et al., 2018; Lin et al., 2025).

The etiology of CMVD remains incompletely understood, and the condition is frequently underdiagnosed in clinical settings. The absence of standardized, targeted therapies presents substantial obstacles to the systematic prevention and management of coronary heart disease, with significant implications for patient outcomes. Consequently, early diagnosis and intervention are essential. Current management strategies are largely empirical, focusing on lifestyle modification, optimization of cardiovascular risk factors, and anti-ischemic pharmacotherapy (Chen et al., 2023). Nevertheless, conventional agents such as nitrates, beta-blockers, calcium channel blockers, and renin-angiotensin system inhibitors often demonstrate limited efficacy in certain patient populations, underscoring persistent therapeutic challenges (Bairey Merz et al., 2020; Pompei et al., 2024).

In traditional Chinese medicine (TCM), the symptoms of CMVD align with the syndrome “Xiong Bi Xin Tong” (chest discomfort and heart pain). commercial Chinese polyherbal preparation (CCPP), as standardized TCM formulations, are recognized for their potential in the prevention and management of CMVD. These agents exert therapeutic effects through multi-metabolites, multi-target, and multi-pathway mechanisms, characterized by broad pharmacological actions, minimal adverse effects, and favorable patient adherence. Accumulating clinical and experimental evidence indicates that CCPPs benefit CMVD via several molecular pathways: (1) protection of coronary microvascular endothelial cells through upregulation of endothelial nitric oxide synthase (eNOS) and increased nitric oxide (NO) production; (2) enhancement of vascular endothelial function by modulating the balance between endothelin-1 (ET-1) and NO; (3) suppression of inflammatory responses by reducing high-sensitivity C-reactive protein (hs-CRP), interleukin-6 (IL-6), and tumor necrosis factor-α (TNF-α); (4) mitigation of oxidative stress by increasing superoxide dismutase (SOD) activity and decreasing malondialdehyde (MDA) levels; (5) promotion of angiogenesis via the vascular endothelial growth factor (VEGF) pathway; and (6) improvement of hemorheological parameters (Yang et al., 2022; Cui et al., 2023; Yang et al., 2023; Ge et al., 2026). The multi-faceted pharmacological profiles of CCPPs are well aligned with the complex pathophysiology of CMVD, potentially offering advantages over single-target conventional biomedicine therapies.

Conventional pairwise meta-analyses have primarily addressed direct comparisons between individual commercial Chinese polyherbal preparation and conventional therapy, limiting the ability to compare different proprietary formulations directly. Network meta-analysis (NMA) overcomes this limitation by integrating both direct and indirect evidence within a unified analytical framework, thereby enabling simultaneous comparison and ranking of multiple interventions through a common comparator (Brignardello-Petersen and Guyatt, 2025). The use of surface under the cumulative ranking curve (SUCRA) probabilities further facilitates the identification of interventions with the highest likelihood of optimal efficacy for specific clinical outcomes. Accordingly, this study employed a rigorous frequentist NMA with a random-effects model, in strict accordance with PRISMA-NMA 2020 reporting standards, to systematically evaluate and rank the comparative efficacy and safety of major CCPPs combined with conventional therapy in patients with CMVD (Huang et al., 2025). The primary objective is to provide clinicians with a comprehensive, evidence-based hierarchy to inform precision-guided selection of adjunctive therapies in the integrated management of CMVD.

## 2 Methods

This NMA was conducted by the PRISMA Extension guidelines for systematic reviews, specifically those related to network meta-analyses of healthcare interventions (Hutton et al., 2015; Page et al., 2021). The detailed PRISMA checklist is provided in Supplementary Appendix S1. The review protocol was prospectively registered in advance with the International Prospective Register of Systematic Reviews (PROSPERO: CRD420251245224).

### 2.1 PICOS criteria

- This study was designed and implemented based on our prospectively registered PROSPERO protocol with a standard PICOS framework. The evaluation of transitivity and consistency for network meta-analysis was pre-specified in the original protocol, and no material unplanned methodological changes were made throughout the research process. Participants were patients diagnosed with CMVD according to the criteria outlined in the Chinese Expert Consensus on the Diagnosis and Treatment of Coronary Microvascular Diseases (Chen et al., 2023), including all CMVD types, regardless of sex, cause, ethnicity, or disease severity.
- Intervention involved the use of CCPPs in addition to conventional medications. All included CCPPs are commercially available proprietary medicines officially approved by the National Medical Products Administration (NMPA) of China, excluding hospital-prepared formulations. Each preparation has a valid national drug registration number as presented in Table 1, and is manufactured referring to unified official quality specifications. In addition, we have finished relevant assessment of these preparations in accordance with the ConPhyMP reporting framework (Heinrich et al., 2022). In terms of raw material sources, the animal-derived ingredients contained in these preparations have relatively standardized application specifications in clinical practice. The formulations adopt officially approved artificial substitutes for some rare animal medicinal materials, and part of animal-derived raw materials are derived from standardized artificial breeding sources, which reduces the dependence on wild protected animal resources to a certain extent. Examples include Shexiang Baoxin Pills (SBP), Shexiang Tongxin Dripping Pills (STDP), Tongxinluo Capsule (TC), Qishen Dropping Pills (QDP), Xinkeshu Tablets (XT), Xinbao Pills (XP), Danshen Dripping Pills (DDP), Yindan Xinnao Tong Capsules (YXTC), Kuanxiong Aerosol (KA), Xueshuantong Capsules (XC), Yangxin Shengmai Granules (YSG), Shenxiang Suhe Pills (SSP), Lingbao Huxin Dan (LHD), Qili Qiangxin Capsules (QQC), Liqi Huoxue Dripping Pills (LDP), Dengzhan Shengmai Capsules (DSC), Yuxintong Capsules (YC), Yixinshu Tablets (YT), and Guanxinning Tablets (GT).
- Control was treated with conventional medicine (CM), such as antiplatelet drugs, beta-blockers, calcium channel blockers, ACE inhibitors/ARBs, statins, or nicorandil.
- Outcome measures were categorized into primary and secondary outcomes. The primary outcome measures were microcirculatory function indicators, including the index of microcirculatory resistance (IMR), coronary flow reserve (CFR), and corrected Thrombolysis in Myocardial Infarction frame count (cTFC). The secondary outcome measures included the total effective rate, left ventricular ejection fraction (LVEF), high-sensitivity C-reactive protein (hs-CRP) (mg/L), Endothelin-1 (ET-1) (ng/L), nitric oxide (NO) (µmol/L), and adverse events.
- Study type was a randomized controlled trials (RCTs) published domestically and internationally, regardless of allocation concealment or blinding status. All studies had complete patient information, and there were no language restrictions.

**TABLE 1:** Characteristics of the included CCPPs.

CCPPs	Manufacturer	Constituent(s)	Usage and dosage (medicine instruction)	Quality control reported?	Production batch number	Chemical analysis reported? (Y/NR)
Shexiang Baoxin pill	Shanghai Hutchison Pharmaceuticals Co., Ltd.	*Artificial Moschus (substitute for Moschus berezovskii Flerov [Moschidae; Moschus* * *])*, *Panax ginseng* C.A.Mey. [Araliaceae; Ginseng radix et rhizoma], *Artificial Bovis Calculus (substitute for Bos taurus Linnaeus [Bovidae; Bovis calculus* * *])*, *Cinnamomum verum* J.Presl [Lauraceae; Cinnamomi cortex], *Liquidambar orientalis* Mill. [Altingiaceae; Styrax], *Bufo gargarizans* Cantor [Bufonidae; Bufonis venenum*], *Cinnamomum camphora* (L.) J.Presl [Lauraceae; Borneolum syntheticum]	1-2 pills (22.5 mg/pill), tid, po	Y-prepared according to NMPA: Z31020068	-	NR
Shexiang Tongxin Dripping pills	Inner Mongolia Conba Pharmaceutical Co., Ltd. Shenglong Branch	*Artificial Moschus (substitute for Moschus berezovskii Flerov [Moschidae; Moschus* * *])*, *Panax ginseng* C.A.Mey. [Araliaceae; Ginseng radix et rhizoma], *Bufo gargarizans* Cantor [Bufonidae; Bufonis venenum*], *Salvia miltiorrhiza* Bunge [Lamiaceae; Salviae miltiorrhizae radix et rhizoma], *Bos taurus* Linnaeus [Bovidae; Bovis calculus], *Ursus thibetanus* Cuvier [Ursidae; Fel ursi*(farm-raised)], *Cinnamomum camphora* (L.) J.Presl [Lauraceae; Borneolum syntheticum]	2 pills (35 mg/pill), tid, po	Y-prepared according to NMPA: Z20080018	-	NR
Tongxinluo Capsules	Shijiazhuang Yiling Pharmaceutical Co., Ltd.	*Panax ginseng* C.A.Mey. [Araliaceae; Ginseng radix et rhizoma], *Buthus martensii* Karsch [Buthidae; Scorpio*], *Hirudo nipponica* Whitman [Hirudinidae; Hirudo*], *Eupolyphaga sinensis* Walker [Corydiidae; Eupolyphaga seu Steleophaga*], *Scolopendra subspinipes mutilans* L. Koch [Scolopendridae; Scolopendra*], *Cryptotympana pustulata* Fabricius [Cicadidae; Cicadae periostracum*], *Paeonia lactiflora* Pall. [Paeoniaceae; Paeoniae radix alba], *Cinnamomum camphora* (L.) J.Presl [Lauraceae; Borneolum syntheticum], *Santalum album* L. [Santalaceae; Santali albi lignum], *Dalbergia odorifera* T.C. Chen [Fabaceae; Dalbergiae odoriferae lignum], *Boswellia carterii* Birdw. [Burseraceae; Olibanum], *Ziziphus jujuba var. spinosa* (Bunge) Hu ex H.F. Chow [Rhamnaceae; Ziziphi spinosae semen]	2-4 capsules (0.26 g/capsule), tid, po	Y-prepared according to NMPA: Z19980015	1410010	NR
Xinkeshu tablets	Shandong Wohua Pharmaceutical Science and Technology Co., Ltd.	*Salvia miltiorrhiza* Bunge [Lamiaceae; Salviae miltiorrhizae radix et rhizoma], *Pueraria lobata* (Willd.) Ohwi [Fabaceae; Puerariae lobatae radix], *Panax notoginseng* (Burkill) F.H.Chen [Araliaceae; Notoginseng radix et rhizoma], *Crataegus pinnatifida* Bunge [Rosaceae; Crataegi fructus], *Aucklandia lappa* Decne. [Asteraceae; Aucklandiae radix]	4 tablets (0.31 g/capsule), tid, po	Y-prepared according to NMPA: Z37020042	-	NR
Xinbao pills	Guangdong Xinbao Pharmaceutical Science and Technology Co., Ltd.	*Datura metel* L. [Solanaceae; Daturae flos], *Panax ginseng* C.A.Mey. [Araliaceae; Ginseng radix et rhizoma], *Cinnamomum verum* J.Presl [Lauraceae; Cinnamomi cortex], *Aconitum carmichaelii* Debx. [Ranunculaceae; Aconiti lateralis radix praeparata], *Cervus nippon* Temminck [Cervidae; Cervi cornu pantotrichum* (farm-raised)], *Cinnamomum camphora* (L.) J.Presl [Lauraceae; Borneolum syntheticum], *Artificial Moschus (substitute for Moschus berezovskii Flerov [Moschidae; Moschus* * *])*, *Panax notoginseng* (Burkill) F.H.Chen [Araliaceae; Notoginseng radix et rhizoma], *Bufo gargarizans* Cantor [Bufonidae; Bufonis venenum*]	2-6 pills (60 mg/pill), tid, po	Y-prepared according to NMPA: Z44021843	-	NR
Danshen Dripping pills	Tasly Pharmaceutical Group Co., Ltd.	*Salvia miltiorrhiza* Bunge [Lamiaceae; Salviae miltiorrhizae radix et rhizoma], *Panax notoginseng* (Burkill) F.H.Chen [Araliaceae; Notoginseng radix et rhizoma], *Cinnamomum camphora* (L.) J.Presl [Lauraceae; Borneolum syntheticum]	10 pills, (27 mg/pill), tid, po	Y-prepared according to NMPA: Z10950111	161,201	NR
Yindan Xinnao Tong Capsules	Guizhou Bailing Enterprise Group Pharmaceutical Co., Ltd.	*Ginkgo biloba* L. [Ginkgoaceae; Ginkgo folium], *Salvia miltiorrhiza* Bunge [Lamiaceae; Salviae miltiorrhizae radix et rhizoma], *Erigeron breviscapus* (Vaniot) Hand.-Mazz. [Asteraceae; Erigerontis herba], *Gynostemma pentaphyllum* (Thunb.) Makino [Cucurbitaceae; Gynostemmatis herba], *Crataegus pinnatifida* Bunge [Rosaceae; Crataegi fructus], *Allium sativum* L. [Liliaceae; Allii sativi bulbus], *Panax notoginseng* (Burkill) F.H.Chen [Araliaceae; Notoginseng radix et rhizoma], *Blumea balsamifera* (L.) DC. [Asteraceae; Blumeae folium]	2-4 capsules (0.4 g/capsule), tid, po	Y-prepared according to NMPA: Z20027144	20130330/20140610/20160120	NR
Qishen Dripping pills	Tasly Pharmaceutical Group Co., Ltd.	*Salvia miltiorrhiza* Bunge [Lamiaceae; Salviae miltiorrhizae radix et rhizoma], *Panax notoginseng* (Burkill) F.H.Chen [Araliaceae; Notoginseng radix et rhizoma], *Astragalus membranaceus* (Fisch.) Bunge [Fabaceae; Astragali radix], *Dalbergia odorifera* T.C. Chen [Fabaceae; Dalbergiae odoriferae oleum]	1 pill, (0.5 g/pill), tid, po	Y-prepared according to NMPA: Z20030139	171,202	NR
Kuanxiong Aerosol	Zhejiang Sukoan Pharmaceutical Co., Ltd.	*Cinnamomum camphora* (L.) J.Presl [Lauraceae; Borneolum syntheticum], *Asarum sieboldii* Miq. [Aristolochiaceae; Asari radix et rhizoma], *Santalum album* L. [Santalaceae; Santali albi lignum], *Alpinia officinarum* Hance [Zingiberaceae; Alpiniae officinarum rhizoma], *Piper longum* L. [Piperaceae; Piperis longi fructus]	2–3 sprays, (5.8 g/60 actuations) prn, sublingual	Y-prepared according to NMPA: Z20163023	-	NR
Xueshuantong Capsules	Guangdong Zhongsheng Pharmaceutical Co., Ltd.	*Astragalus membranaceus* Fisch. ex Bunge [Fabaceae; Astragali radix], *Panax notoginseng* (Burkill) F.H.Chen [Araliaceae; Notoginseng radix et rhizoma], *Scrophularia ningpoensis* Hemsl. [Scrophulariaceae; Scrophulariae radix], *Salvia miltiorrhiza* Bunge [Lamiaceae; Salviae miltiorrhizae radix et rhizoma]	3 capsules, (0.5 g/capsule), tid, po	Y-prepared according to NMPA: Z20030017	220,230/230,419	NR
Yangxin Shengmai Granules	Qinhuangdao Shanguan Pharmaceutical Co., Ltd.	*Panax ginseng* C.A.Mey. [Araliaceae; Ginseng radix et rhizoma], *Ophiopogon japonicus* (Thunb.) Ker Gawl. [Asparagaceae; Ophiopogonis radix], *Salvia miltiorrhiza* Bunge [Lamiaceae; Salviae miltiorrhizae radix et rhizoma], *Schisandra chinensis* (Turcz.) Baill. [Schisandraceae; Schisandrae chinensis fructus], *Dimocarpus longan* Lour. [Sapindaceae; Longan arillus], *Lycium barbarum* L. [Solanaceae; Lycii fructus], *Paeonia veitchii* Lynch [Paeoniaceae; Paeoniae radix rubra], *Achyranthes bidentata* Blume [Amaranthaceae; Achyranthis bidentatae radix], *Curcuma aromatica* Salisb. [Zingiberaceae; Curcumae radix], *Aucklandia lappa* Decne. [Asteraceae; Aucklandiae radix], *Citrus medica* L. [Rutaceae; Citri medicae fructus], *Poria cocos* (Schwein.) F.A. Wolf [Polyporaceae; Poria], *Alisma plantago-aquatica* L. [Alismataceae; Alismatis rhizoma], *Glycyrrhiza uralensis* Fisch. ex DC. [Fabaceae; Glycyrrhizae radix et rhizoma]	1 bag, (14 g/bag) tid, po	Y-prepared according to NMPA: Z20030096	-	NR
Shenxiang Suhe pills	Hangzhou Hu Qing Yu Tang Pharmaceutical Co., Ltd.	*Artificial Moschus (substitute for Moschus berezovskii Flerov [Moschidae; Moschus* * *])*, *Cinnamomum camphora* (L.) J.Presl [Lauraceae; Borneolum syntheticum], *Bubalus bubalis* (Linnaeus, 1758) [Bovidae; Bubali cornus*], *Boswellia carterii* Birdw. [Burseraceae; Olibanum], *Styrax tonkinensis* (Pierre) Craib ex Hartwich [Styracaceae; Benzoinum], *Atractylodes macrocephala* Koidz. [Asteraceae; Atractylodis macrocephalae rhizoma], *Cyperus rotundus* L. [Cyperaceae; Cyperi rhizoma], Aucklandia lappa Decne. [Asteraceae; Aucklandiae radix], *Aquilaria sinensis* (Lour.) Spreng. [Thymelaeaceae; Aquilariae lignum resinatum], *Syzygium aromaticum* (L.) Merr. and L.M.Perry [Myrtaceae; Caryophylli flos], *Liquidambar orientalis* Mill. [Altingiaceae; Styrax]	1bottle, (0.7 g/bottle), bid, po	Y-prepared according to NMPA: Z33020141	22,063	NR
Lingbao Huxin Dan	Leiyunshang Pharmaceutical Group Co., Ltd.	*Salvia miltiorrhiza* Bunge [Lamiaceae; Salviae miltiorrhizae radix et rhizoma], *Panax ginseng* C.A.Mey. [Araliaceae; Ginseng radix et rhizoma rubra], *Bufo gargarizans* Cantor [Bufonidae; Bufonis venenum*], *Artificial Bovis Calculus (substitute for Bos taurus Linnaeus [Bovidae; Bovis calculus* * *]), Artificial Moschus (substitute for Moschus berezovskii Flerov [Moschidae; Moschus* * *])*, *Cinnamomum camphora* (L.) J.Presl [Lauraceae; Borneolum syntheticum], *Panax notoginseng* (Burkill) F.H.Chen [Araliaceae; Notoginseng radix et rhizoma], *Liquidambar orientalis* Mill. [Altingiaceae; Styrax]	3-5 pills, (0.08 g/pill), tid, po	Y-prepared according to NMPA: Z32021181	05,002	NR
Qili Qiangxin Capsules	Shijiazhuang Yiling Pharmaceutical Co., Ltd.	*Astragalus membranaceus* Fisch. ex Bunge [Fabaceae; Astragali radix], *Panax ginseng* C.A.Mey. [Araliaceae; Ginseng radix et rhizoma], *Aconitum carmichaelii* Debeaux [Ranunculaceae; Aconiti lateralis radix praeparata], *Salvia miltiorrhiza* Bunge [Lamiaceae; Salviae miltiorrhizae radix et rhizoma], *Lepidium apetalum* Willd. [Brassicaceae; Lepidii semen], *Alisma plantago-aquatica* L. [Alismataceae; Alismatis rhizoma], *Polygonatum odoratum* (Mill.) Druce [Asparagaceae; Polygonati odorati rhizoma], *Cinnamomum cassia* (L.) J.Presl [Lauraceae; Cinnamomi ramulus], *Carthamus tinctorius* L. [Asteraceae; Carthami flos], *Periploca sepium* Bunge [Apocynaceae; Periplocae cortex]	4 capsules, (0.3 g/capsule), tid, po	Y-prepared according to NMPA: Z20040141	-	NR
Liqi Huoxue Dripping pills	Guizhou Minzu Pharmaceutical Co., Ltd.	*Litsea cubeba* (Lour.) pers. [Lauraceae; Litseae cubebae fructus], *Blumea balsamifera* (L.) DC. [Asteraceae; L-Borneolum], *Ligusticum chuanxiong* S.H.Qiu, Y.Q.Zeng, K.Y.Pan, Y.C.Tang and J.M.Xu [Apiaceae; Chuanxiong rhizoma], *Allium macrostemon* Bunge [Amaryllidaceae; Allii macrostemonis bulbus]	10 pills, (25 mg/pill), tid, po	Y-prepared according to NMPA: Z20120037	-	NR
Dengzhan Shengmai Capsules	Yunnan Shengwugu Pharmaceutical Co., Ltd.	*Erigeron breviscapus* (Vant.) Hand.-Mazz. [Asteraceae; Erigerontis herba], *Panax ginseng* C.A.Mey. [Araliaceae; Ginseng radix et rhizoma], *Schisandra chinensis* (Turcz.) Baill. [Schisandraceae; Schisandrae chinensis fructus], *Ophiopogon japonicus* (L. f.) Ker Gawl. [Asparagaceae; Ophiopogonis radix]	2 capsules, (0.18 g/capsule), tid, po	Y-prepared according to NMPA: Z20026439	-	NR
Yuxintong Capsules	Jilin Aodong Group Dalian Pharmaceutical Co., Ltd.	*Corydalis yanhusuo* (Y.H.Chou and Chun C.Hsu) W.T.Wang ex Z.Y.Su and C.Y.Wu [Papaveraceae; Corydalis rhizoma], *Panax ginseng* C.A.Mey. [Araliaceae; Ginseng radix et rhizoma rubra], *Panax notoginseng* (Burkill) F.H.Chen ex C.H.Chow [Araliaceae; Notoginseng radix et rhizoma]	3 capsules, (0.33 g/capsule), tid, po	Y-prepared according to NMPA: Z20020089	160,109/170,108	NR
Yixinshu tablets	Guizhou Xinbang Pharmaceutical Co., Ltd.	*Panax ginseng* C.A.Mey. [Araliaceae; Ginseng radix et rhizoma], *Ophiopogon japonicus* (Thunb.) Ker Gawl. [Asparagaceae; Ophiopogonis radix], *Schisandra chinensis* (Turcz.) Baill. [Schisandraceae; Schisandrae chinensis fructus], *Astragalus membranaceus* Fisch. ex Bunge [Fabaceae; Astragali radix], *Salvia miltiorrhiza* Bunge [Lamiaceae; Salviae miltiorrhizae radix et rhizoma], *Ligusticum chuanxiong* S.H.Qiu, Y.Q.Zeng, K.Y.Pan, Y.C.Tang and J.M.Xu [Apiaceae; Chuanxiong rhizoma], *Crataegus pinnatifida* Bunge [Rosaceae; Crataegi fructus]	3 tablets, (0.6 g/tablet), tid, po	Y-prepared according to NMPA: Z20090491	-	NR
Guanxinning tablets	Zhengda Qingchunbao Pharmaceutical Co., Ltd.	*Salvia miltiorrhiza* Bunge [Lamiaceae; Salviae miltiorrhizae radix et rhizoma], *Ligusticum chuanxiong* S.H.Qiu, Y.Q.Zeng, K.Y.Pan, Y.C.Tang and J.M.Xu [Apiaceae; Chuanxiong rhizoma]	3-4 tablets, (0.38 g/tablet), tid, po	Y-prepared according to NMPA: Z20150028	-	NR

* Non-botanical drug names comply with the Chinese Pharmacopoeia (2025 edition). Artificial Moschus and Artificial Bovis Calculus are approved synthetic substitutes. Storage conditions follow manufacturer’s specifications: cool, dry place. NMPA, national medical products administration of china.

### 2.2 Search strategy

Two investigators independently conducted a systematic search of relevant databases in Chinese and English. Sources included international databases such as PubMed, the Cochrane Library, Embase, and Web of Science, as well as Chinese databases including CNKI, the VIP Journal Integration Platform, and the Wanfang Data Knowledge Service Platform. The search was updated until November 2025. To ensure a comprehensive retrieval of studies, additional records were identified by examining conference proceedings, manually checking the reference lists of pivotal publications, and seeking input from field specialists. The search strategy was constructed according to the PICOS framework, integrating both controlled vocabulary (MeSH terms) and free-text keywords. Search terms were logically combined using the Boolean operators AND and OR An example search string is as follows: ((“Microvascular Angina” [Mesh] OR (“syndrome X” OR “coronary microvascular disease” OR “coronary microvascular diseases” OR “coronary microvascular disease”): ti, ab) AND ((“Medicine, Chinese Traditional” OR “Chinese patent drug” OR capsule OR tablet OR pill OR powder OR “oral liquid” OR granule): ti, ab, kw) AND ((“Randomized Controlled Trial” OR “controlled clinical trial” OR randomized OR placebo OR “drug therapy” OR randomly OR trial OR groups): ti, ab). The complete search strategy is provided in Supplementary Appendix S2.

### 2.3 Study selection and data extraction

The study selection process involved initial screening, re-screening, and qualitative evaluation. Two researchers independently reviewed the literature using EndNote, automatically and manually removing duplicates. Studies were screened based on predefined inclusion and exclusion criteria, and ineligible studies were excluded. Full-text reviews were conducted to eliminate further studies that did not report relevant outcomes or follow the specified interventions. Any discrepancies were resolved through discussion or by consulting a supervisor. Data extraction was conducted using a predefined form that categorized data into the following: ① general study information (author, publication year, sample size, participant age, disease duration); ② research methods (randomization, allocation concealment, blinding, selective reporting); ③ intervention details (treatment/control groups, duration); and ④ outcome measures (IMR, CFR, cTFC, total effective rate, LVEF, NO, hs-CRP and ET-1 levels, adverse events).

### 2.4 Risk of bias and quality assessment

The methodological quality of each included RCT was independently appraised by two reviewers using the Cochrane Risk of Bias tool version 2 (RoB 2) (Sterne et al., 2019), which evaluates five distinct domains: (1) the randomisation process; (2) deviations from intended interventions; (3) missing outcome data; (4) measurement of the outcome; and (5) selection of the reported results. Each trial was classified as having low risk of bias if rated low across all domains; some concerns if any domain raised reservations without reaching high-risk thresholds; or high risk if any single domain indicated high bias or multiple domains generated substantial uncertainty regarding result validity. Discrepancies between reviewers were resolved by consensus or, where necessary, third-party adjudication. The certainty of evidence derived from the NMA was subsequently evaluated using the Confidence in Network Meta-Analysis (CINeMA) framework, which assesses confidence across six complementary domains: within-study bias, reporting bias, indirectness, imprecision, heterogeneity, and incoherence (Nikolakopoulou et al., 2020; Papakonstantinou et al., 2020). Sensitivity analyses were planned to examine the robustness of the primary outcomes following the exclusion of trials with a high risk of bias or some concerns.

### 2.5 Data synthesis and analysis

The NMA was conducted using a frequentist framework, with StataMP 18.0 employed for data analysis and visualization. For dichotomous outcomes, treatment effects were expressed as relative risks (RR) with 95% confidence intervals (CI), while odds ratios (ORs) with 95% CIs were used for adverse event rates. Continuous outcomes were synthesized using mean differences (MDs) and their 95% CIs. Heterogeneity was assessed using τ^2^ values, categorized as low (<0.04), low-to-moderate (0.04–0.16), moderate-to-high (0.16–0.36), or high (>0.36) (Rhodes et al., 2015; Huang et al., 2025). The τ^2^ value was assumed constant across all comparisons, and a correlation of 0.5 was specified for the between-study covariance matrix. Local consistency between direct and indirect evidence within closed loops was examined using the node-splitting method, while global inconsistency across the network was assessed via a design-by-treatment interaction model. A network plot was generated to illustrate the comparisons among interventions, with nodes representing treatments (sized by total patient sample size) and edges denoting direct comparisons (thickness proportional to the number of studies). Results are presented in a league table of all pairwise comparisons. Treatments were ranked by calculating SUCRA scores, ranging from 0 to 1, with higher scores indicating a more favorable ranking. Potential publication bias was evaluated by visual inspection of funnel plots. Pre-specified sensitivity analyses were conducted for primary outcomes by sequentially excluding: (1) studies rated as high risk of bias in the randomization domain; and (2) small-sample studies (n < 30 per arm).

## 3 Results

### 3.1 Literature search results

**FIGURE 1:**
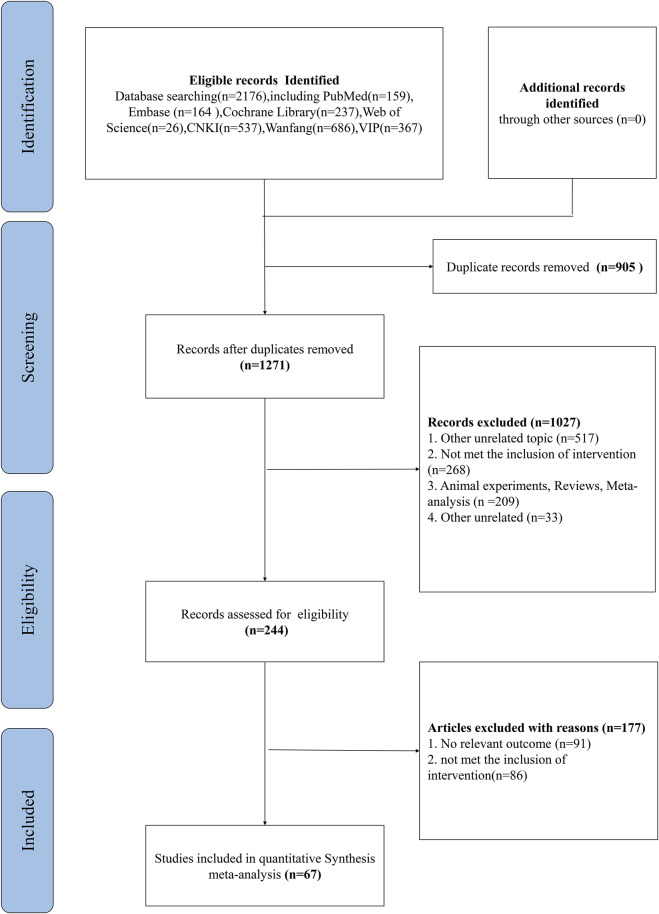
Flowchart of literature search and selection process.

The NMA began with a comprehensive database search, identifying 2,176 articles, which were then managed in EndNote. From these, 905 duplicate entries were removed. Next, titles and abstracts were reviewed, leading to the exclusion of another 1,027 studies. Specifically, these comprised 517 on unrelated topics, 209 reviews, animal studies, meta-analyses, or experimental designs, 268 without the required interventions, and 33 other irrelevant studies. Subsequently, full texts were evaluated using predefined inclusion and exclusion criteria: 91 were excluded for lacking outcome measures, and 86 for not meeting intervention criteria. Ultimately, 67 RCTs were included (Figure 1).

### 3.2 Included study characteristics

This NMA included 67 Chinese RCTs published between 2011 and 2025, involving 6,139 participants (Song et al., 2011; Zhang et al., 2013; Peng and Lu, 2014; Wang et al., 2014; Ge et al., 2015; Wang and Tan, 2015; Zhou, 2015; Chen et al., 2016; Zhang et al., 2016; Cai, 2017; Li et al., 2017; Liu et al., 2017; Qin et al., 2017; Yang and Zhou, 2017; Jin, 2018; Wei, 2018; Wu et al., 2018; Yan et al., 2018; Chen et al., 2019; Feng, 2019; Jia et al., 2019; Li, 2019; Liang et al., 2019; Wang et al., 2019; Wu et al., 2019; Xue et al., 2020; Zhang, 2020; Zhang and Chen, 2020; Fu, 2021; Gong et al., 2021; He et al., 2021; Kang et al., 2021; Li, 2021; Liu, 2021; Shen et al., 2021; Shen, 2021; Zhang et al., 2021; Zhao et al., 2021; Bai et al., 2022; Chen et al., 2022; Chen and Shao, 2022; Sun et al., 2022; Wang and Li, 2022; Wei et al., 2022; Qin et al., 2023; Yang and Ren, 2023; Zhao D. et al., 2023; Chen et al., 2024; Han et al., 2024; Lai, 2024; Li et al., 2024; Liu, 2024; Peng et al., 2024; Sun et al., 2024; Sun, 2024; Wang B. et al., 2024; Wang H. et al., 2024; Zhao et al., 2024; Zheng and Wu, 2024; Zhou et al., 2024; Kong et al., 2025; Lai, 2025; Li and Liu, 2025; Li N. et al., 2025; Liu R. et al., 2025; Liu Y. et al., 2025; Zhang, 2025). The study population was divided into two groups: an intervention group of 3,082 participants and a control group of 3,057 participants. Of the 67 trials, 4 did not report participants’ ages. The mean age of participants in the remaining 63 RCTs ranged from 41 to 70 years. Individual trial sample sizes ranged from 40 to 200 participants, and treatment durations ranged from 4 to 48 weeks. Details of all included studies are provided in Supplementary Appendix S3, Supplementary Table S3.1.

A total of 19 CCPPs were included in this study. Details on the TCM functions, ingredients, metabolites, pharmacological effects, and regulatory information (including approval and batch numbers) for these CCPPs are available in Table 1; Supplementary Appendix S3, Supplementary Table S3.2.

### 3.3 Risk of bias, and certainty of evidence

**FIGURE 2:**
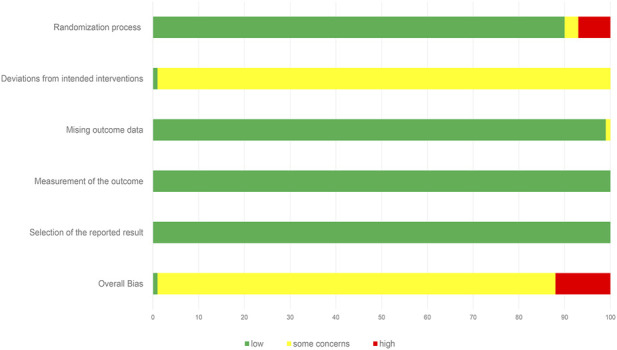
Risk of bias item across all included studies.

Risk of bias was assessed for all 67 RCTs using the Cochrane RoB 2.0 tool (Figure 2; Supplementary Table S5). For the randomisation process (D1), 60 trials (89.5%) were rated as low risk, reflecting adequate sequence generation, while 7 (10.5%) raised some concerns due to insufficient reporting of allocation concealment. Regarding deviations from intended interventions (D2), only one trial (1.4%) achieved low risk. In contrast, the remaining 66 studies (98.6%) were rated as having some concerns or high risk, attributable to the open-label design inherent to Chinese patent medicine administration, which precludes blinding of participants and investigators. Missing outcome data (D3) were rated as low risk in 66 trials (98.5%), indicating robust handling of attrition. Measurement of the outcome (D4) was rated as low risk across all 67 trials (100%). For the selection of the reported result (D5), 61 trials (91%) were rated as low risk, while 6 trials (9%) were rated as having some concerns, reflecting the potential risk of selective reporting due to the lack of publicly available registered protocols in some subset of studies. Overall, 56 trials (86.5%) were judged to have some concerns, and 8 (11.9%) were judged to be high risk; only one trial (1.4%) was judged to be low risk across all domains. These limitations—predominantly inadequate blinding due to the nature of herbal interventions and incomplete reporting of randomisation methods—are recognized as characteristic of open-label trials in the traditional Chinese medicine context and should be considered when interpreting the network estimates. The CINeMA framework was used to evaluate the certainty of evidence for each comparison; most pairwise comparisons were graded as low to moderate confidence, primarily downgraded due to risk of bias in individual studies and, to a lesser degree, imprecision (Appendix 10).

### 3.4 Consistency, heterogeneity, and publication bias

Transitivity was preliminarily evaluated by comparing key confounding factors across included studies, such as baseline age, disease course, sample size and overall risk of bias, to roughly ensure the comparability of main effect modifiers among different treatment groups. The assessment of transitivity was performed in general accordance with our pre-specified study protocol, with no obvious analytical deviations. All evidence networks satisfied the transitivity assumption, thereby supporting the rationality of indirect comparisons to a certain extent (Supplementary Appendix S10, Supplementary Table S10.1). Notably, no closed treatment loops were observed in the current evidence network, which may make local inconsistency assessment difficult to conduct in this analysis. Global inconsistency was assessed via the design-by-treatment interaction model, which demonstrated relatively good concordance between direct and indirect evidence across all treatment comparisons, with no statistically significant inconsistency identified (p > 0.05). The evaluation of global consistency was generally consistent with the pre-defined analytical protocol. For heterogeneity, τ^2^ estimates indicated low-to-moderate variance for most outcomes, which may provide partial support for the reliability of pooled effect estimates (Supplementary Appendix S6, Supplementary Table S6). Visual inspection of funnel plots revealed no substantial asymmetry across primary outcomes, suggesting a relatively low possibility of obvious publication bias (Appendix 11).

### 3.5 Primary outcomes

#### 3.5.1 IMR

**FIGURE 3:**
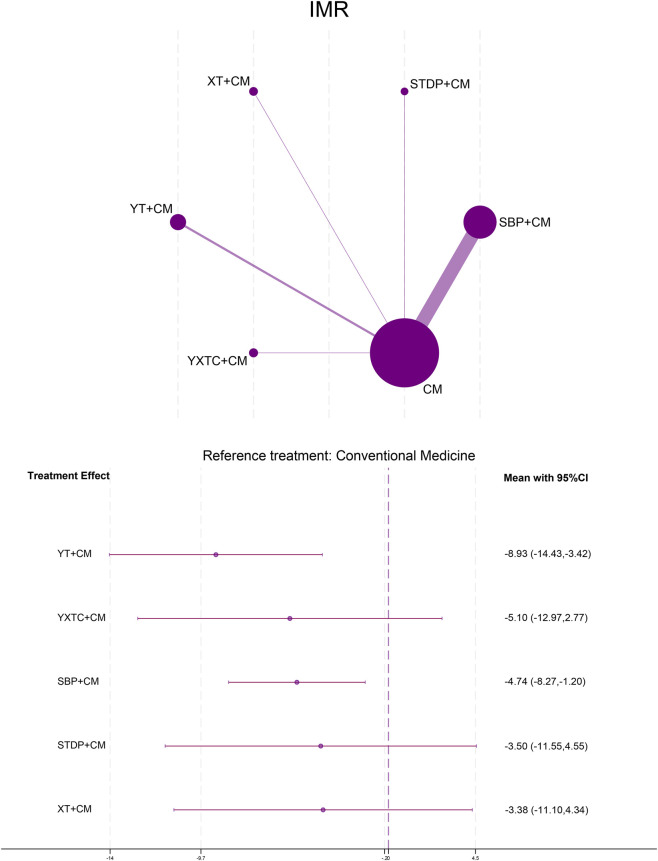
Network meta-analysis results for the index of microcirculatory resistance (IMR). Evidence network plot for IMR. Nodes represent individual interventions, with node size proportional to the total number of randomized patients. Edges represent direct comparisons, with edge thickness proportional to the number of contributing trials. Forest plot of pooled Mean differences (MD) with 95% confidence intervals (CI) for each CCPP-based intervention versus conventional medicine therapy alone.

This NMA included 9 RCTs involving 689 participants to assess reductions in IMR. The network plot showed direct comparisons between CCPP + CM combination regimens and CM alone, with node size proportional to the number of participants per intervention and line thickness reflecting the number of trials directly comparing the two treatments. The forest plot showed that, compared with CM monotherapy, some CCPP-containing combination regimens achieved significant reductions in IMR. YT + CM showed a relatively favorable probabilistic reduction (MD = −8.93, 95% CI: 14.43 to −3.42; SUCRA = 88.4%, low certainty of evidence), followed by SBP + CM (MD = −4.74, 95% CI: 8.27 to −1.20; SUCRA = 56.3%, low certainty of evidence) (Figure 3). The league table showed no statistically significant difference in the indirect comparisons between the various intervention regimens. Given the lack of significant differences in indirect comparisons and the low certainty of evidence, these SUCRA rankings only reflect cumulative probability trends and cannot be regarded as definitive superior effects. According to the CINeMA framework, the overall certainty of evidence for the IMR outcome was mainly rated as low (Supplementary Appendix S8, Supplementary Table S8.1; Supplementary Appendix S9; Supplementary Table S9.1; Supplementary Appendix S10; Supplementary Table S10.2).

#### 3.5.2 CFR

**FIGURE 4:**
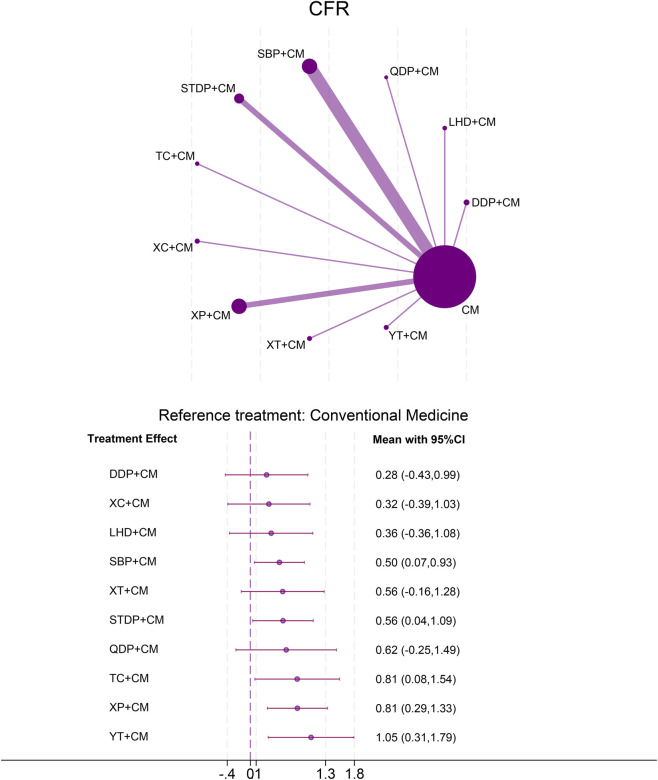
Network meta-analysis results for coronary flow reserve (CFR). Evidence network plot for CFR. Nodes represent individual interventions, with node size proportional to the total number of randomized patients. Edges represent direct comparisons, with edge thickness proportional to the number of contributing trials. Forest plot of pooled Mean differences (MD) with 95% confidence intervals (CI) for each CPM-based intervention versus conventional medicine therapy alone.

Regarding the outcome of CFR, this NMA synthesized data from 15 RCTs encompassing 1,485 participants. The forest plot showed that some of the 10 CCPP + CM combination regimens achieved statistically significant improvements in CFR compared with CM monotherapy. Among these, YT + CM achieved a higher SUCRA ranking for CFR improvement (MD = 1.05, 95% CI: 0.31 to 1.79; SUCRA = 85.0%, low certainty of evidence), followed by XP + CM (MD = 0.81, 95% CI: 0.29 to 1.33; SUCRA = 72.7%, low certainty of evidence), and TC + CM (MD = 0.81, 95% CI: 0.08 to 1.54; SUCRA = 70.2%, low certainty of evidence) (Figure 4). The league table showed no statistically significant difference in indirect comparisons between the various intervention regimens. Such hierarchical results should be interpreted with great caution, considering the low evidence certainty and non-significant indirect head-to-head comparisons. According to the CINeMA framework, the overall certainty of evidence for the CFR outcome was mainly rated as low (Supplementary Appendix S8, Supplementary Table S8.2; Supplementary Appendix S9; Supplementary Table S9.2; Supplementary Appendix S10; Supplementary Table S10.3).

#### 3.5.3 cTFC

**FIGURE 5:**
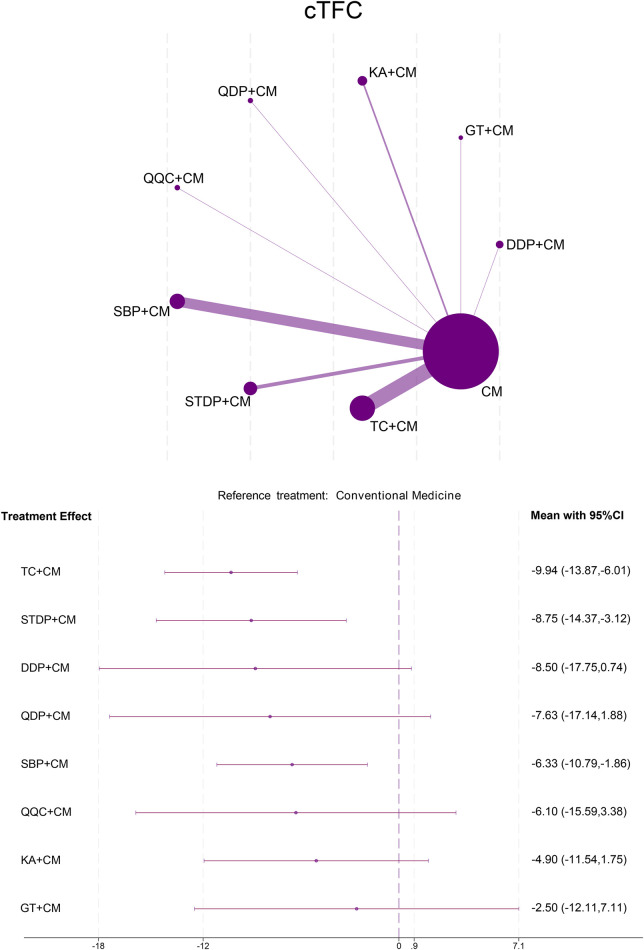
Network meta-analysis results for the corrected Thrombolysis in Myocardial Infarction frame count (cTFC). Evidence network plot for cTFC. Nodes represent individual interventions, with node size proportional to the total number of randomized patients. Edges represent direct comparisons, with edge thickness proportional to the number of contributing trials. Forest plot of pooled Mean differences (MD) with 95% confidence intervals (CI) for each CCPP-based intervention versus conventional medicine therapy alone.

Regarding cTFC reduction, 20 RCTs involving 1,513 participants were included in this NMA. The forest plot showed that 8 CCPP + CM combination regimens exhibited a trend toward reduced cTFC, but only 3 intervention regimens reached statistical significance. Among them, TC + CM presented a relatively high probabilistic rank in cTFC lowering (MD = −9.94, 95% CI: 13.87 to −6.01, SUCRA = 79.5%, low certainty of evidence). Similarly, STDP + CM (MD = −8.75, 95% CI: 14.37 to −3.12, SUCRA = 69.5%, low certainty of evidence) also exhibited a significant reduction effect (Figure 5). The league table showed no statistically significant difference in the indirect comparisons between the different intervention regimens. Limited by low evidence quality and non-significant indirect comparisons, these SUCRA rankings do not indicate reliable superiority across different regimens. According to the CINeMA framework, the overall certainty of evidence for the cTFC outcome was mainly rated as low (Supplementary Appendix S8, Supplementary Table S8.3; Supplementary Appendix S9; Supplementary Table S9.3; Supplementary Appendix S10; Supplementary Table S10.4).

### 3.6 Secondary outcomes

#### 3.6.1 Total effective rate

To assess the clinical efficacy of different CCPP + CM regimens in the treatment of CMVD, this NMA of the total effective rate was conducted, including 35 eligible RCTs enrolling 3,385 participants. The network plot showed that all 14 CCPP + CM combination regimens were directly compared with CM monotherapy, with the diameter of circular nodes representing the sample size for each intervention and the thickness of connecting lines reflecting the number of RCTs included in each pairwise comparison. Forest plot analysis demonstrated that the majority of CCPP + CM regimens achieved a significantly higher total clinical effectiveness rate compared with CM monotherapy (Supplementary Appendix S7, Supplementary Figure S7.1). Among these regimens, TC + CM obtained the highest SUCRA score in cumulative ranking analysis (RR = 1.46, 95% CI: 1.26 to 1.70; SUCRA = 92.2%, moderate certainty of evidence), followed by GT + CM (RR = 1.40, 95% CI: 1.07 to 1.83; SUCRA = 79.8%, moderate certainty of evidence) and LHD + CM (RR = 1.30, 95% CI: 1.04 to 1.62; SUCRA = 64.7%, moderate certainty of evidence). Notably, most indirect comparisons across regimens remained non-significant; hence, these probabilistic rankings require prudent clinical interpretation (Supplementary Appendix S8, Supplementary Table S8.4; Supplementary Appendix S9; Supplementary Table S9.4; Supplementary Appendix S10; Supplementary Table S10.5).

#### 3.6.2 LVEF

This NMA included 16 RCTs involving 1,441 participants and reported LVEF data. Relative to CM monotherapy, only a subset of CCPP + CM regimens achieved a statistically significant improvement in LVEF (Supplementary Appendix S7, Supplementary Figure S7.2). STDP + CM showed a favorable SUCRA profile for LVEF improvement (MD = 10.99, 95% CI: 5.42 to 16.55; SUCRA = 94.9%), followed by SSP + CM (MD = 5.91, 95% CI: 0.21 to 11.60; SUCRA = 65.0%, low certainty of evidence). In view of low evidence certainty and insignificant indirect comparative results, such ranking outcomes are exploratory rather than conclusive. Further details on SUCRA and indirect comparisons were provided in Supplementary Appendix S8 (Supplementary Table S8.5) and Supplementary Appendix S9 (Supplementary Table S9.5).

#### 3.6.3 Serum hs-CRP levels

To assess the effects of various CCPP-based combination regimens on serum hs-CRP levels in patients with CMVD, this NMA of 29 eligible RCTs enrolling 2,724 participants was conducted, all of which reported hs-CRP outcomes. The results indicated that, compared with CM monotherapy, most CCPP plus CM regimens achieved a statistically significant reduction in serum hs-CRP levels (Supplementary Appendix S7, Supplementary Figure S7.3). Among these regimens, YXTC + CM ranked higher in the SUCRA cumulative ranking for reducing hs-CRP (MD = −3.37, 95% CI: 4.58 to −2.17; SUCRA = 93.4%), followed by SBP + CM (MD = −1.79, 95% CI: 3.36 to −0.22; SUCRA = 84.5%) and STDP + CM (MD = −1.48, 95% CI: 2.67 to −0.29; SUCRA = 83.0%), all of which exhibited statistically significant hs-CRP-lowering effects versus CM monotherapy. Still, the absence of significant differences in indirect comparisons restricts the robust differentiation between different CCPP regimens. Further details on SUCRA and indirect comparisons are provided in Supplementary Appendix S8 (Supplementary Table S8.6) and Supplementary Appendix S9 (Supplementary Table S9.6).

#### 3.6.4 Serum ET-1 levels

To evaluate the effects of different CCPP-containing treatment regimens on serum ET-1 levels in CMVD patients, this NMA of 30 eligible RCTs enrolling 3,048 participants was conducted, all of which reported serum ET-1 levels as an outcome. Compared with CM monotherapy, most CCPP plus CM regimens exhibited a significantly greater reduction in serum ET-1 levels (Supplementary Appendix S7, Supplementary Figure S7.4). Among these regimens, DDP + CM achieved a superior probabilistic SUCRA rank for ET-1 reduction (MD = −36.33, 95% CI: 53.83 to −18.83, SUCRA = 91.4%), followed by STDP + CM (MD = −34.51, 95% CI: 54.32 to −14.70, SUCRA = 88.5%) and XC + CM (MD = −31.82, 95% CI: 52.19 to −11.45, SUCRA = 85.1%), all of which achieved a statistically significant reduction in ET-1 levels relative to CM monotherapy. These probabilistic rankings should not be overinterpreted, given low evidence certainty and non-significant indirect comparisons across interventions. Further details on SUCRA and indirect comparisons are provided in Supplementary Appendix S8 (Supplementary Table S8.7) and Supplementary Appendix S9 (Supplementary Table S9.7).

#### 3.6.5 Serum NO levels

To evaluate the effect of different CCPP-containing regimens on serum NO levels in patients with CMVD, this NMA, including 28 RCTs enrolling 2,751 participants, was conducted, all of which reported serum NO level outcomes. Compared with CM monotherapy, multiple CCPP plus CM regimens significantly elevated serum NO levels (Supplementary Appendix S7, Supplementary Figure S7.5). Among these, DDP + CM displayed a higher cumulative SUCRA ranking (MD = 31.55, 95% CI: 10.49 to 52.60; SUCRA = 88.5%), followed by QDP + CM (MD = 22.47, 95% CI: 5.20 to 39.74; SUCRA = 73.4%) and YXTC + CM (MD = 19.24, 95% CI: 1.34 to 37.15; SUCRA = 66.3%). Due to limited evidence quality and insignificant indirect head-to-head results, these ranking findings remain exploratory. Further details on SUCRA and indirect comparisons are provided in Supplementary Appendix S8 (Supplementary Table S8.8) and Supplementary Appendix S9 (Supplementary Table S9.8).

#### 3.6.6 Adverse events

To assess the safety of various CCPP-based therapies for CMVD, a network meta-analysis of adverse event data from 21 RCTs enrolling 1,996 participants was conducted. The network plot demonstrated that all 12 CCPPs combination regimens were directly compared with CM. Forest plot analysis revealed that SBP + CM (RR = 0.33, 95% CI: 0.13 to 0.84, SUCRA = 82.6%) and YXTC + CM (RR = 0.38, 95% CI: 0.16 to 0.91, SUCRA = 79.8%) significantly reduced the risk of adverse events compared with CM alone.

Common adverse events reported across trials encompassed a spectrum of generally mild manifestations, such as gastrointestinal symptoms, dizziness, palpitations, rash, headache, and other non-specific complaints. The majority of these events were characterized as mild in severity and self-limiting in nature. Events of greater clinical concern were infrequent, and their causal relationship to the study preparations could not be definitively established based on the available data. Detailed safety data, including event types and frequencies in both intervention and control groups, are presented in Appendix 13. These findings suggest that combination regimens containing CCPPs appear to be generally well-tolerated in patients with CMVD, though the interpretation of safety outcomes should consider the limitations in event reporting across trials (Supplementary Appendix S7; Supplementary Figure S7.6; Supplementary Appendix S8; Supplementary Table S8.9; Supplementary Appendix S9; Supplementary Table S9.9; Supplementary Appendix S13).

To enhance result transparency and clinical practicality, we have summarized the top three regimens for all evaluated outcomes based on SUCRA metrics in Supplementary Table S14 (Supplementary Appendix S14). Interventions with small sample sizes, limited available studies or wide confidence intervals are clearly labeled as insufficient evidence, to avoid misinterpretation and ensure reasonable and evidence-based clinical decision-making.

### 3.7 Sensitivity analyses

Based on the inclusion criteria, the studies divided into two groups: the treatment group received CCPPs in combination with CM, while the control group was treated with CM alone. To test the robustness of the primary outcome measure, we conducted sensitivity analyses excluding RCTs with a sample size of fewer than 30 per group. None of the studies found a significant effect on outcome. As shown in Appendix 12, the results of sensitivity analyses were consistent with the primary results, confirming their robustness.

## 4 Discussion

### 4.1 Summary of findings

This NMA was conducted to systematically assess the comparative efficacy of 19 CCPPs as adjuncts to conventional Biomedicine for CMVD, pooling data from 67 RCTs involving 6,139 patients. The findings suggest that specific CCPPs-CM combinations outperformed CM monotherapy across multiple key clinical outcomes for CMVD, with notable heterogeneity in the efficacy of different CCPPs for distinct therapeutic targets. Yixinshu Tablets (YT)+CM appear to be a relatively favorable option for improving CFR and reducing IMR, while Tongxinluo Capsule (TC)+CM shows potential advantages in increasing the total effective rate and lowering cTFC. Shexiang Tongxin Dripping Pills (STDP)+CM may provide notable benefits for LVEF improvement. Meanwhile, Yindan Xinnao Tong Capsules (YXTC)+CM and Danshen Dripping Pills (DDP)+CM tend to exert preferable modulatory effects on inflammatory and endothelial biomarkers, including hs-CRP, ET-1, and NO. Collectively, these results indicate that adjunctive CCPPs treatment may offer multiple therapeutic benefits for CMVD management, providing a hierarchical evidence base for personalized CCPPs selection in clinical practice.

### 4.2 Comparison with existing literature

Within the framework of TCM, CMVD is classified under “chest bi” (thoracic obstruction) and “heart pain.” The predominant clinical phenotypes involve Qi deficiency coupled with blood stasis or the accumulation of phlegm and stasis (Yu et al., 2022). The CCPPs evaluated in this study are primarily formulated to invigorate blood circulation and replenish Qi to restore patency—principles that resonate closely with the underlying pathophysiology of CMVD. Contemporary pharmacological evidence suggests that these multi-metabolites agents exert synergistic effects across diverse molecular targets and signaling pathways. By modulating the vascular endothelium, suppressing inflammatory cascades, attenuating oxidative stress, and stimulating angiogenesis (Yang et al., 2022; Yang et al., 2023; Wang Y. et al., 2024), CCPPs may help improve the complex pathological changes of CMVD, and might compensate for the limited therapeutic effect of single conventional biomedical treatment. In this context, an NMA was conducted to systematically assess the comparative efficacy and safety of various CCPPs as adjuncts to CM versus CM alone. The primary objective was to evaluate their impact on microcirculatory status, endothelial function, and clinical prognoses, thereby generating robust evidence-based insights to refine integrated treatment strategies for CMVD. This study presents several potential methodological improvements compared with previous relevant meta-analyses (Wang et al., 2025). Notably, the present analysis is substantially broader in scope: whereas the prior study synthesized data from 9 CCPPs across 39 randomized controlled trials (RCTs; n = 3,240), this work encompasses 19 CCPPs from 67 RCTs (n = 6,139), supported by a more contemporary literature cutoff and a more exhaustive search string. Furthermore, the rigorous Confidence in CINeMA framework was employed to evaluate the certainty of evidence. Regarding outcome selection, this study provides a more multidimensional assessment, beyond IMR and CFR, with cTFC, total effective rate, and LVEF integrated as key endpoints. Although certain inconsistencies in efficacy ranking exist between different studies, such differences may stem from variations in inclusion criteria, evidence network structure and heterogeneity control methods. Therefore, the updated efficacy ranking in this study might offer more clinically meaningful references for the individualized management of CMVD.

### 4.3 Modulatory impact of CCPPs on coronary microvascular function

CMVD remains the pathophysiological hallmark of myocardial ischemia in the absence of obstructive coronary artery disease. To rigorously quantify the therapeutic impact of CCPPs on microcirculatory health, this study utilized CFR, IMR, and cTFC as primary endpoints. CFR, defined as the ratio of hyperemic to basal coronary flow, offers a global assessment of vasodilatory capacity across both epicardial and microvascular compartments (Aun et al., 2022). In contrast, IMR provides a highly specific, pressure-wire-based quantification of microvascular resistance that remains independent of epicardial stenosis severity (Wang D. et al., 2024). Complementing these, cTFC serves as a reproducible angiographic surrogate for coronary flow velocity, where higher frame counts are robustly associated with impaired myocardial perfusion and microvascular rarefaction (Gibson et al., 1996; Subban and Mullasari, 2013).

These microcirculatory indicators not only assist clinical diagnosis, but also may correlate with long-term prognosis. Impaired CFR is strongly predictive of all-cause mortality and recurrent ischemic events, particularly in steering risk stratification and therapeutic interventions for patients with myocardial infarction with non-obstructive coronary arteries (MINOCA) (Kelshiker et al., 2022). Furthermore, in the setting of acute coronary syndromes managed with percutaneous coronary intervention (PCI), an elevated post-procedural IMR is a potent independent predictor of MACE, including heart failure hospitalization and target vessel failure (Eerdekens et al., 2024; Zhang et al., 2024). Evidence also suggests that elevated cTFC correlates with adverse prognoses in the MINOCA population (Mareai et al., 2023), while the synergistic application of CFR and IMR yields enhanced prognostic discrimination (Hong et al., 2023). Consequently, this multidimensional evaluation framework provides a scientifically rigorous basis for the efficacy assessments performed in our analysis.

The results of this network meta-analysis suggest distinct microcirculatory regulatory characteristics among different CCPPs. YT tended to show relatively favorable effects in improving CFR and reducing IMR. This efficacy likely stems from the synergistic action of metabolites—including ginsenosides, astragaloside IV, and tanshinones—which have been shown to preserve endothelial integrity, suppress pro-inflammatory cascades, and attenuate cellular apoptosis (Sun et al., 2017; Yu et al., 2022). Meanwhile, TC appears to have certain advantage in improving coronary flow and reducing cTFC. Mechanistic studies underscore its ability to enhance endothelium-dependent vasodilation and alleviate microvascular vasospasm through the PKA-eNOS signaling axis. Additionally, TC preserves microvascular structural stability and mitigates hyperpermeability via the PPAR-α/Angptl4 pathway, directly addressing the underlying drivers of the coronary slow-flow phenomenon (Qi et al., 2015; Qi et al., 2018).

### 4.4 Cardioprotective implications of CPMs in CMVD-related impairment

CMVD is inextricably linked to the pathophysiology of left ventricular (LV) diastolic impairment and serves as a critical driver in the continuum of heart failure (Toya et al., 2023). This clinical synergy has been robustly characterized in the literature. For example, Paolisso et al. found an inverse association between microcirculatory integrity and left ventricular diastolic function, and they noted that the presence of CMVD was consistently associated with poor diastolic parameters (Paolisso et al., 2024). In the context of STEMI, the prevalence of LV diastolic dysfunction is markedly higher in patients with microvascular compromise than in those without (Aldujeli et al., 2024). Moreover, evidence from hypertrophic cardiomyopathy cohorts underscores that microvascular rarefaction facilitates adverse ventricular remodeling and systolic decline, eventually predisposing patients with severe CMVD to end-stage heart failure (Olivotto et al., 2006).

In this NMA, the adjunctive use of Qi-supplementing and blood-invigorating CCPPs alongside CM showed certain potential trends in enhancing LVEF and overall clinical response, with STDP showing relatively preferable effects in this pooled analysis. These results resonate with established clinical benchmarks where STDP has been shown to significantly augment LVEF while attenuating N-terminal pro-B-type natriuretic peptide (NT-proBNP) levels (Wu et al., 2021). Mechanistically, the therapeutic breadth of STDP is attributed to its diverse metabolites—including tanshinones, salvianolic acids, and ginsenosides—which modulate pivotal signaling axes such as Dectin-1/Syk/IRF5 and VEGF/eNOS. By orchestrating anti-inflammatory responses, stimulating neoangiogenesis, and preserving endothelial homeostasis (Cui et al., 2023; Cui et al., 2024), STDP addresses the multifaceted pathological drivers of CMVD-related cardiac dysfunction, reinforcing its role as a viable evidence-based strategy for integrated cardiovascular care.

### 4.5 Modulatory effects of CCPPs on core pathophysiological pathways in CMVD

#### 4.5.1 Anti-inflammatory mechanisms

Chronic, low-grade inflammation is increasingly recognized as a central driver of CMVD pathogenesis and progression (Schindler and Bhandiwad, 2023). This sustained inflammatory milieu triggers the Fas/FasL and NLRP3 inflammasome pathways, perpetuating an inflammatory cascade that culminates in microvascular endothelial injury, microthrombogenesis, capillary rarefaction, and adverse vascular remodeling (Li et al., 2023; Guo et al., 2024). Clinical data corroborate this paradigm, demonstrating that CMVD patients exhibit markedly elevated circulating levels of pro-inflammatory cytokines—including C-reactive protein (CRP), IL-6, and TNF-α—which correlate positively with disease severity (Medina-Leyte et al., 2021). Notably, systemic inflammation, as quantified by hs-CRP, is a pivotal driver of coronary microvascular dysfunction (Bairey Merz et al., 2017).

In this NMA, treatment with YXTC was found to correlate with a relatively obvious decrease in hs-CRP levels among CMVD patients. This finding is in robust agreement with existing meta-analytical evidence: one synthesis of nine randomized controlled trials indicated that YXTC, as an adjunct to biomedical pharmacotherapy, not only significantly lowered hs-CRP but also ameliorated angina symptoms in CMVD cohorts (Yu et al., 2023). Furthermore, analogous meta-analyses in the context of acute ischemic stroke have validated the capacity of adjunctive YXTC to significantly downregulate serum hs-CRP, TNF-α, and IL-6 (Yang et al., 2024), underscoring a consistency in its anti-inflammatory profile across distinct vascular pathologies. Mechanistically, YXTC comprises a synergistic blend of metabolites—such as tanshinone IIA, salvianolic acid B, ginkgo flavonoids, scutellarin, and Panax notoginseng saponins (Shao et al., 2025). Together, these compounds suppress the NF-κB/NLRP3 inflammatory signaling axis and attenuate hs-CRP expression, thereby exerting a potent, multi-target modulatory effect to mitigate inflammation and restore coronary microcirculation (Zhao Y. et al., 2023).

#### 4.5.2 Endothelial cytoprotection

Vascular endothelial cells function as the critical regulatory nexus for maintaining the physiological vasomotor tone of coronary microvessels by precisely elaborating vasoactive mediators. Conversely, endothelium-dependent vasomotor dysfunction constitutes the fundamental pathological substrate of CMVD. Endothelial impairment directly suppresses eNOS activity, precipitating a deficit in NO synthesis and bioavailability. Concurrently, it markedly upregulates ET-1 production and secretion, ultimately culminating in profound endothelium-dependent vasodilatory failure (Amiri et al., 2004; Cui et al., 2014; Sabe et al., 2022). Given that emerging evidence establishes coronary endothelial and microvascular dysfunction as robust, independent predictors of adverse cardiovascular events, the preservation and longitudinal assessment of endothelial function carry immense prognostic value in the comprehensive management of CMVD (Brainin et al., 2018).

Our analysis indicates that DDP may exert potential advantages in improving endothelial function, reflected by decreased ET-1 and increased NO levels in relevant populations. These observations align closely with the established pharmacological profiles of Salvia miltiorrhiza formulations. The primary metabolites of Salvia miltiorrhiza, notably tanshinones and salvianolic acids, confer robust cardioprotection via a multidimensional mechanism encompassing antioxidant, anti-inflammatory, and direct endothelium-preserving actions (Hao et al., 2017; Li Q. et al., 2025). Consequently, these mechanistic insights provide a rigorous theoretical framework for the observed clinical efficacy of Salvia miltiorrhiza preparations in ameliorating microvascular endothelial dysfunction within the CMVD population.

### 4.6 Clinical implications

Based on the differentiated efficacy profiles of various CCPPs observed in this study, a targeted, phenotype-driven approach to medication selection may tentatively be considered in patients with CMVD, taking into account clinical manifestations, patterns of functional impairment, and comorbid conditions.

For patients with prominent coronary microcirculatory dysfunction, as evidenced by impaired coronary flow reserve and elevated microvascular resistance, Yixinshu Tablets might be regarded as one possible adjunctive treatment option. In CMVD patients with recurrent angina pectoris, slow coronary flow, and suboptimal symptomatic response to conventional therapy, Tongxinluo Capsule may show certain trends toward improving overall clinical response and ameliorating coronary slow flow status. For patients with concomitant cardiac systolic dysfunction and reduced left ventricular ejection fraction, Shexiang Tongxin Dripping Pills could be viewed as a tentative alternative adjunctive option.

Regarding vascular endothelial injury and chronic inflammatory activation commonly observed in CMVD, Yindan Xinnao Tong Capsules and Danshen Dripping Pills seem to display preliminary potential trends in improving endothelial function and reducing systemic inflammatory levels, which may make them hypothetically suitable for CMVD patients with long-term metabolic disorders or endothelial dysfunction. From a practical clinical decision-making perspective, all CCPPs included in this analysis should be applied only as adjunctive regimens on the basis of standardized conventional biomedicine, and are not intended to replace guideline-recommended antiplatelet, lipid-regulating, or anti-myocardial ischemia therapies.

Stratified medication selection based on clinical phenotype, degree of microcirculatory injury, and accompanying inflammatory status may help facilitate rational exploratory application of commercial Chinese polyherbal preparation in CMVD. These findings only provide preliminary exploratory reference for clinicians in developing individualized comprehensive treatment strategies.

### 4.7 Limitations

This NMA assessed 19 CCPPs as adjuncts to conventional medicine for CMVD, employing the CINeMA framework and adhering strictly to the PRISMA-NMA 2020 guidelines with complete PROSPERO registration to maintain methodological rigor. Several limitations warrant more explicit elaboration and full clarification. The included 67 RCTs exhibited prominent quality differences: only one trial was rated as low risk of bias, and 98.6% of included studies adopted an open-label design without effective blinding of participants and investigators. Such methodological defects may contribute to a high risk of performance and detection bias, which could potentially overestimate the clinical therapeutic effects of related interventions. Limited data for certain intervention nodes resulted in wide confidence intervals for some outcomes, thereby reducing the precision of effect estimates. Additionally, the network lacked closed loops, so all comparisons between individual CCPPs are primarily based on indirect evidence, which may weaken the reliability of pairwise comparative results. Although the search strategy encompassed both English and Chinese databases, all eligible studies were published in Chinese and lacked relevant international research data, resulting in obvious geographic and population bias and potentially restricting the external generalizability of our research conclusions. While funnel plots did not reveal substantial asymmetry, the prevalent publication of positive results suggests that residual publication bias cannot be excluded. In addition, the reporting of adverse events exhibited notable heterogeneity across the included trials. Certain studies provided aggregate event counts without detailed descriptions, while others did not explicitly report safety-related outcomes. Consequently, our capacity to fully characterize the comprehensive safety profile, including potential drug interactions, of the investigated preparations is constrained by the available data. Furthermore, considerable variation in treatment durations and the absence of long-term follow-up data limit the ability to draw definitive conclusions regarding the durability and safety of these integrative regimens.

Considering that most included trials presented some concerns or high risk of bias, and the overall certainty of current evidence was rated as low to moderate, the findings of this synthesis should be interpreted with sufficient caution. Despite these limitations, this synthesis consolidates current evidence. However, the findings require cautious interpretation, and validation through high-quality, internationally representative trials with rigorous methodology remains important.

## 5 Conclusion

In conclusion, this NMA synthesizes available data to suggest potential benefits CCPP + CM may bring favourable therapeutic trends in patients with CMVD. Specific formulations—notably YT, TC, and STDP—showed promising trends in ameliorating microcirculatory resistance, accelerating coronary flow, and preserving left ventricular systolic function. These observed clinical trends are hypothetically supported by potential pleiotropic effects, predominantly the attenuation of systemic inflammation and the restoration of endothelial homeostasis.

However, given the methodological constraints, geographic homogeneity, and paucity of longitudinal data inherent in the primary literature, these findings require prudent interpretation. The overall certainty of evidence in this analysis is low to moderate, and results should be regarded as exploratory and hypothesis-generating rather than definitive clinical conclusions. Ultimately, establishing the credible clinical role of CCPPs in the global management of CMVD awaits rigorous validation through large-scale, double-blind, and internationally representative randomized controlled trials with long-term follow-up. Until such evidence emerges, this analysis only offers tentative exploratory references for individualized and integrative therapeutic strategies for patients with CMVD.

## Data Availability

The original contributions presented in the study are included in the article/Supplementary Material, further inquiries can be directed to the corresponding author.

## Glossary

IMR: the index of microcirculatory resistance
CFR: coronary flow reserve
cTFC: corrected Thrombolysis in Myocardial Infarction frame count
LVEF: left ventricular ejection fraction
hs-CRP: high-sensitivity C-reactive protein
NO: nitric oxide
ET-1: endothelin-1
SBP: Shexiang Baoxin Pills
STDP: Shexiang Tongxin Dripping Pills
TC: Tongxinluo Capsule
QDP: Qishen Dropping Pills
XT: Xinkeshu Tablets
XP: Xinbao Pills
DDP: Danshen Dripping Pills
YXTC: Yindan Xinnao Tong Capsules
KA: Kuanxiong AeFrosol
XC: Xueshuantong Capsules
YSG: Yangxin Shengmai Granules
SSP: Shenxiang Suhe Pills
LHD: Lingbao Huxin Dan
QQC: Qili Qiangxin Capsules
LDP: Liqi Huoxue Dripping Pills
DSC: Dengzhan Shengmai Capsules
YC: Yuxintong Capsules
YT: Yixinshu Tablets
GT: Guanxinning Tablets
CM: conventional medicine
RCTs: randomized controlled trials
SUCRA: surface under the cumulative ranking curves
CINeMA: Confidence in Network Meta-Analysis
CMVD: coronary microvascular disease
CCPPs: commercial Chinese polyherbal preparation
