# Supplementary material for: Efficacy and safety of combining commercial Chinese polyherbal preparation with conventional medicine in the treatment of coronary microvascular disease: a systematic review and network meta-analysis
Source: Front Pharmacol. 2026 Jun 4;17:1846574. doi: 10.3389/fphar.2026.1846574 (PMC13275717; doi:10.3389/fphar.2026.1846574)
Supplement: Supplementary file 1 [file DataSheet1.pdf]

Supplementary Appendix

Efficacy and Safety of Combining Commercial Chinese polyherbal preparation with Conventional Medicine in the Treatment of Coronary Microvascular Disease: A Systematic Review and Network Meta-Analysis

Table of contents

Appendix 1: PRISMA NMA Checklist 3

Appendix 2: Search strategy 8

Appendix 3: Characteristics of included studies 14

Appendix 4: List of data extracted from the included randomized clinical trials 23

Appendix 5: Risk of bias of randomized clinical trials 24

Appendix 6: Evaluation of inconsistency and heterogeneity 26

Appendix 7: Network maps and forest plots of secondary outcomes 27

Appendix 8: SUCRA and cumulative probability plots 33

Appendix 9: League table of Summary Estimates for different Commercial Chinese polyherbal preparations combined with Conventional Medicine in the treatment of coronary microvascular disease, derived from the meta-analysis of 67 trials. 42

Appendix 10: CINeMA Assessment 51

Appendix 11: Funnel plots 66

Appendix 12: Table S12 Sensitivity analyses of primary outcomes 71

Appendix 13: Table S13. Safety and adverse events summary 72

Appendix 14: Table S14. Summary of top three interventions ranked by SUCRA for each clinical outcome *74*

Appendix 1: PRISMA NMA Checklist

Section/Topic	Item #	Checklist Item	Reported on Page #
**TITLE**			
Title	1	Identify the report as a systematic review *incorporating a network meta-analysis (or related form of meta-analysis).*	*Title*
**ABSTRACT**			
Structured summary	2	Provide a structured summary including, as applicable:<br>**Background:** main objectives<br>**Methods:** data sources; study eligibility criteria, participants, and interventions; study appraisal; and *synthesis methods, such as network meta-analysis.*<br>**Results:** number of studies and participants identified; summary estimates with corresponding confidence/credible intervals; *treatment rankings may also be discussed. Authors may choose to summarize pairwise comparisons against a chosen treatment included in their analyses for brevity.*<br>**Discussion/Conclusions:** limitations; conclusions and implications of findings.<br>**Other:** primary source of funding; systematic review registration number with registry name.	*Abstract*
**INTRODUCTION**			
Rationale	3	Describe the rationale for the review in the context of what is already known*, including mention of why a network meta-analysis has been conducted.*_	*1 Introduction*
Objectives	4	Provide an explicit statement of questions being addressed, with reference to participants, interventions, comparisons, outcomes, and study design (PICOS).	*1 Introduction*
**METHODS**			
Protocol and registration	5	Indicate whether a review protocol exists and if and where it can be accessed (e.g., Web address); and, if available, provide registration information, including registration number.	*2 Methods*
Eligibility criteria	6	Specify study characteristics (e.g., PICOS, length of follow-up) and report characteristics (e.g., years considered, language, publication status) used as criteria for eligibility, giving rationale. *Clearly describe eligible treatments included in the treatment network, and note whether any have been clustered or merged into the same node (with justification).*_	*2.1 PICOS criteria*

Information sources	7	Describe all information sources (e.g., databases with dates of coverage, contact with study authors to identify additional studies) in the search and date last searched.	*2.2 Search strategy*
Search	8	Present full electronic search strategy for at least one database, including any limits used, such that it could be repeated.	*2.2 Search strategy; Appendix 2: Search strategy*
Study selection	9	State the process for selecting studies (i.e., screening, eligibility, included in systematic review, and, if applicable, included in the meta-analysis).	*2.3 Study selection and data extraction*
Data collection process	10	Describe method of data extraction from reports (e.g., piloted forms, independently, in duplicate) and any processes for obtaining and confirming data from investigators.	*2.3 Study selection and data extraction*
Data items	11	List and define all variables for which data were sought (e.g., PICOS, funding sources) and any assumptions and simplifications made.	*2.1 PICOS criteria*
**Geometry of the network**	**S1**	Describe methods used to explore the geometry of the treatment network under study and potential biases related to it. This should include how the evidence base has been graphically summarized for presentation, and what characteristics were compiled and used to describe the evidence base to readers.	*2.5. Data synthesis and analysis*
Risk of bias within individual studies	12	Describe methods used for assessing risk of bias of individual studies (including specification of whether this was done at the study or outcome level), and how this information is to be used in any data synthesis.	*2.4 Risk of bias and quality assessment; 3.3 Risk of bias, and certainty of evidence*
Summary measures	13	State the principal summary measures (e.g., risk ratio, difference in means). *Also describe the use of additional summary measures assessed, such as treatment rankings and surface under the cumulative ranking curve (SUCRA) values, as well as modified approaches used to present summary findings from meta-analyses.*	*2.5. Data synthesis and analysis*
Planned methods of analysis	14	Describe the methods of handling data and combining results of studies for each network meta-analysis. This should include, but not be limited to:<br>• *Handling of multi-arm trials;*<br>• *Selection of variance structure;*<br>• *Selection of prior distributions in Bayesian analyses; and*<br>• *Assessment of model fit.*	*2.5. Data synthesis and analysis*
**Assessment of Inconsistency**	**S2**	Describe the statistical methods used to evaluate the agreement of direct and indirect evidence in the treatment network(s) studied. Describe efforts taken to address its presence when found.	*2.5. Data synthesis and analysis*
Risk of bias across studies	15	Specify any assessment of risk of bias that may affect the cumulative evidence (e.g., publication bias, selective reporting within studies).	*2.5. Data synthesis and analysis*
Additional analyses	16	Describe methods of additional analyses if done, indicating which were pre-specified. This may include, but not be limited to, the following:<br>• Sensitivity or subgroup analyses;	*2.5. Data synthesis and analysis*

		• Meta-regression analyses;<br>• *Alternative formulations of the treatment network; and*<br>• *Use of alternative prior distributions for Bayesian analyses (if applicable).*_	
**RESULTS†**			
Study selection	17	Give numbers of studies screened, assessed for eligibility, and included in the review, with reasons for exclusions at each stage, ideally with a flow diagram.	*3.1. Literature search results; Figure 1*
**Presentation of network structure**	**S3**	Provide a network graph of the included studies to enable visualization of the geometry of the treatment network.	*Figure 3; Figure 4; Figure 5; Appendix 7: Network maps and forest plots of secondary outcomes*
**Summary of network geometry**	**S4**	Provide a brief overview of characteristics of the treatment network. This may include commentary on the abundance of trials and randomized patients for the different interventions and pairwise comparisons in the network, gaps of evidence in the treatment network, and potential biases reflected by the network structure.	*3.5 Primary Outcomes; 3.6 Secondary Outcomes (all outcomes)*
Study characteristics	18	For each study, present characteristics for which data were extracted (e.g., study size, PICOS, follow-up period) and provide the citations.	*3.2. Included study characteristics; Appendix 3: Characteristics of included studies, Table S3.1: Baseline of characteristics of included studies*
Risk of bias within studies	19	Present data on risk of bias of each study and, if available, any outcome level assessment.	*3.3 Risk of bias, and certainty of evidence; Figure 2; Appendix 5: Risk of bias of randomized clinical trials, Table S5: Study-level risk of bias assessment using Cochrane risk of bias tool 2.0 for assessing risk of bias of randomized clinical trials.*

Results of individual studies	20	For all outcomes considered (benefits or harms), present, for each study: 1) simple summary data for each intervention group, and 2) effect estimates and confidence intervals. *Modified approaches may be needed to deal with information from larger networks.*	*3.5 Primary Outcomes; 3.6 Secondary Outcomes*
Synthesis of results	21	Present results of each meta-analysis done, including confidence/credible intervals. *In larger networks, authors may focus on comparisons versus a particular comparator (e.g. placebo or standard care), with full findings presented in an appendix. League tables and forest plots may be considered to summarize pairwise comparisons.* If additional summary measures were explored (such as treatment rankings), these should also be presented.	*3.5 Primary Outcomes; 3.6 Secondary Outcomes; Appendix 7: Network maps and forest plots of secondary outcomes; Appendix 8: SUCRA and cumulative probability plots; Appendix 9: League table of Summary Estimates for different Commercial Chinese polyherbal preparations combined with Conventional Medicine in the treatment of coronary microvascular disease, derived from the meta-analysis of 67 trials.*
**Exploration for inconsistency**	**S5**	Describe results from investigations of inconsistency. This may include such information as measures of model fit to compare consistency and inconsistency models, *P* values from statistical tests, or summary of inconsistency estimates from different parts of the treatment network.	*3.4 Consistency, Heterogeneity, and Publication Bias*
Risk of bias across studies	22	Present results of any assessment of risk of bias across studies for the evidence base being studied.	*3.4 Consistency, Heterogeneity, and Publication Bias*
Results of additional analyses	23	Give results of additional analyses, if done (e.g., sensitivity or subgroup analyses, meta-regression analyses*, alternative network geometries studied, alternative choice of prior distributions for Bayesian analyses,* and so forth).	*3.7 Sensitivity analyses*
**DISCUSSION**			
Summary of evidence	24	Summarize the main findings, including the strength of evidence for each main outcome; consider their relevance to key groups (e.g., healthcare providers, users, and policy-makers).	*4.1. Summary of findings*
Limitations	25	Discuss limitations at study and outcome level (e.g., risk of bias), and at review level (e.g., incomplete	*4.7 Limitations*

		retrieval of identified research, reporting bias). *Comment on the validity of the assumptions, such as transitivity and consistency. Comment on any concerns regarding network geometry (e.g., avoidance of certain comparisons).*	
Conclusions	26	Provide a general interpretation of the results in the context of other evidence, and implications for future research.	*5. Conclusion*
**FUNDING**			
Funding	27	Describe sources of funding for the systematic review and other support (e.g., supply of data); role of funders for the systematic review. This should also include information regarding whether funding has been received from manufacturers of treatments in the network and/or whether some of the authors are content experts with professional conflicts of interest that could affect use of treatments in the network.	*Funding; Conflict of interest*

Appendix 2: Search strategy

Table S2.1: Search strategy of PubMed

#	Searches
**1**	("Medicine, Chinese Traditional"[Mesh])
**2**	(Zhong Yi Xue[Title/Abstract] OR Chung I Hsueh[Title/Abstract] OR Hsueh, Chung I[Title/Abstract] OR Traditional Medicine, Chinese[Title/Abstract] OR Chinese Traditional Medicine[Title/Abstract] OR Traditional Chinese Medicine[Title/Abstract] OR Chinese Medicine, Traditional[Title/Abstract] OR Traditional Tongue Diagnosis[Title/Abstract] OR Tongue Diagnoses, Traditional[Title/Abstract] OR Tongue Diagnosis, Traditional[Title/Abstract] OR Traditional Tongue Diagnoses[Title/Abstract] OR Traditional Tongue Assessment[Title/Abstract] OR Tongue Assessment, Traditional[Title/Abstract] OR Traditional Tongue Assessments[Title/Abstract] OR Chinese patent drug[Title/Abstract] OR capsule[Title/Abstract] OR tablet[Title/Abstract] OR pill[Title/Abstract] OR powder[Title/Abstract] OR oral liquid[Title/Abstract] OR granule[Title/Abstract])
**3**	#1 OR #2
**4**	("Randomized Controlled Trial" [Publication Type]) OR (controlled clinical trial[Title/Abstract] OR randomized[Title/Abstract] OR placebo[Title/Abstract] OR drug therapy[Title/Abstract] OR randomly[Title/Abstract] OR trial[Title/Abstract] OR groups[Title/Abstract])
**5**	("Microvascular Angina"[Mesh])
**6**	("Angina, Microvascular"[Title/Abstract] OR "Cardiac Syndrome X"[Title/Abstract] OR "Syndrome X, Cardiac"[Title/Abstract] OR "Syndrome X, Angina"[Title/Abstract] OR "Angina Syndrome X"[Title/Abstract] OR "Angina Syndrome Xs"[Title/Abstract] OR "Syndrome Xs, Angina"[Title/Abstract] OR "X Syndrome, Angina"[Title/Abstract] OR "Angina X Syndrome"[Title/Abstract] OR "Angina X Syndromes"[Title/Abstract] OR "Syndrome, Angina X"[Title/Abstract] OR "coronary microvascular dysfunction"[Title/Abstract] OR "coronary microvascular disorder"[Title/Abstract] OR "coronary microvascular disease"[Title/Abstract] OR CMD[Title/Abstract] OR CMVD[Title/Abstract] OR "syndrome X"[Title/Abstract] OR MVA[Title/Abstract] OR "Coronary microcirculation disease"[Title/Abstract] OR "Angina Pectoris with Normal Coronary Arteriogram"[Title/Abstract] OR INOCA[Title/Abstract] OR "coronary microvascular diseases"[Title/Abstract] OR "coronary microvascular diseases"[Title/Abstract] OR "coronary microvascular diease"[Title/Abstract] OR "No-Reflow Phenomenon"[Title/Abstract] OR "No Reflow Phenomenon"[Title/Abstract] OR "Slow-Flow Phenomenon"[Title/Abstract] OR "Phenomenon,Slow-Flow"[Title/Abstract] OR "Slow Flow Phenomenon"[Title/Abstract] OR "Coronary microcirculation dysfunction"[Title/Abstract] OR "Coronary microcirculation injury"[Title/Abstract] OR "Coronary microcirculation obstruction"[Title/Abstract] OR "Coronary microcirculation lesions"[Title/Abstract] OR "Abnormal coronary microcirculation"[Title/Abstract] OR "Coronary microcirculation dysfunction"[Title/Abstract])
**7**	#5 OR #6
**CMVD associated with INOCA**	#3 AND #4 AND #7
**9**	"percutaneous coronary intervention" [Mesh]
**10**	(Coronary Intervention ， Percutaneous[Title/Abstract]) OR (Coronary Interventions ， Percutaneous[Title/Abstract]) OR (Intervention ， Percutaneous Coronary[Title/Abstract]) OR (Interventions ， Percutaneous Coronary[Title/Abstract]) OR (Percutaneous Coronary Interventions[Title/Abstract]) OR (Percutaneous Coronary Revascularization[Title/Abstract]) OR (Coronary Revascularization ， Percutaneous[Title/Abstract]) OR (Coronary Revascularizations ， Percutaneous[Title/Abstract]) OR (Percutaneous Coronary Revascularizations[Title/Abstract]) OR (Revascularization， Percutaneous Coronary[Title/Abstract]) OR (Revascularizations， Percutaneous Coronary[Title/Abstract]) OR (stable angina pectoris[Title/Abstract]) OR (Unstable angina[Title/Abstract]) OR (acute coronary syndrome[Title/Abstract]) OR (myocardial infarction[Title/Abstract]) OR (Iatrogenic[Title/Abstract]) OR (microcirculation dysfunction[Title/Abstract]) OR (microcirculation lesions[Title/Abstract]) OR (microvascular disorders[Title/Abstract]) OR (Microvascular obstruction[Title/Abstract]) OR (Microcirculation obstruction[Title/Abstract]) OR (Perioperative myocardial injury[Title/Abstract]) OR （ Angina Pectoris with Normal Coronary Arteriogram[Title/Abstract]） OR （IOCA[Title/Abstract]） OR （MIOCA[Title/Abstract]） OR (No

	-Reflow Phenomenon[Title/Abstract]) OR (No Reflow Phenomenon[Title/Abstract]) OR (Slow-Flow Phenomenon[Title/Abstract]) OR (Phenomenon， Slow-Flow[Title/Abstract]) OR (Slow Flow Phenomenon[Title/Abstract])
**11**	#9 OR #10
**CMVD associated with IOCA**	#3 AND #4 AND #11
**12**	"Cardiomyopathy, Dilated"[Mesh]
**13**	(Cardiomyopathies, Dilated[Title/Abstract] OR Dilated Cardiomyopathies[Title/Abstract] OR Dilated Cardiomyopathy[Title/Abstract] OR Cardiomyopathy, Familial Idiopathic[Title/Abstract] OR Cardiomyopathies, Familial Idiopathic[Title/Abstract] OR Familial Idiopathic Cardiomyopathies[Title/Abstract] OR Familial Idiopathic Cardiomyopathy[Title/Abstract] OR Idiopathic Cardiomyopathies, Familial[Title/Abstract] OR Idiopathic Cardiomyopathy, Familial[Title/Abstract] OR Congestive Cardiomyopathy[Title/Abstract] OR Cardiomyopathies,Congestive[Title/Abstract] OR Congestive Cardiomyopathies[Title/Abstract] OR Cardiomyopathy, Congestive[Title/Abstract] OR Cardiomyopathy, Dilated, CMD1A[Title/Abstract] OR Cardiomyopathy, Dilated, LMNA[Title/Abstract] OR Cardiomyopathy, Dilated, Autosomal Recessive[Title/Abstract] OR Cardiomyopathy, Dilated, 1a[Title/Abstract] OR Cardiomyopathy, Idiopathic Dilated[Title/Abstract] OR Cardiomyopathies, Idiopathic Dilated[Title/Abstract] OR Dilated Cardiomyopathies, Idiopathic[Title/Abstract] OR Dilated Cardiomyopathy, Idiopathic[Title/Abstract] OR Idiopathic Dilated Cardiomyopathies[Title/Abstract] OR Idiopathic Dilated Cardiomyopathy[Title/Abstract])
**14**	#12 OR #13
**15**	"Diabetic Cardiomyopathies"[Mesh]
**16**	(Cardiomyopathies, Diabetic[Title/Abstract] OR Cardiomyopathy, Diabetic[Title/Abstract] OR Diabetic Cardiomyopathy[Title/Abstract])
**17**	#15 OR #16
**18**	"Cardiomyopathy, Hypertrophic"[Mesh]
**19**	(Cardiomyopathies, Hypertrophic[Title/Abstract] OR Hypertrophic Cardiomyopathies[Title/Abstract] OR Hypertrophic Cardiomyopathy[Title/Abstract] OR Cardiomyopathy, Hypertrophic Obstructive[Title/Abstract] OR Cardiomyopathies, Hypertrophic Obstructive[Title/Abstract] OR Hypertrophic Obstructive Cardiomyopathies[Title/Abstract] OR Hypertrophic Obstructive Cardiomyopathy[Title/Abstract] OR Obstructive Cardiomyopathies, Hypertrophic[Title/Abstract] OR Obstructive Cardiomyopathy, Hypertrophic[Title/Abstract] OR Cardiovascular neurosis[Title/Abstract] OR Disturbance coronary microcirculation after transplantation[Title/Abstract])
**20**	#18 OR #19
**21**	(CFR[Title/Abstract] OR Coronary Flow Reserve[Title/Abstract] OR IMR[Title/Abstract] OR Coronary microvascular resistance index[Title/Abstract])
**Other types of CMVD**	(#14 OR #17 OR #20) AND #21 AND #3 AND #4

Table S2.2: Search strategy of Web of Science: Science Citation Index Expanded

#	Searches
**1**	TS= (("traditional Chinese medicine" OR "Chinese traditional medicine" OR "zhong yi xue" OR "chung i hsueh") AND TS= (("tongue diagnosis*" OR "tongue assessment*") OR ("Chinese patent drug*" OR capsule OR tablet OR pill OR powder OR "oral liquid" OR granule)))
**2**	(TS= (randomized controlled trial) OR TS= (("controlled clinical trial" OR randomized OR placebo OR "drug therapy" OR randomly OR trial OR groups) NOT TS= ("animal" NOT "human"))) AND DT=(Article)
**3**	TS= (("microvascular angina" OR "cardiac syndrome X" OR "angina syndrome X" OR "coronary microvascular dysfunction" OR "coronary microvascular disease" OR "syndrome X" OR MVA OR CMD OR CMVD OR "coronary microcirculation disease" OR "Angina Pectoris with Normal Coronary Arteriogram" OR INOCA OR "No-Reflow Phenomenon" OR "Slow-Flow Phenomenon" OR "coronary microcirculation dysfunction") NOT TS= (animal NOT human))
**CMVD associated with INOCA**	#1 AND #2 AND #3
4	TS= (("percutaneous coronary intervent*" OR "percutaneous coronary revascularization*" OR PCI) OR ("stable angina pectoris" OR "unstable angina" OR "acute coronary syndrome" OR "myocardial infarction") OR ("coronary microvascular dysfunction" OR "microvascular obstruction" OR "microcirculation obstruction" OR "no-reflow phenomenon" OR "slow-flow phenomenon") OR ("perioperative myocardial injury" OR iatrogenic) OR ("Angina Pectoris with Normal Coronary Arteriogram" OR INOCA OR MINOCA))
**CMVD associated with IOCA**	#1 AND #2 AND #4
**5**	TS= (("dilated cardiomyopathy" OR "congestive cardiomyopathy" OR "idiopathic dilated cardiomyopathy" OR "familial idiopathic cardiomyopathy" OR "CMD1A" OR "LMNA") NOT TS= (animal NOT human))
**6**	TS= ("diabetic cardiomyopathy" OR "diabetic cardiomyopathies") NOT TS= (animal NOT human))
**7**	TS= (("hypertrophic cardiomyopathy" OR "hypertrophic obstructive cardiomyopathy") NOT TS= (animal NOT human))
**8**	#5 OR #6 OR #7
**9**	TS= (CFR OR "Coronary Flow Reserve" OR IMR OR "Coronary microvascular resistance index") NOT TS= (animal NOT human)
**Other types of CMVD**	#8 AND #9 AND #1 AND #2

Table S2.3: Search strategy of Cochrane Central Register of Controlled Trials (CENTRAL)

#	Searches
**1**	MeSH descriptor: [Microvascular Angina] explode all trees
**2**	("syndrome X or "coronary microvascular disease or "coronary microvascular diseases or "coronary microvascular disease "): ti, ab
**3**	#1 or #2
**4**	("Medicine, Chinese Traditional" or "Chinese patent drug" or capsule or tablet or pill or powder or "oral liquid" or granule):ti, ab,kw
**5**	("Randomized Controlled Trial" or "controlled clinical trial" or randomized or placebo or "drug therapy" or randomly or trial or groups): ti, ab
**6**	#3 AND #4 AND #5

Table S2.4: Search strategy of Embase

#	Searches
**1**	'microvascular angina'/exp
**2**	'syndrome X':ab,ti OR 'coronary microvascular disease':ab,ti OR 'coronary microvascular diseases':ab,ti OR 'coronary microvascular diease':ab,ti
**3**	#1 or #2
**4**	'chinese medicine':ab,ti OR 'chinese patent drug':ab,ti OR 'capsule':ab,ti OR 'tablet':ab,ti OR 'pill':ab,ti OR ‘powder':ab,ti OR 'oral liquid':ab,ti OR 'granule':ab,ti
**5**	'randomized controlled trial':ab,ti OR 'controlled clinical trial':ab,ti OR 'randomized':ab,ti OR 'placebo':ab,ti OR 'drug therapy':ab,ti OR 'randomly':ab,ti OR 'trial':ab,ti OR 'groups':ab,ti
**6**	#3 AND #4 AND #5

Table S2.5: Search strategy of CNKI、VIP、and Wanfang

#	Searches
**CMVD associated with INOCA**	(主题:(冠状动脉微血管病) or 主题:(微血管性心绞痛) or 主题:(冠状动脉慢血流) or 主题:(微血管心绞痛) or 主题:(心脏X综合征) or 主题:(冠状动脉微血管疾病) or 主题:(冠状动脉微循环疾病) or 主题:(冠状动脉微循环障碍)) and(主题:(中药) or 主题:(中成药) or 主题:(胶囊) or 主题:(片) or 主题:(丹) or 主题:(散) or 主题:(口服液) or 主题:(丸) or 主题:(颗粒)) and(全部:(随机) or 全部:(安慰剂) or 全部:(盲法) or 全部:(单盲) or 全部:(双盲) or 全部:(三盲) or 全部:(临床研究) or 全部:(临床疗效)) not(主题:(鼠) or 主题:(兔) or 主题:(猪) or 主题:(动物) or 主题:(斑马鱼))
**CMVD associated with IOCA**	(主题:(PCI) or 主题:(经皮冠状动脉介入治疗) or 主题:(经皮冠状动脉介入) or 主题:(经皮冠脉介入) or 主题:(稳定型心绞痛) or 主题:(不稳定性心绞痛) or 主题:(急性冠脉综合征) or 主题:(心肌梗死) or 主题:(医源性) or 主题:(正常冠状动脉造影的胸痛)) and (主题:(微循环障碍) or 主题:(微循环病变) or 主题:(微血管障碍) or 主题:(微血管阻塞) or 主题:(微血管病变) or 主题:(微循环阻塞) or 主题:(无复流) or 主题:(慢血流) or 主题:(围手术期心肌损伤))and(主题:(中药) or 主题:(中成药) or 主题:(胶囊) or 主题:(片) or 主题:(丹) or 主题:(散) or 主题:(口服液) or 主题:(丸) or 主题:(颗粒)) and(全部:(随机) or 全部:(安慰剂) or 全部:(盲法) or 全部:(单盲) or 全部:(双盲) or 全部:(三盲) or 全部:(临床研究) or 全部:(临床疗效)) not(主题:(鼠) or 主题:(兔) or 主题:(猪) or 主题:(动物) or 主题:(斑马鱼))
**Other types of CMVD**	(主题:(糖尿病心肌病) or 主题:(扩张性心肌病) or 主题:(肥厚性心肌病) or 主题:(充血性心肌病) or 主题:(充血型心肌病) or 主题:(肥厚型心肌病) or 主题:扩张型心肌病) or 主题:(应激性心肌病) or 主题:(应激型心肌病) or 主题:(心血管神经症) or 主题:(移植后冠状动脉微循环障碍)) and (主题:(CFR) or 主题:(冠状动脉血流储备) or 主题:(冠脉血流储备) or 主题:(IMR) or 主题:(冠状动脉微血管阻力指数) or 主题:(冠脉微血管阻力指数)) and(主题:(中药) or 主题:(中成药) or 主题:(胶囊) or 主题:(片) or 主题:(丹) or 主题:(散) or 主题:(口服液) or 主题:(丸) or 主题:(颗粒)) and(全部:(随机) or 全部:(安慰剂) or 全部:(盲法) or 全部:(单盲) or 全部:(双盲) or 全部:(三盲) or 全部:(临床研究) or 全部:(临床疗效)) not(主题:(鼠) or 主题:(兔) or 主题:(猪) or 主题:(动物) or 主题:(斑马鱼))

Appendix 3: Characteristics of included studies

Table S3.1: Baseline of characteristics of included studies

Study	Country and region	Design	Follow-up duration	Number of participants	Randomised treatments	Dose and frequency	Allergic rhinitis duration (mean ± SD), years	Age (mean ± SD), years	Outcome indicator
Li Mengjie (2024)	China	RCT	12 weeks	110	Shexiang Baoxin Pill + Aspirin Enteric-coated Tablets + Atorvastatin Calcium Tablets + Metoprolol Succinate SR Tablets 55	Shexiang Baoxin Pill 22.5mg TID po ×12 weeks Aspirin Enteric-coated Tablets 100mg QD po; Atorvastatin Calcium Tablets 20mg QD po; Metoprolol Succinate SR Tablets 47.5mg QD po ×12 weeks	4.11±0.96	59.26±7.62	124589
					Aspirin Enteric-coated Tablets+ Atorvastatin Calcium Tablets+ Metoprolol Succinate SR Tablets 55		3.86±0.87	57.49±8.34	
Yang Yangyang (2023)	China	RCT	12 weeks	40	Shexiang Baoxin Pill + Conventional Treatment 20	Shexiang Baoxin Pill 45mg TID po ×12 weeks	-	-	159
					Conventional Treatment 20	Conventional Treatment× 12 weeks	-	-	
Sun Minli (2022)	China	RCT	4 weeks	111	Shexiang Baoxin Pill + Conventional Treatment 56	Shexiang Baoxin Pill 45mg TID po ×4 weeks	-	51.23±13.85	68
					Conventional Treatment 55	Conventional Treatment×4 weeks	-	52.60±14.76	
Fu Zhaohui (2021)	China	RCT	8 weeks	164	Shexiang Baoxin Pill + Conventional Treatment 82	Shexiang Baoxin Pill 22.5mg TID po ×8 weeks	3.42±1.61	61.48±12.49	467
					Conventional Treatment 82	Conventional Treatment×8 weeks	3.51±2.45	62.13±11.57	
Liu Fajun (2021)	China	RCT	20 weeks	77	Shexiang Baoxin Pill + Diltiazem HCl Tablets 39	Shexiang Baoxin Pill 45mg TID po ×20 weeks	-	56.12±12.75	47
					Diltiazem HCl Tablets 38	Diltiazem HCl Tablets 30mg TID po ×20 weeks	-	55.37±12.89	
Chen Baozeng (2022)	China	RCT	4 weeks	70	Shexiang Baoxin Pill + Conventional Treatment 35	Shexiang Baoxin Pill 45mg TID po ×4 weeks	-	56.0±9.2	68
					Conventional Treatment 35	Conventional Treatment×4 weeks	-	59.0±9.8	
Bai Yuhan (2022).	China	RCT	12 weeks	86	Shexiang Baoxin Pill + Conventional Treatment 39	Shexiang Baoxin Pill 22.5mg TID po ×12 weeks	4.05±1.02	62.71±7.24	14568
					Conventional Treatment 39	Conventional Treatment×12 weeks	3.87±0.96	64.55±6.14	
Chen Xiaolin (2016).	China	RCT	24 weeks	70	Shexiang Baoxin Pill + Aspirin Enteric-coated Tablets + Simvastatin Tablets 37	Shexiang Baoxin Pill 22.5mg TID po ×12 weeks Aspirin Enteric-coated Tablets 100mg QD po; Simvastatin Tablets 20mg QD po ×12 weeks	-	58±13	124
					Aspirin Enteric-coated Tablets+ Simvastatin Tablets 33		-	57±15	
Wu Caiyun (2019).	China	RCT	12 weeks	76	Shexiang Baoxin Pill + Nicorandil Tablets 38	Shexiang Baoxin Pill 22.5mg TID po ×12 weeks	3.5±0.7	53.7±2.6	4678

					Nicorandil Tablets 38		3.4±0.8	54.1±2.5	
Fen Haoli (2019).	China	RCT	8 weeks	60	Shexiang Baoxin Pill + Aspirin Enteric-coated Tablets + Rosuvastatin Tablets 30	Shexiang Baoxin Pill 22.5mg TID po ×8 weeks Aspirin Enteric-coated Tablets 100mg QD po; Rosuvastatin Tablets 5mg QD po ×8 weeks	-	52.13±4.55	3459
					Aspirin Enteric-coated Tablets+ Rosuvastatin Tablets 30		-	52.03±4.23	
Zhang Keqing (2016).	China	RCT	12 weeks	65	Shexiang Baoxin Pill + Aspirin Enteric-coated Tablets + Isosorbide Dinitrate Tablets 35	Shexiang Baoxin Pill 45mg TID po ×12 weeks Aspirin Enteric-coated Tablets 100mg QD po; Isosorbide Dinitrate Tablets 10mg TID po ×12 weeks	-	50.7±6.3	4678
					Aspirin Enteric-coated Tablets+ Isosorbide Dinitrate Tablets 30		-	51.3±7.5	
Yang Guanglong (2017).	China	RCT	4 weeks	45	Shexiang Baoxin Pill + Nicorandil Tablets 22	Shexiang Baoxin Pill 45mg TID po ×4 weeks	-	58.3±3.8	3
					Nicorandil Tablets 23	Nicorandil Tablets 5mg TID po ×4 weeks	-	56.5±3.6	
Wang Shixun (2015).	China	RCT	24 weeks	66	Shexiang Baoxin Pill + Conventional Treatment 34	Shexiang Baoxin Pill 45mg TID po ×24 weeks	-	-	378
					Conventional Treatment 32	Conventional Treatment×24 weeks	-	-	
Xue Zengming (2020).	China	RCT	12 weeks	64	Shexiang Baoxin Pill + Nicorandil Tablets 32	Shexiang Baoxin Pill 45mg TID po ×12 weeks	-	60.98±7.45	58
					Nicorandil Tablets 32	Nicorandil Tablets 5mg TID po ×12 weeks	-	61.02±7.36	
Shen Shuxin (2021).	China	RCT	48 weeks	64	Shexiang Baoxin Pill + Conventional Treatment 32	Shexiang Baoxin Pill 45mg TID po ×48 weeks	-	41.38±9.43	135
					Conventional Treatment 32	Conventional Treatment×48 weeks	-	45.75±10.61	
Zhang Xingzhi (2025).	China	RCT	12 weeks	86	Shexiang Baoxin Pill + Nicorandil Tablets +Aspirin Enteric-coated Tablets + Atorvastatin Calcium Tablets + Metoprolol Succinate SR Tablets + Isosorbide Mononitrate Tablets 43	Shexiang Baoxin Pill 22.5mg TID po ×12 weeks Nicorandil Tablets 5mg TID po; Aspirin Enteric-coated Tablets 100mg QD po; Atorvastatin Calcium Tablets 20mg QD po; Metoprolol Succinate SR Tablets 47.5mg QD po; Isosorbide Mononitrate Tablets 20mg BID po×12 weeks	3.58±1.04	62.28±4.77	4568
					Nicorandil Tablets +Aspirin Enteric-coated Tablets+ Atorvastatin Calcium Tablets+ Metoprolol Succinate SR Tablets+ Isosorbide Mononitrate Tablets 43		3.75±1.12	62.37±4.65	
Sun Kaiyou (2011).	China	RCT	24 weeks	142	Shexiang Baoxin Pill + Conventional Treatment 74	Shexiang Baoxin Pill 45mg TID po ×24 weeks	-	50±7	25
					Conventional Treatment 68	Conventional Treatment×24 weeks	-	51±9	
Zhou Qianglin (2015).	China	RCT	24 weeks	46	Shexiang Baoxin Pill + Conventional Treatment 24	Shexiang Baoxin Pill 45mg TID po ×24 weeks	-	-	3
					Conventional Treatment 22	Conventional Treatment×24 weeks	-	-	
Jin Huilin(2018).	China	RCT	12 weeks	100	Shexiang Baoxin Pill + Nicorandil Tablets 50	Shexiang Baoxin Pill 45mg TID po ×12 weeks	-	54.5±6.4	4678
					Nicorandil Tablets 50	Nicorandil Tablets 5mg TID po ×12 weeks	-	53.6±6.5	

Cai Xiaodun (2017).	China	RCT	12 weeks	60	Shexiang Baoxin Pill + Nicorandil Tablets 30	Shexiang Baoxin Pill 45mg TID po ×12 weeks	-	52.5±6.6	4678
					Nicorandil Tablets 30	Nicorandil Tablets 5mg TID po ×12 weeks	-	52.6±6.5	
Zhen Wenhui (2024).	China	RCT	12 weeks	100	Shexiang Tongxin Dripping Pills + Conventional Treatment 52	Shexiang Tongxin Dripping Pills 70 mg TID po ×12 weeks	6.4±1.3	56.8±7.5	359
					Conventional Treatment 48	Conventional Treatment×12 weeks	6.8±1.8	57.1±7.1	
Qin Xiaofei (2023).	China	RCT	24 weeks	100	Shexiang Tongxin Dripping Pills + Conventional Treatment 55	Shexiang Tongxin Dripping Pills 70 mg TID po ×24 weeks	-	55.78±8.55	29
					Conventional Treatment 56	Conventional Treatment×24 weeks	-	57.00±10.00	
Gong Yumiao (2021).	China	RCT	13 weeks	106	Shexiang Tongxin Dripping Pills + Nicorandil Tablets +Aspirin Enteric-coated Tablets + Atorvastatin Calcium Tablets + Metoprolol Succinate SR Tablets + Isosorbide Mononitrate Tablets 54	Shexiang Tongxin Dripping Pills 70 mg TID po ×13 weeks Nicorandil Tablets 5mg TID po; Aspirin Enteric-coated Tablets 100mg QD po; Atorvastatin Calcium Tablets 20mg QD po; c 47.5mg QD po; Isosorbide Mononitrate Tablets 20mg BID po×13 weeks	-	62.71±5.32	4678
					Nicorandil Tablets +Aspirin Enteric-coated Tablets+ Atorvastatin Calcium Tablets+ Metoprolol Succinate SR Tablets+ Isosorbide Mononitrate Tablets 52		-	61.98±5.39	
Liu Yong (2025).	China	RCT	12 weeks	64	Shexiang Tongxin Dripping Pills + Conventional Treatment 32	Shexiang Tongxin Dripping Pills 70 mg TID po ×12 weeks	-	59.94±9.63	10
					Conventional Treatment 32	Conventional Treatment×12 weeks	-	60.78±11.13	
Han Yong (2024).	China	RCT	4 weeks	88	Shexiang Tongxin Dripping Pills + Conventional Treatment 44	Shexiang Tongxin Dripping Pills 70 mg TID po ×4 weeks	-	63.23±6.33	2379
					Conventional Treatment 44	Conventional Treatment×4 weeks	-	63.14±6.35	
Li Kangrong (2021).	China	RCT	24 weeks	72	Shexiang Tongxin Dripping Pills + Conventional Treatment 36	Shexiang Tongxin Dripping Pills 70 mg TID po ×24 weeks	-	61.23±6.37	10
					Conventional Treatment 36	Conventional Treatment×24 weeks	-	60.92±6.14	
He Na (2021).	China	RCT	48 weeks	128	Tongxinluo Capsules + Conventional Treatment 65	Tongxinluo Capsules 4 capsules TID po ×48 weeks	-	59.28± 15.92	57
					Conventional Treatment 63	Conventional Treatment×48 weeks	-	60.58± 16.97	
Zhang Chunhui (2020).	China	RCT	12 weeks	100	Tongxinluo Capsules + Nicorandil Tablets +Aspirin Enteric-coated Tablets+ Atorvastatin Calcium Tablets 50	Tongxinluo Capsules 2 capsules TID po ×12 weeks Nicorandil Tablets 5mg TID po; Aspirin Enteric-coated Tablets 100mg QD po; Atorvastatin Calcium Tablets 20mg QD po×12weeks	3.15±0.62	55.21±2.27	347
					Nicorandil Tablets +Aspirin Enteric-coated Tablets+ Atorvastatin Calcium Tablets 50		3.12±0.68	55.80±2.21	
Chen Hao (2022).	China	RCT	24 weeks	40	Tongxinluo Capsules + Conventional Treatment 20	Tongxinluo Capsules 4 capsules TID po ×24 weeks	-	52.25±10.14	347
					Conventional Treatment 20	Conventional Treatment×24 weeks	-	53.14±9.10	
Qin Gang (2017).	China	RCT	24 weeks	115	Tongxinluo Capsules + Nicorandil Tablets + Simvastatin Tablets 58	Tongxinluo Capsules 2 capsules TID po ×24 weeks	-	60.6±5.2	37
					Nicorandil Tablets + Simvastatin Tablets 57	Nicorandil Tablets 5mg TID po; Simvastatin Tablets 20mg QD po×24weeks	-	59.3±6.0	

Ge Hailong (2015).	China	RCT	12 weeks	71	Tongxinluo Capsules + Conventional Treatment 38	Tongxinluo Capsules 3 capsules TID po ×12 weeks	-	56±8	2
					Conventional Treatment 33	Conventional Treatment×12 weeks	-	54±7	
Wang Zhiqian (2016).	China	RCT	12 weeks	112	Tongqiao Biyan Tablets + Mometasone Furoate Nasal Spray 56	Tongxinluo Capsules 3 capsules TID po ×12 weeks	-	52.25±10.14	468
					Mometasone Furoate Nasal Spray 56	Conventional Treatment×12 weeks	-	53.14±9.10	
Sun Xiaofang (2024).	China	RCT	24 weeks	80	Tongxinluo Capsules + Nicorandil Tablets +Aspirin Enteric-coated Tablets+ Atorvastatin Calcium Tablets 40	Tongxinluo Capsules 2 capsules TID po ×24 weeks Nicorandil Tablets 5mg TID po; Aspirin Enteric-coated Tablets 100mg QD po; Atorvastatin Calcium Tablets 20mg QD po×24weeks	-	60.25±2.78	37
					Nicorandil Tablets +Aspirin Enteric-coated Tablets+ Atorvastatin Calcium Tablets 40		-	61.45±2.51	
Wei Wei (2022).	China	RCT	24 weeks	106	Tongxinluo Capsules + Nicorandil Tablets +Aspirin Enteric-coated Tablets+ Atorvastatin Calcium Tablets 53	Tongxinluo Capsules 2 capsules TID po ×24 weeks Nicorandil Tablets 5mg TID po; Aspirin Enteric-coated Tablets 100mg QD po; Atorvastatin Calcium Tablets 20mg QD po×24weeks	-	70.1±5.9	3678
					Nicorandil Tablets +Aspirin Enteric-coated Tablets+ Atorvastatin Calcium Tablets 53		-	68.9±6.0	
Peng Qinqkui (2014).	China	RCT	24 weeks	42	Tongxinluo Capsules + Nicorandil Tablets +Aspirin Enteric-coated Tablets+ Atorvastatin Calcium Tablets 21	Tongxinluo Capsules 2 capsules TID po ×24 weeks Nicorandil Tablets 5mg TID po; Aspirin Enteric-coated Tablets 100mg QD po; Atorvastatin Calcium Tablets 20mg QD po×24 weeks	-	-	37
					Nicorandil Tablets +Aspirin Enteric-coated Tablets+ Atorvastatin Calcium Tablets 21		-	-	
Chen Baozeng (2019).	China	RCT	24 weeks	120	Xinkeshu Tablets + Aspirin Enteric-coated Tablets+Metoprolol Tablets+Rosuvastatin Calcium Tablets+ Isosorbide Mononitrate Tablets 60	Xinkeshu Tablets 1.24g TID po ×24 weeks Aspirin Enteric-coated Tablets 100mg QD po; Metoprolol Tablets 23.75mg QD po; Rosuvastatin Calcium Tablets 10mg QD po; Isosorbide Mononitrate Tablets 5mg TID po×24 weeks	-	66.1±4.6	468
					Aspirin Enteric-coated Tablets+Metoprolol Tablets+Rosuvastatin Calcium Tablets+ Isosorbide Mononitrate Tablets 60		-	66.7±3.7	
Li Yuan (2017).	China	RCT	24 weeks	110	Xinkeshu Tablets + Conventional Treatment 55	Xinkeshu Tablets (0.31g/tablet) 1.24g TID po×24 weeks	-	58.20±10.26	4568
					Conventional Treatment 55	Conventional Treatment×24 weeks	-	57.10±9.52	
Jia Wanming (2017).	China	RCT	12 weeks	60	Xinkeshu Tablets + Nicorandil Tablets+Conventional Treatment 30	Xinkeshu Tablets (0.31g/tablet) 1.24g TID po;Nicorandil Tablets 5mg TID po ×12 weeks Conventional Treatment×12 weeks	-	48.93±5.06	467
					Conventional Treatment 30		-	47.77±5.86	

Liang Yuefeng (2019).	China	RCT	24 weeks	77	Xinkeshu Tablets + Bisoprolol Fumarate Tablets+Aspirin Enteric-coated Tablets+Trimetazidine Dihydrochloride Tablets+Atorvastatin Calcium Tablets 38	Xinkeshu Tablets 4 tablets TID po×24weeks Bisoprolol Fumarate Tablets 2.5mg QD po; Aspirin Enteric-coated Tablets 100mg QD po; Trimetazidine Dihydrochloride Tablets 20mg TID po; Atorvastatin Calcium Tablets 10mg QD po×24 weeks	4.7±1.5	69.97±8.48	7
					Bisoprolol Fumarate Tablets+Aspirin Enteric-coated Tablets+Trimetazidine Dihydrochloride Tablets+Atorvastatin Calcium Tablets 39		4.3±1.2	70.46±7.75	
Lai Haiqing (2025).	China	RCT	4 weeks	84	Xinkeshu Tablets + Nicorandil Tablets+Conventional Treatment 42	Xinkeshu Tablets 4 tablets TID po×4weeks Conventional Treatment×4weeks	3.09±0.55	65.27±5.87	12459
					Conventional Treatment 42		3.12±0.58	65.35±5.92	
Zhao Danhua (2024).	China	RCT	24 weeks	200	Xinbao Pills + Conventional Treatment 100	Xinbao Pills 2 pills TID po ×24weeks Conventional Treatment ×24weeks	8.52±1.19	59.74±9.63	246789
					Conventional Treatment 100		8.45±1.26	60.14±9.14	
Zhao Danhua (2021).	China	RCT	12 weeks	122	Xinbao Pills + Losartan Potassium Tablets+Conventional Treatment 61	Xinbao Pills 2 pills TID po ×12weeks Losartan Potassium Tablets 50mg QD po; Conventional Treatment ×12weeks	1.07±0.39	58.31±7.34	12
					Losartan Potassium Tablets+Conventional Treatment 61		1.17±0.35	60.03±6.97	
Zhao Danhua (2023).	China	RCT	4 weeks	198	Xinbao Pills + Diltiazem Hydrochloride Tablets+Conventional Treatment 99	Xinbao Pills 2 pills TID po ×4weeks Diltiazem Hydrochloride Tablets 30mg TID po×4weeks	-	56.79±4.15	489
					Diltiazem Hydrochloride Tablets+Conventional Treatment 99		-	56.96±5.24	
Zhang Yuanhen (2020).	China	RCT	12 weeks	124	Danshen Dripping Pills +Conventional Treatment 62	Danshen Dripping Pills 10 pills TID po ×12weeks Conventional Treatment ×12weeks	7.04±2.98	62.05±9.87	3
					Conventional Treatment 62		7.53±2.77	61.72±10.15	
Wei Ying (2018).	China	RCT	12 weeks	80	Danshen Dripping Pills + Aspirin Enteric-coated Tablets+Metoprolol Tablets+ Isosorbide Mononitrate Tablets 40	Danshen Dripping Pills 10 pills TID po ×12weeks Aspirin Enteric-coated Tablets 100mg QD po; Metoprolol Tablets 25mg BID po; Isosorbide Mononitrate Tablets 20mg BID po×12 weeks	-	58.28±11.25	479
					Aspirin Enteric-coated Tablets+Metoprolol Tablets+ Isosorbide Mononitrate Tablets 40		-	56.48±10.75	
Li Na (2025).	China	RCT	4 weeks	104	Danshen Dripping Pills + Verapamil Hydrochloride Tablets 52	Danshen Dripping Pills 10 pills TID po ×4weeks Verapamil Hydrochloride Tablets 80mg TID po ×4weeks	7.38±2.01	45.48±6.35	24689
					Verapamil Hydrochloride Tablets 52		7.27±1.91	45.78±6.46	
Wang Zhen (2019).	China	RCT	24 weeks	87	Yindan Xinnao Tong Capsules +Conventional Treatment 43	Yindan Xinnao Tong Capsules 0.8g TID po×24weeks Conventional Treatment×24weeks	-	57.3±11.9	1678
					Conventional Treatment 44		-	56.1±13.2	
Wang Caige (2022).	China	RCT	24 weeks	130	Yindan Xinnao Tong Capsules + Nicorandil Tablets 65	Yindan Xinnao Tong Capsules 0.8g TID po×24weeks Nicorandil Tablets 5mg TID po ×24 weeks	2.09±1.72	57.82±4.79	4689
					Nicorandil Tablets 65		2.16±1.64	58.17±3.36	

Peng Chaan (2024).	China	RCT	4 weeks	104	Yindan Xinnao Tong Capsules + Nicorandil Tablets 63	Yindan Xinnao Tong Capsules 0.8g TID po×4weeks	6.49±2.61	59.78±14.72	4789
					Nicorandil Tablets 63	Nicorandil Tablets 5mg TID po ×4 weeks	6.14±2.24	59.34±14.26	
Kang Lirui (2021)	China	RCT	12 weeks	60	Qishen Dripping Pills+Conventional Treatment 30	Qishen Dripping Pills 0.5g TID po ×12weeks	-	57.2±11.46	249
					Conventional Treatment 30	Conventional Treatment×12weeks	-	60.00±13.04	
Sun Jinhua (2024)	China	RCT	48 weeks	76	Qishen Dripping Pills + Nicorandil Tablets 38	Qishen Dripping Pills 0.5g TID po ×48weeks	-	53.34±10.02	35
					Nicorandil Tablets 38	Nicorandil Tablets 5mg TID po ×248weeks	-	53.37±10.0	
Shen Xia (2021)	China	RCT	8 weeks	68	Qishen Dripping Pills+Conventional Treatment 34	Qishen Dripping Pills 0.5g TID po ×8weeks	0.97±0.08	65.41±4.27	8
					Conventional Treatment 34	Conventional Treatment×8weeks	0.96±0.08	65.43±4.29	
Zhang Yinli (2013)	China	RCT	12 weeks	120	Qishen Dripping Pills +Conventional Treatment 60	Qishen Dripping Pills 0.5g TID po ×12weeks	1.25±0.39	55.7±3.2	12
					Trimetazidine Dihydrochloride Tablets 60	Trimetazidine Dihydrochloride Tablets 20mg TID po×12weeks	1.50±0.29	58.6±4.4	
Yan Weili (2018)	China	RCT	12 weeks	118	Qishen Dripping Pills + Nicorandil Tablets 59	Qishen Dripping Pills 0.5g TID po ×12weeks	-	60.37±5.12	4678
					Nicorandil Tablets 59	Nicorandil Tablets 5mg TID po ×12weeks	-	61.16±5.47	
Lai Peiwen (2024)	China	RCT	4 weeks	104	Kuanxiong Aerosol+Nicorandil Tablets 52	Kuanxiong Aerosol (5.8 g: 60 sprays)3 sprays TID×4weeks	-	54.23±5.06	39
					Nicorandil Tablets 52	Nicorandil Tablets 5mg TID po ×4weeks	-	54.68±5.32	
Liu Xiqi （2024）	China	RCT	4 weeks	60	Kuanxiong Aerosol+Conventional Treatment 30	Kuanxiong Aerosol (5.8 g: 60 sprays)3 sprays TID×4weeks	-	66.37±12.94	7
					Conventional Treatment 30	Conventional Treatment×4weeks	-	66.82±9.36	
Liu Rui (2025)	China	RCT	6 weeks	81	Xueshuantong Capsules+ Diltiazem Hydrochloride Tablets+Conventional Treatment 41	Xueshuantong Capsules 3 pills TID po ×6weeks	4.88±1.16	61.03±8.22	24689
					Diltiazem Hydrochloride Tablets+Conventional Treatment 40	Diltiazem Hydrochloride Tablets 30mg TID po×6weeks	5.11±1.27	60.15±8.05	
Wang Bo (2024)	China	RCT	12 weeks	87	Xueshuantong Capsules+Conventional Treatment 43	Xueshuantong Capsules 3 pills TID po×12weeks		60.1±10.7	4789
					Conventional Treatment 44	Conventional Treatment×12weeks		61.0±8.5	
Kong Xiao (2025)	China	RCT	12 weeks	92	Yangxin Shengmai Granules+Conventional Treatment 43	Yangxin Shengmai Granules 14g TID po×12weeks	-	68.3±7.2	4589
					Conventional Treatment 49	Conventional Treatment×12weeks	-	69.1±6.9	
Zhou Xiaokai (2024)	China	RCT	8 weeks	118	Shenxiang Suhe Pills+Conventional Treatment 59	Shenxiang Suhe Pills 0.7g BID po×8weeks	-	67.05±8.15	249
					Conventional Treatment 59	Conventional Treatment×8weeks	-	66.83±7.53	
Wang Haiyang	China	RCT	4 weeks	76	Lingbao Huxin Dan+Conventional Treatment 38	Lingbao Huxin Dan 4 pills BID po×4weeks	-	62.70±5.63	2

					Conventional Medicine Treatment 38		-	61.90±6.75	
Zhang Hongbin (2021)	China	RCT	24 weeks	80	Qili Qiangxin Capsules+Nicorandil Tablets 40	Qili Qiangxin Capsules 4 capsules TID po×24weeks	-	58.3±7.0	3678
					Nicorandil Tablets 40	Nicorandil Tablets 5mg TID po ×24weeks	-	57.6±6.5	
Li Huiying (2019)	China	RCT	8 weeks	102	Qili Qiangxin Capsules+Nicorandil Tablets+Conventional Treatment 51	Liqi Huoxue Dripping Pills 10 pills TID po×8 weeks	-	52.51±8.42	678
					Nicorandil Tablets+Conventional Treatment 51	Nicorandil Tablets 5mg TID po ×8weeks	-	54.29±8.42	
Liu Yan (2017)	China	RCT	24 weeks	120	Dengzhan Shengmai Capsules+Conventional Treatment 60	Dengzhan Shengmai Capsules 3 capsules TID po×24weeks	-	58.20±10.26	678
					Conventional Treatment 60	Conventional Treatment×24weeks	-	57.10±9.52	
Wu Youhua (2018)	China	RCT	4 weeks	82	Yuxintong Capsules+Nicorandil Tablets+Conventional Treatment 41	Yuxintong Capsules 4 capsules TID po ×4weeks	5.48±0.52	53.35±1.43	468
					Nicorandil Tablets+Conventional Treatment 41	Nicorandil Tablets 5mg TID po ×4weeks	5.27±0.34	53.13±1.26	
Li Chao (2025)	China	RCT	8 weeks	84	Yixinshu Tablets+Rosuvastatin Calcium Tablets+Conventional Treatment 42	Yixinshu Tablets 1.2g TID po× 8weeks	4.74±1.10	64.61±3.06	124568
					Rosuvastatin Calcium Tablets+Conventional Treatment 42	Rosuvastatin Calcium Tablets 10mg QD po×8weeks	5.03±1.04	63.25±3.54	
Chen Huashan (2024)	China	RCT	10 weeks	60	Guanxinning Tablets+Conventional Treatment 30	Guanxinning Tablets 1.52g TID po× 10weeks	-	57.5±6.5	346
					Conventional Treatment 30	Conventional Treatment×10weeks	-	58.8±6.2	

Abbreviations: PO, oral intake; QD, once daily; BID, twice daily; TID, three times daily; y, years old; m, month; w, week; d, day; Outcome indicator : 1, Index of Microcirculatory Resistance(IMR); 2, Coronary Flow Reserve(CFR); 3, Corrected Thrombolysis in Myocardial Infarction Frame Count(cTFC); 4, Total Effective Rate; 5, Left Ventricular Ejection Fraction(LVEF); 6, Nitric Oxide(NO); 7, High-Sensitivity C-Reactive Protein(hs-CRP); 8, Endothelin-1(ET-1); 9, Adverse event.

Table S3.2: Manufacturer Details and Regulatory Information of Included Medicines

CCPPs	Manufacturer	Specification	Approval Number	Production Batch Number
Shexiang Baoxin Pill	Shanghai Hutchison Pharmaceuticals Co., Ltd.	22.5mg/pill	Z31020068	-
Shexiang Tongxin Dripping Pills	Inner Mongolia Conba Pharmaceutical Co., Ltd. Shenglong Branch	35 mg /pill	Z20080018	-
Tongxinluo Capsules	Shijiazhuang Yiling Pharmaceutical Co., Ltd.	0.26 g/capsule	Z19980015	1410010
Xinkeshu Tablets	Shandong Wohua Pharmaceutical Science & Technology Co., Ltd.	0.31 g/tablet	Z37020042	-
Xinbao Pills	Guangdong Xinbao Pharmaceutical Science & Technology Co., Ltd.	60 mg/pill	Z44021843	-
Danshen Dripping Pills	Tasly Pharmaceutical Group Co., Ltd.	27 mg/pill	Z10950111	161201
Yindan Xinnao Tong Capsules	Guizhou Bailing Enterprise Group Pharmaceutical Co., Ltd.	0.4 g/capsule	Z20027144	20130330 20140610 20160120
Qishen Dripping Pills	Tasly Pharmaceutical Group Co., Ltd.	0.5 g/bag	Z20030139	171202
Kuanxiong Aerosol	Zhejiang Sukoan Pharmaceutical Co., Ltd.	5.8 g/60 actuations	Z20163023	-
Xueshuantong Capsules	Guangdong Zhongsheng Pharmaceutical Co., Ltd.	0.5 g/capsule	Z20030017	220230 230419
Yangxin Shengmai Granules	Qinhuangdao Shanguan Pharmaceutical Co., Ltd.	14 g/bag	Z20030096	-
Shenxiang Suhe Pills	Hangzhou Hu Qing Yu Tang Pharmaceutical Co., Ltd.	0.7 g/bottle	Z33020141	22063
Lingbao Huxin Dan	Leiyunshang Pharmaceutical Group Co., Ltd.	0.08 g/pill	Z32021181	05002
Qili Qiangxin Capsules	Shijiazhuang Yiling Pharmaceutical Co., Ltd.	0.3 g/capsule	Z20040141	-
Liqi Huoxue Dripping Pills	Guizhou Minzu Pharmaceutical Co., Ltd.	25 mg/pill	Z20120037	-
Dengzhan Shengmai Capsules	Yunnan Shengwugu Pharmaceutical Co., Ltd.	0.18 g/capsule	Z20026439	-
Yuxintong Capsules	Jilin Aodong Group Dalian Pharmaceutical Co., Ltd.	0.33 g/capsule	Z20020089	160109 170108
Yixinshu Tablets	Guizhou Xinbang Pharmaceutical Co., Ltd.	0.6 g/tablet	Z20090491	-
Guanxinning Tablets	Zhengda Qingchunbao Pharmaceutical Co., Ltd.	0.38 g/tablet	Z20150028	-

Medicine	Manufacturer	Specification	Approval Number	Production Batch Number
Nicorandil Tablets	Jinzhou Jiutai Pharmaceuticals Co., Ltd.	5 mg/tablet	H21022583	-
	Chugai Pharmaceutical Co., Ltd.	5 mg/tablet	H20110492	-
	Guangxi Nanning Baihui Pharmaceuticals Group Co., Ltd.	5 mg/tablet	H45021071	-
Aspirin Enteric-coated Tablets	Bayer HealthCare Manufacturing S.r.l., Italy	100 mg/tablet	HJ20160685	-
Atorvastatin Calcium Tablets	Pfizer Pharmaceuticals Limited	20 mg/tablet	H20051408	-
Metoprolol Succinate SR Tablets	AstraZeneca AB	47.5mg/tablet	HJ20150044	-
	AstraZeneca Pharmaceuticals Co., Ltd.	47.5mg/tablet	H32025391	-
Isosorbide Mononitrate Tablets	Ruiyang Pharmaceuticals Joint Stock Co., Ltd.	20 mg/tablet	H20213309	-
	Qilu Pharmaceuticals Co., Ltd.	20 mg/tablet	H20065685	-
Rosuvastatin Tablets	Shengrui Pharmaceuticals Group	10 mg/tablet	H20203475	-
Simvastatin Tablets	Jiangsu Huanghe Pharmaceuticals Joint Stock Co., Ltd.	20 mg/tablet	H20067793	-
Diltiazem HCl Tablets	Jiangsu Hengrui Pharmaceuticals Joint Stock Co., Ltd.	30 mg/tablet	H20200916	-
Bisoprolol Fumarate Tablets	Merck KGaA	2.5mg/tablet	HJ20160475	X19990258
Trimetazidine Dihydrochloride Tablets	Beijing Wansheng Pharmaceutical Co., Ltd.	20mg/tablet	H20065167	090801
	Servier Pharmaceuticals Co., Ltd.	20mg/tablet	H20055465	-
Verapamil Hydrochloride Tablets	Guangzhou Baiyunshan Pharmaceutical Holdings Co., Ltd.	40mg/tablet	H44021499	-
Losartan Potassium Tablets	Sandoz GmbH	50mg/tablet	H20140913	-

Appendix 4: List of data extracted from the included randomized clinical trials

Data category	List of variables
Study	Primary author, year of publication, duration of study, total number of patients in each group
Patients	Age, nationality, duration
Interventions	Drug class, dose, and duration of the primary intervention, and strategies used for implementing them
Efficacy outcome	Mean of change in IMR (mmHg·s/mL), CFR, cTFC (frames), LVEF (%), NO (μmol/L), hs-CRP (mg/L), ET-1 (ng/L), with respective standard deviation from baseline.
Adverse events	Various adverse events reported in the included trials

Abbreviations: IMR, index of microcirculatory resistance; CFR, coronary flow reserve; cTFC, corrected thrombolysis in myocardial infarction frame count; LVEF, left ventricular ejection fraction; hs-CRP, high-sensitivity C-reactive protein; NO, nitric oxide; ET-1, endothelin-1

.

Appendix 5: Risk of bias of randomized clinical trials

Table S5: Study-level risk of bias assessment using Cochrane risk of bias tool 2.0 for assessing risk of bias of randomized clinical trials.

Unique ID	Randomization process	Deviations from intended interventions	Mising outcome data	Measurement of the outcome	Selection of the reported result	Over all
Li Mengjie (2024)	low	some concerns	low	low	low	some concerns
Yang Yangyang (2023)	low	some concerns	low	low	low	some concerns
Sun Minli (2022)	low	some concerns	low	low	low	some concerns
Fu Zhaohui (2021)	low	some concerns	low	low	low	some concerns
Liu Fajun (2021)	low	some concerns	low	low	low	some concerns
Chen Baozeng (2022)	low	some concerns	low	low	low	some concerns
Bai Yuhan (2022)	low	some concerns	low	low	low	some concerns
Chen Xiaolin (2016)	low	some concerns	low	low	low	some concerns
Wu Caiyun (2019)	low	some concerns	low	low	low	some concerns
Fen Haoli (2019)	low	some concerns	low	low	low	some concerns
Zhang Keqin (2016)	low	some concerns	low	low	low	some concerns
Yang Guanglong (2017)	low	some concerns	low	low	low	some concerns
Wang Shixun (2015)	low	some concerns	low	low	low	some concerns
Xue Zengmin (2020)	low	some concerns	low	low	low	some concerns
Shen Shuxin (2021)	low	some concerns	low	low	low	some concerns
Zhang Xinzhi (2025)	low	some concerns	low	low	low	some concerns
Sun Kaiyou (2011)	some concerns	some concerns	low	low	some concerns	high
Zhou Qianglin (2015)	low	some concerns	low	low	low	some concerns
Jin Huilin (2018)	low	some concerns	low	low	low	some concerns
Cai Xiaodun (2017)	low	some concerns	low	low	low	some concerns
Zhen Wenhui (2024)	low	some concerns	low	low	low	some concerns
Qin Xiaofei (2023)	low	some concerns	low	low	low	some concerns
Gong Yumiao (2021)	low	some concerns	low	low	low	some concerns
Liu Yong (2025)	low	some concerns	some concerns	low	some concerns	high
Han Yong (2024)	low	some concerns	low	low	low	some concerns
Li Kangrong (2021)	low	some concerns	low	low	low	some concerns
He Na (2021)	low	some concerns	low	low	low	some concerns
Zhang Chunhui (2020)	low	some concerns	low	low	low	some concerns
Chen Hao (2022)	low	some concerns	low	low	low	some concerns
Qin Gang (2017)	low	some concerns	low	low	low	some concerns
Ge Hailong	low	some concerns	low	low	low	some concerns

(2015)						
Wang Zhiqian (2014)	low	some concerns	low	low	low	some concerns
Sun Xiaofang (2024)	low	some concerns	low	low	low	some concerns
Wei Wei (2022)	low	some concerns	low	low	low	some concerns
Peng Qinkui (2014)	some concerns	some concerns	low	low	some concerns	high
Chen Baozeng (2019)	low	some concerns	low	low	low	some concerns
Li Yuan (2017)	low	some concerns	low	low	low	some concerns
Jia Wanming (2019)	low	some concerns	low	low	low	some concerns
Liang Yuefeng (2019)	low	some concerns	low	low	low	some concerns
Lai Haiqin (2025)	low	some concerns	low	low	low	some concerns
Zhao Danhua (2024)	low	some concerns	low	low	low	some concerns
Zhao Danhua (2021)	low	some concerns	low	low	low	some concerns
Zhao Danhua (2023)	low	some concerns	low	low	low	some concerns
Wang Zhen (2019)	low	some concerns	low	low	low	some concerns
Wang Caige (2022)	low	some concerns	low	low	low	some concerns
Kang Lirui (2021)	low	some concerns	low	low	low	some concerns
Sun Jinhua (2024)	low	some concerns	low	low	low	some concerns
Shen Xia (2021)	low	some concerns	low	low	low	some concerns
Zhang Yinli (2013)	low	some concerns	low	low	low	some concerns
Yan Weili (2018)	low	some concerns	low	low	low	some concerns
Lai Peiwen (2024)	low	some concerns	low	low	low	some concerns
Liu Xiqi (2024)	low	some concerns	low	low	low	some concerns
Zhang Yuanhen (2020)	low	some concerns	low	low	low	some concerns
Wei Ying (2018)	low	low	low	low	low	low
Liu Rui (2025)	high	some concerns	low	low	low	high
Li Na (2025)	low	some concerns	low	low	low	some concerns
Kong Xiao (2025)	high	some concerns	low	low	low	high
Zhou Xiaokai (2024)	low	some concerns	low	low	low	some concerns
Wang Haiyang (2024)	low	some concerns	low	low	low	some concerns
Wang Bo (2024)	low	some concerns	low	low	low	some concerns
Peng Chaan (2024)	low	some concerns	low	low	low	some concerns
Zhang Hongbin (2021)	low	some concerns	low	low	low	some concerns
Li Huiyin (2019)	low	some concerns	low	low	low	some concerns
Liu Yan (2017)	low	some concerns	low	low	low	some concerns
Wu Youhua (2018)	high	some concerns	low	low	some concerns	high
Li Chao (2025)	high	some concerns	low	low	some concerns	high
Chen Huashan (2024)	high	some concerns	low	low	some concerns	high

Appendix 6: Evaluation of inconsistency and heterogeneity

No closed treatment loops existed in the current evidence network, so loop-specific local inconsistency testing was not feasible. Global inconsistency was assessed using the design-by-treatment interaction model in Stata MP 18.0. Global inconsistency χ^2 and P-values for each outcome were summarized in Table S6. No significant global inconsistency was detected across all outcomes. Between-study heterogeneity was quantified by τ^2.

Table S6: Global consistency and Heterogeneity score

Clinical outcome	Chi square	P value	τ2 network
IMR	1.61	0.2041	0.96
CFR	5.31	0.2131	0.19
cTFC	24.66	0.4981	0.71
LVEF	2.64	0.4412	0.88
NO	4.44	0.3521	0.51
hs-CRP	29.92	0.0552	0.57
ET-1	15.12	0.6217	0.95
Effective rate	10.54	0.0665	0.04
Adverse events	4.75	0.0593	0.05

Abbreviations: IMR, index of microcirculatory resistance; CFR, coronary flow reserve; cTFC, corrected thrombolysis in myocardial infarction frame count; LVEF, left ventricular ejection fraction; hs-CRP, high-sensitivity C-reactive protein; NO, nitric oxide; ET-1, endothelin-1.

Appendix 7: Network maps and forest plots of secondary outcomes

Figure S7.1: Network map of the total effective rate, and forest plot of network effect sizes compared with Conventional Medicine treatment. The size of the nodes was proportional to the number of participants included in the trial, and the thickness of lines between the interventions relates to the number of studies for that comparison.

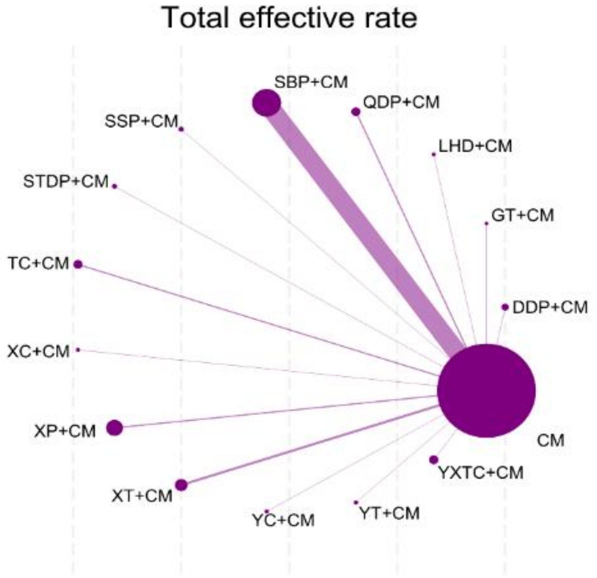

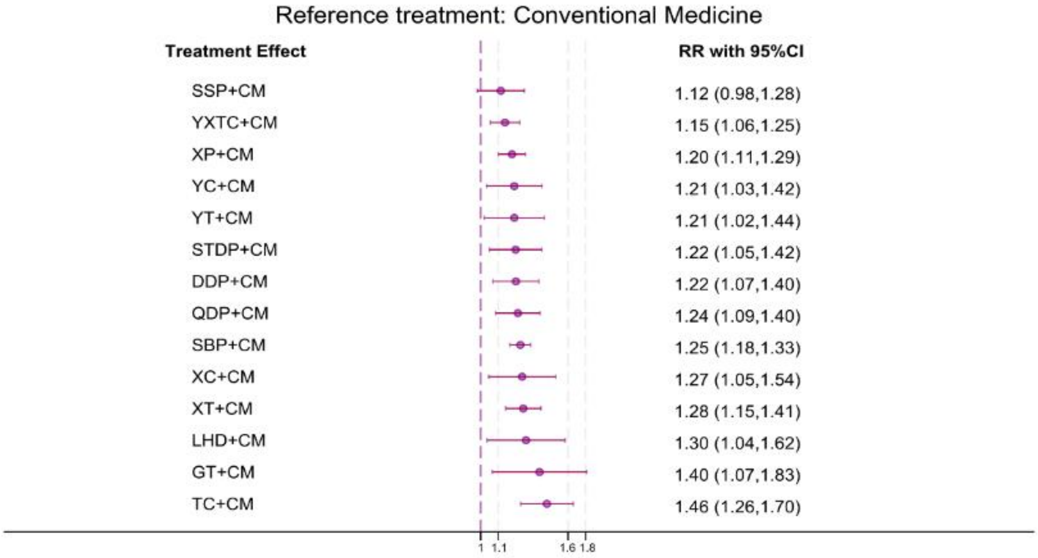

Abbreviations: SUCRA, surface under the cumulative ranking curve; CM, Conventional Medicine; TC, Tongxinluo Capsules; GT, Guanxinning Tablets; LHD,Lingbao Huxin Dan; XT, Xinkeshu Tablets; SBP, Shexiang Baoxin Pills; XC, Xinxuekang Capsules; QDP,Qishen Dripping Pills; DDP,Danshen Dripping Pills; STDP,Shexiang Tongxin Dripping Pills; YC,Yuxintong Capsules; YT, Yixinshu Tablets; XP,Xinbao Pills; YXTC,Yindan Xinnao Tong Capsules; SSP, Shenxiang Suhe Pills.

Figure S7.2: Network map of the effect on LVEF, and forest plot of network effect sizes compared with Conventional Medicine treatment. The size of the nodes was proportional to the number of participants included in the trial, and the thickness of lines between the interventions relates to the number of studies for that comparison.

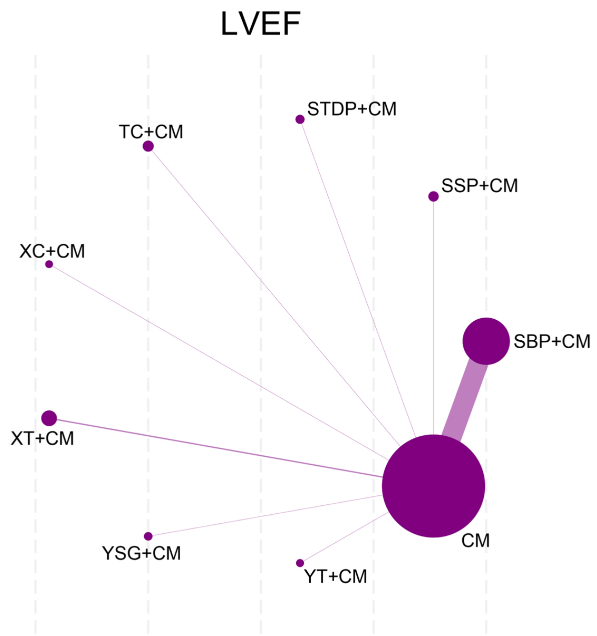

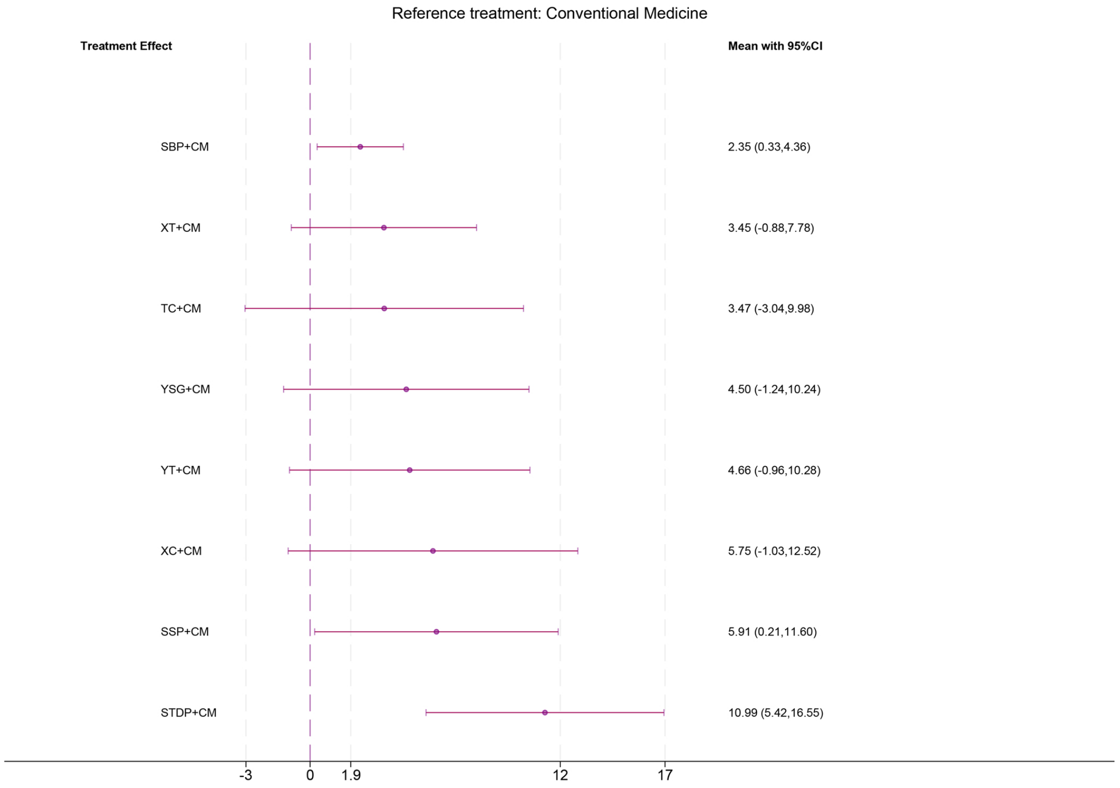

Abbreviations: CM, Conventional Medicine; SBP, Shexiang Baoxin Pills; SSP, Shenxiang Suhe Pills; STDP, Shexiang Tongxin Dripping Pills; TC, Tongxinluo Capsules; XC, Xinxuekang Capsules; XT, Xinkeshu Tablets; YSG, Yangxin Shengmai Granules; YT, Yixinshu Tablets

Figure S7.3: Network map of the effect on hs-CRP, and forest plot of network effect sizes compared with Conventional Medicine treatment. The size of the nodes was proportional to the number of participants included in the trial, and the thickness of lines between the interventions relates to the number of studies for that comparison.

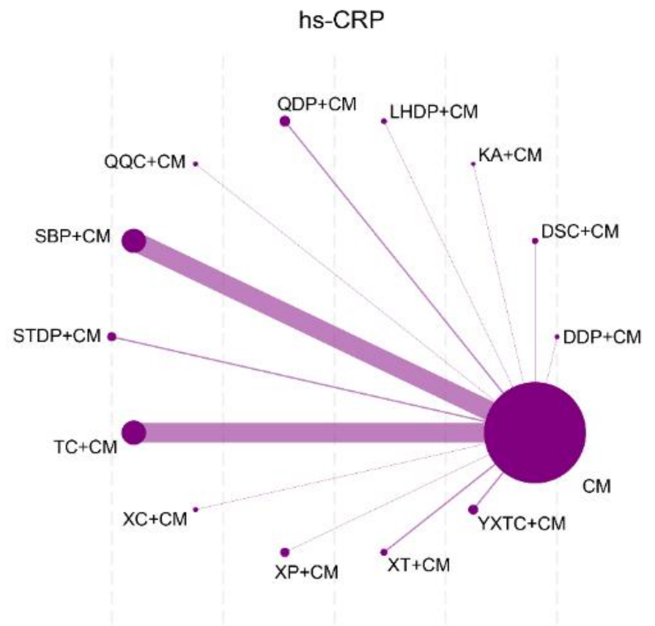

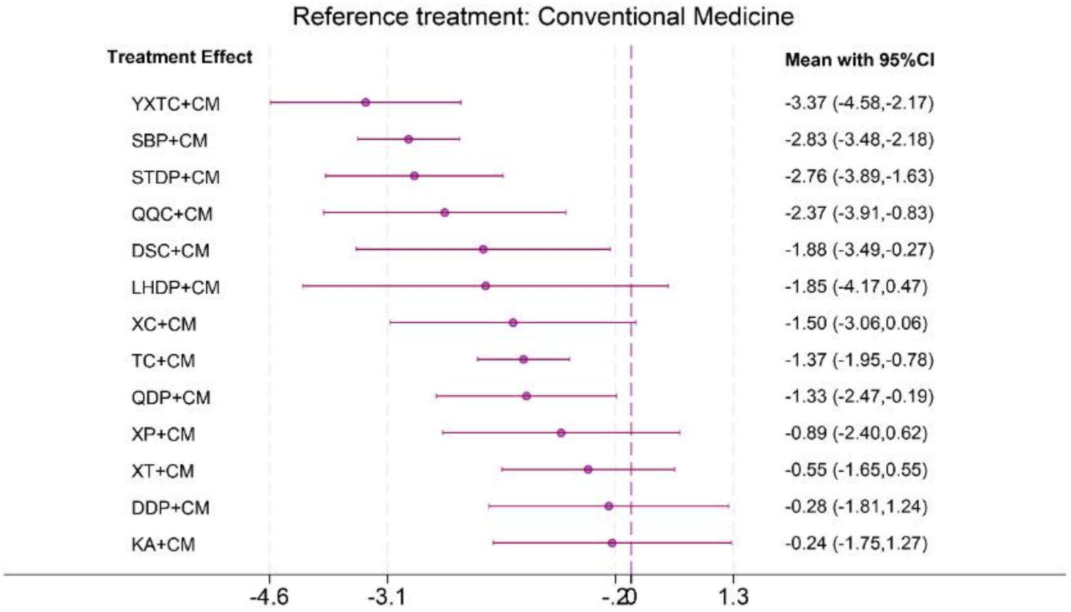

Abbreviations: CM, Conventional Medicine; YXTC, Yindan Xinnao Tong Capsules; SBP, Shexiang Baoxin Pills; STDP, Shexiang Tongxin Dripping Pills; QQC, Qili Qiangxin Capsules; DSC, Dengzhan Shengmai Capsules; LHDP, Liqi Huoxue Dripping Pills; XC, Xinxuekang Capsules; TC, Tongxinluo Capsules; QDP, Qishen Dripping Pills; XP, Xinbao Pills; XT, Xinkeshu Tablets; DDP, Danshen Dripping Pills; KA, Kuanxiong Aerosol.

Figure S7.4: Network map of the effect on ET-1, and forest plot of network effect sizes compared with Conventional Medicine treatment. The size of the nodes was proportional to the number of participants included in the trial, and the thickness of lines between the interventions relates to the number of studies for that comparison.

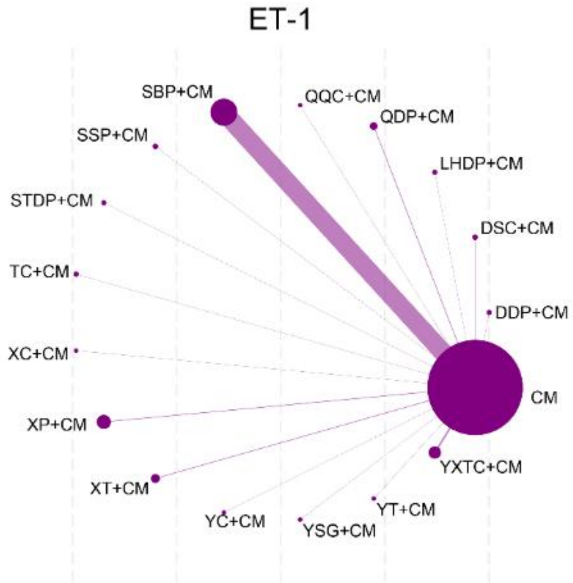

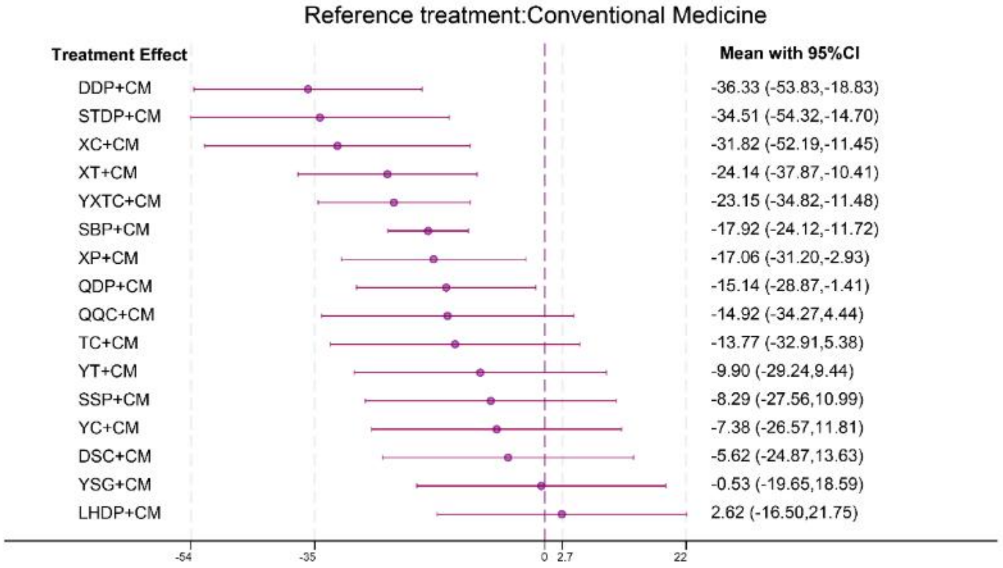

Abbreviations: CM, Conventional Medicine; DDP, Danshen Dripping Pills; STDP,Shexiang Tongxin Dripping Pills; XC, Xinxuekang Capsules; XT, Xinkeshu Tablets; YXTC,Yindan Xinnao Tong Capsules; SBP, Shexiang Baoxin Pills; XP,Xinbao Pills; QDP,Qishen Dripping Pills; QQC,Qili Qiangxin Capsules; TC, Tongxinluo Capsules; YT, Yixinshu Tablets; SSP, Shenxiang Suhe Pills; YC,Yuxintong Capsules; DSC,Dengzhan Shengmai Capsules; YSG, Yangxin Shengmai Granules; LHDP,Liqi Huoxue Dripping Pills.

Figure S7.5: Network map of the effect on NO, and forest plot of network effect sizes compared with Conventional Medicine treatment. The size of the nodes was proportional to the number of participants included in the trial, and the thickness of lines between the interventions relates to the number of studies for that comparison.

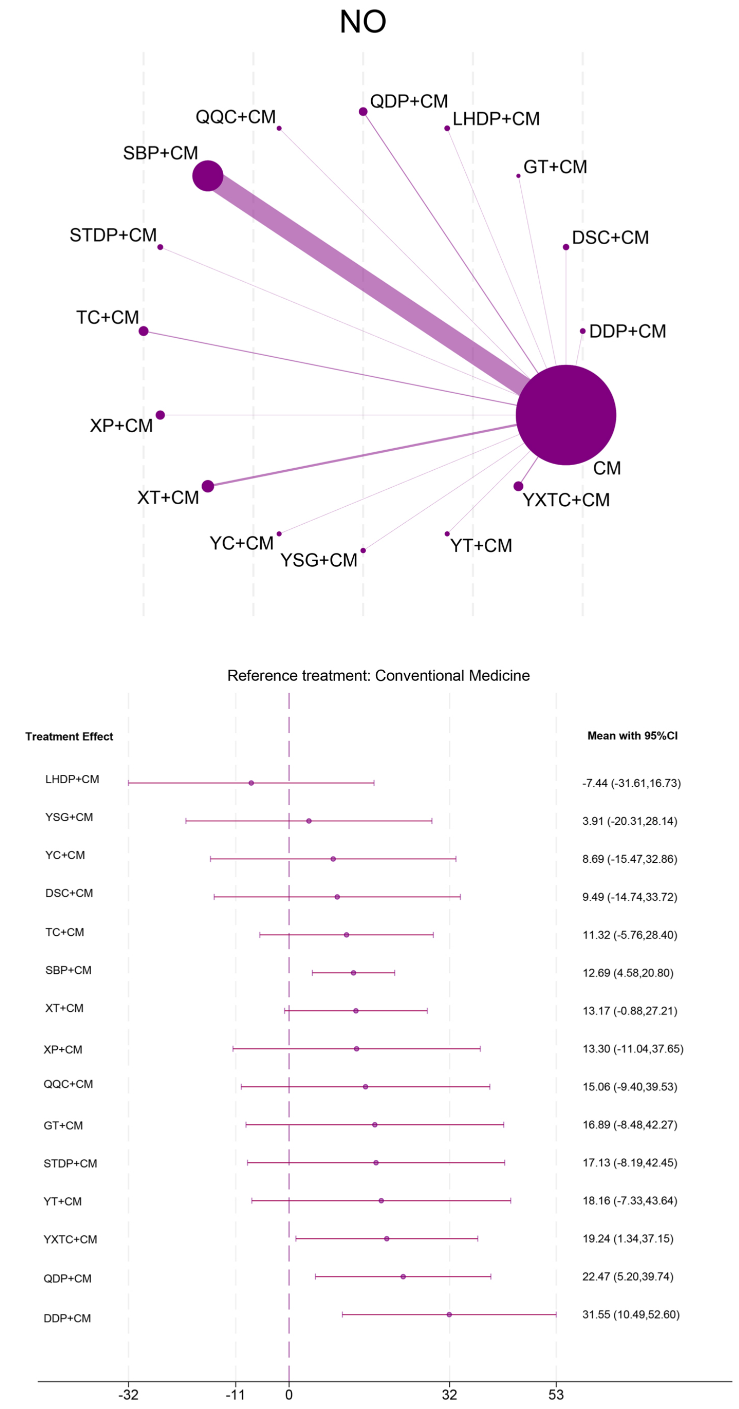

Abbreviations: CM, Conventional Medicine; DDP, Danshen Dripping Pills; QDP, Qishen Dripping Pills; YXTC, Yindan Xinnao Tong Capsules; YT, Yixinshu Tablets; GT, Guanxinning Tablets; STDP, Shexiang Tongxin Dripping Pills; QQC, Qili Qiangxin Capsules; XT, Xinkeshu Tablets; XP, Xinbao Pills; SBP, Shexiang Baoxin Pills; TC, Tongxinluo Capsules; DSC, Dengzhan Shengmai Capsules; YC, Yuxintong Capsules; YSG, Yangxin Shengmai Granules.

Figure S7.6: Network map of the effect on Adverse event, and forest plot of network effect sizes compared with Conventional Medicine treatment. The size of the nodes was proportional to the number of participants included in the trial, and the thickness of lines between the interventions relates to the number of studies for that comparison.

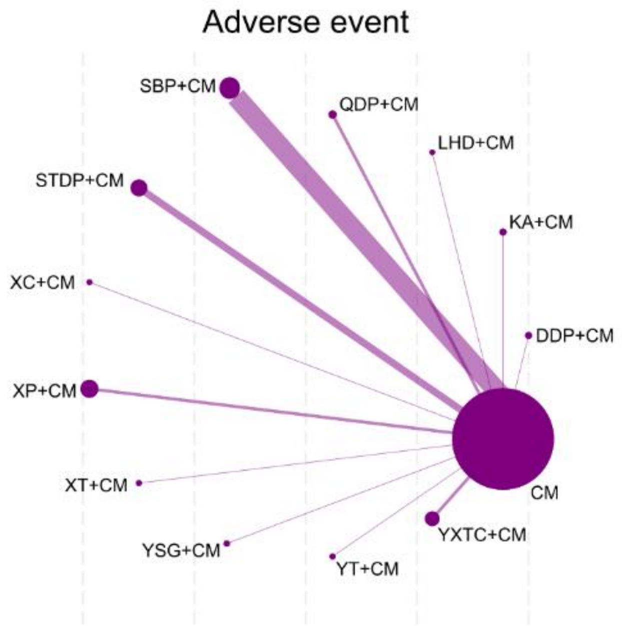

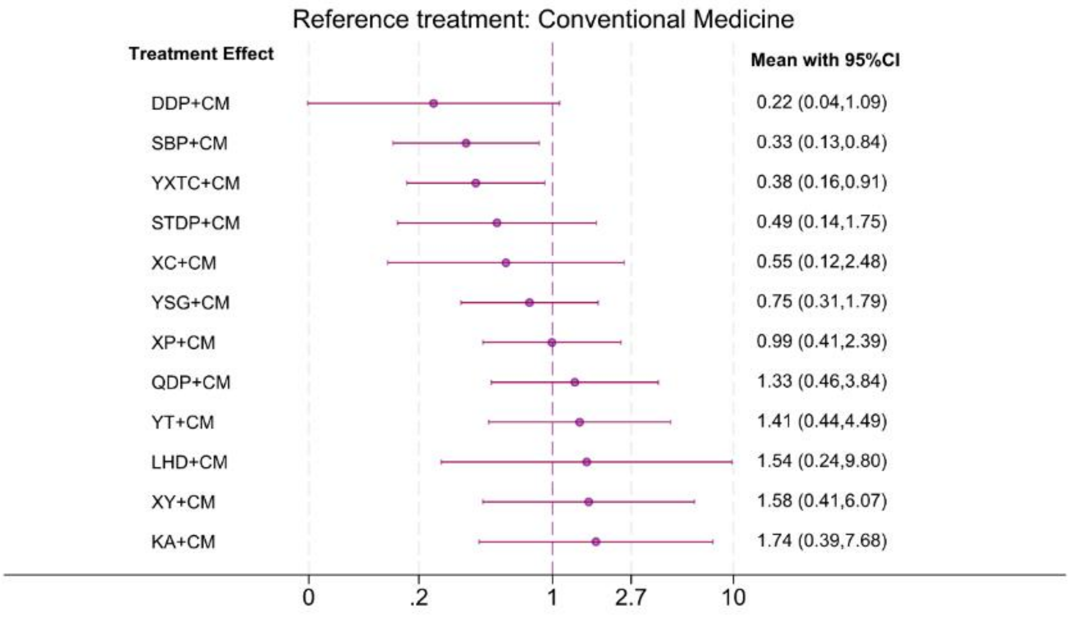

Abbreviations: CM, Conventional Medicine; YXTC, Yindan Xinnao Tong Capsules; DDP, Danshen Dripping Pills; SBP, Shexiang Baoxin Pills; STDP, Shexiang Tongxin Dripping Pills; XC, Xinxuekang Capsules; YSG, Yangxin Shengmai Granules; XP, Xinbao Pills; QDP, Qishen Dripping Pills; LHD, Lingbao Huxin Dan; YT, Yixinshu Tablets; XT, Xinkeshu Tablets; KA, Kuanxiong Aerosol.

Appendix 8: SUCRA and cumulative probability plots

Figure S8.1: Cumulative ranking probability curves for IMR levels of different Commercial Chinese polyherbal preparations combined with Conventional Medicine in the treatment of coronary microvascular disease, within the scope of network meta-analysis. A larger area under the curve indicates a more effective treatment regimen.

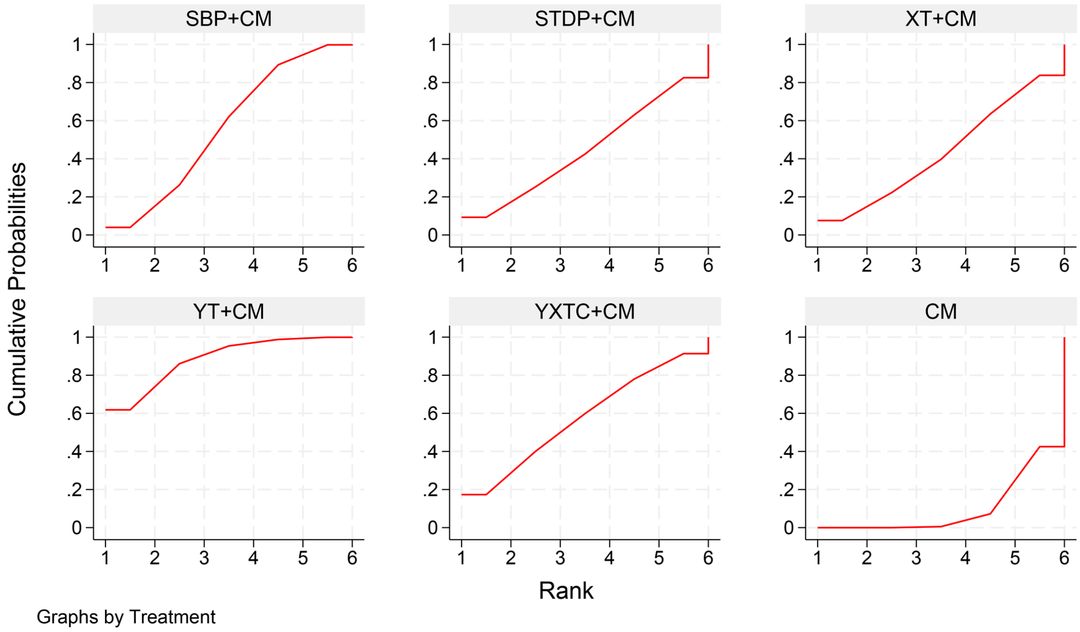

Table S8.1: SUCRA rankings of IMR for different Commercial Chinese polyherbal preparations combined with Conventional Medicine in the treatment of coronary microvascular disease.

Treatment	SUCRA	PrBest	MeanRank
YT+CM	88.4	61.8	1.6
YXTC+CM	57.3	17.3	3.1
SBP+CM	56.3	4	3.2
STDP+CM	44.5	9.3	3.8
XT+CM	43.4	7.6	3.8
CM	10.1	0.0	5.5

Abbreviations: SUCRA, surface under the cumulative ranking curve; CM, Conventional Medicine; YT, Yixinshu Tablets; YXTC, Yindan Xinnao Tong Capsules; SBP, Shexiang Baoxin Pills; STDP, Shexiang Tongxin Dripping Pills; XT, Xinkeshu Tablets.

Figure S8.2: Cumulative ranking probability curves for CFR levels of different Commercial Chinese polyherbal preparations combined with Conventional Medicine in the treatment of coronary microvascular disease, within the scope of network meta-analysis. A larger area under the curve indicates a more effective treatment regimen.

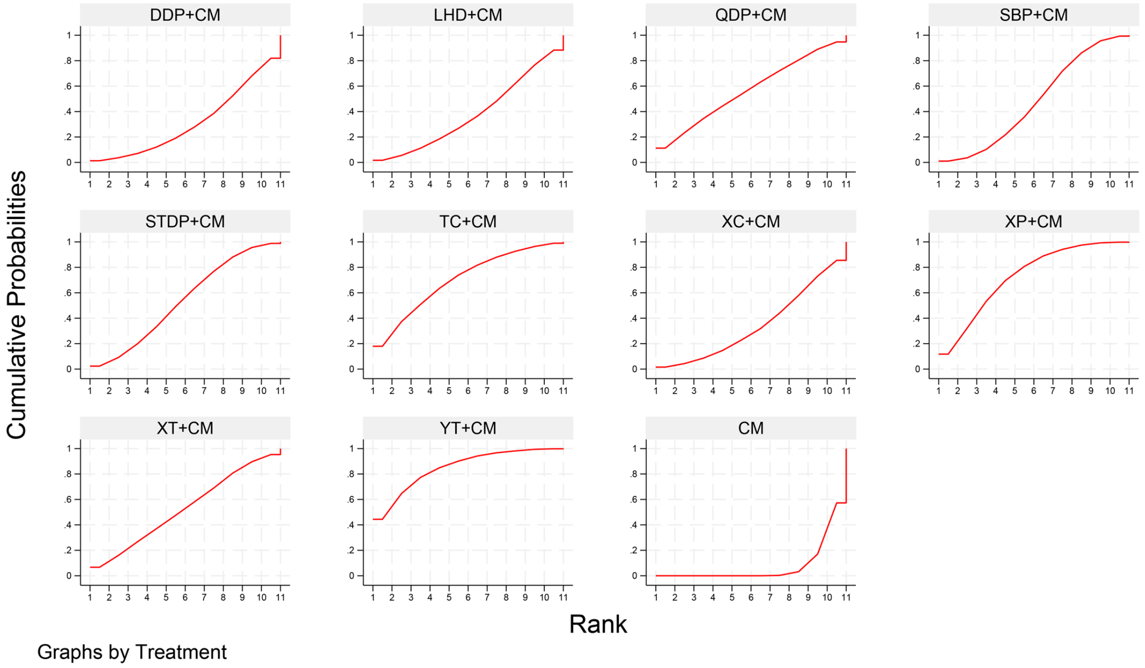

Table S8.2: SUCRA rankings of CFR for different Commercial Chinese polyherbal preparations combined with Conventional Medicine in the treatment of coronary microvascular disease.

Treatment	SUCRA	PrBest	MeanRank
YT+CM	85	44.4	2.5
XP+CM	72.7	11.8	3.7
TC+CM	70.2	18	4
QDP+CM	56.7	11.3	5.3
STDP+CM	53.7	2.3	5.6
XT+CM	52.7	6.7	5.7
SBP+CM	47.9	1	6.2
LHD+CM	37.6	1.7	7.2
XC+CM	34.4	1.5	7.6
DDP+CM	31.2	1.3	7.9
CM	7.8	0	10.2

Abbreviations: SUCRA, surface under the cumulative ranking curve; CM, Conventional Medicine; YT, Yixinshu Tablets; XP, Xinbao Pills; TC, Tongxinluo Capsules; QDP, Qishen Dripping Pills; STDP, Shexiang Tongxin Dripping Pills; XT, Xinkeshu Tablets; SBP, Shexiang Baoxin Pills; LHD, Lingbao Huxin Dan; XC, Xinxuekang Capsules; DDP, Danshen Dripping Pills.

Figure S8.3: Cumulative ranking probability curves for cTFC levels of different Commercial Chinese polyherbal preparations combined with Conventional Medicine in the treatment of coronary microvascular disease, within the scope of network meta-analysis. A larger area under the curve indicates a more effective treatment regimen.

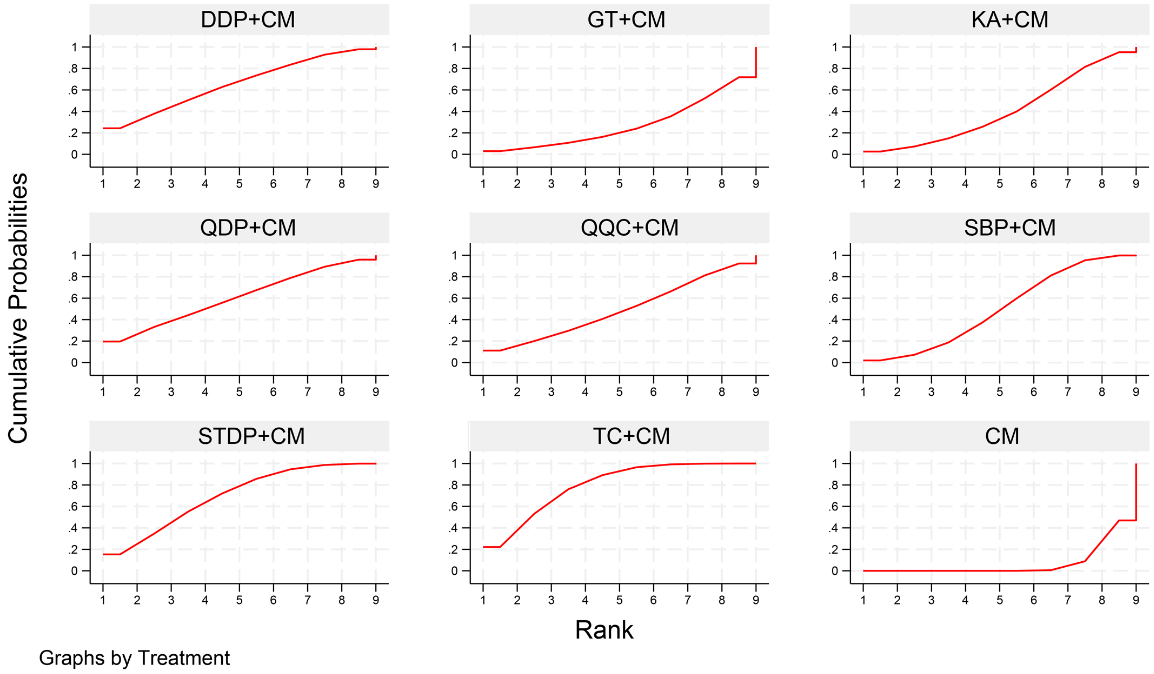

Table S8.3: SUCRA rankings of cTFC for different Commercial Chinese polyherbal preparations combined with Conventional Medicine in the treatment of coronary microvascular disease.

Treatment	SUCRA	PrBest	MeanRank
TC+CM	79.5	22.2	2.6
STDP+CM	69.5	15.3	3.4
DDP+CM	65.4	24.3	3.8
QDP+CM	60.6	19.6	4.2
SBP+CM	50.2	2	5
QQC+CM	49.3	11.2	5.1
KA+CM	40.9	2.6	5.7
GT+CM	27.5	3	6.8
CM	7.1	0	8.4

Abbreviations: SUCRA, surface under the cumulative ranking curve; CM, Conventional Medicine; TC, Tongxinluo Capsules; STDP, Shexiang Tongxin Dripping Pills; DDP, Danshen Dripping Pills; QDP, Qishen Dripping Pills; SBP, Shexiang Baoxin Pills; QQC, Qili Qiangxin Capsules; KA, Kuanxiong Aerosol;
GT, Guanxinning Tablets.

Figure S8.4: Cumulative ranking probability curves for the total effective rate of different Commercial Chinese polyherbal preparations combined with Conventional Medicine in the treatment of coronary microvascular disease, within the scope of network meta-analysis. A larger area under the curve indicates a more effective treatment regimen.

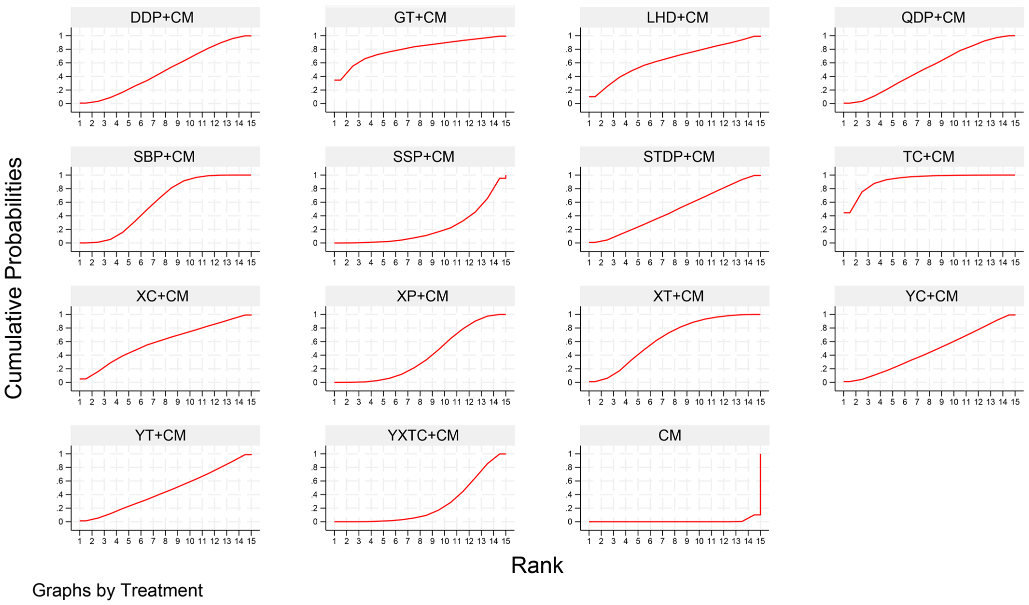

Table S8.8: SUCRA rankings of the total effective rate for different Commercial Chinese polyherbal preparations combined with Conventional Medicine in the treatment of coronary microvascular disease.

Treatment	SUCRA	PrBest	MeanRank
TC+CM	92.2	44.5	2.1
GT+CM	79.8	34.6	3.8
LHD+CM	64.7	10.2	5.9
XT+CM	64.2	1	6
SBP+CM	59.8	0.1	6.6
XC+CM	59.4	5.1	6.7
QDP+CM	52.6	0.6	7.6
DDP+CM	49.2	0.7	8.1
STDP+CM	48.5	0.8	8.2
YC+CM	46.1	1.1	8.5
YT+CM	45.8	1.3	8.6
XP+CM	39.6	0	9.5
YXTC+CM	25.7	0	11.4
SSP+CM	21.8	0	12
CM	0.7	0	14.9

Abbreviations: SUCRA, surface under the cumulative ranking curve; CM, Conventional Medicine; TC, Tongxinluo Capsules; GT, Guanxinning Tablets; LHD, Lingbao Huxin Dan; XT, Xinkeshu Tablets; SBP, Shexiang Baoxin Pills; XC, Xinxuekang Capsules; QDP, Qishen Dripping Pills; DDP, Danshen Dripping Pills; STDP, Shexiang Tongxin Dripping Pills; YC, Yuxintong Capsules; YT, Yixinshu Tablets; XP, Xinbao Pills; YXTC, Yindan Xinnao Tong Capsules; SSP, Shenxiang Suhe Pills.

Figure S8.5: Cumulative ranking probability curves for LVEF of different Commercial Chinese polyherbal preparations combined with Conventional Medicine in the treatment of coronary microvascular disease, within the scope of network meta-analysis. A larger area under the curve indicates a more effective treatment regimen.

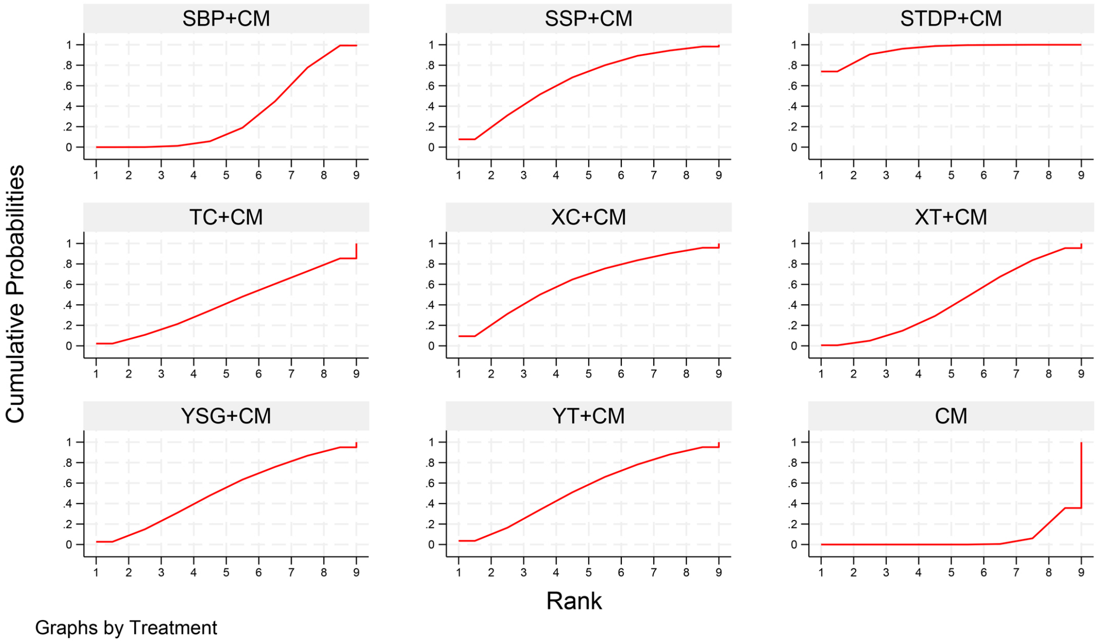

Table S8.4: SUCRA rankings of LVEF for different Commercial Chinese polyherbal preparations combined with Conventional Medicine in the treatment of coronary microvascular disease.

Treatment	SUCRA	PrBest	MeanRank
STDP+CM	94.9	73.9	1.4
SSP+CM	65	7.7	3.8
XC+CM	62.6	9.4	4
YT+CM	54	3.6	4.7
YSG+CM	52.2	2.7	4.8
XT+CM	43	0.6	5.6
TC+CM	42	2.2	5.6
SBP+CM	31	0	6.5
CM	5.3	0	8.6

Abbreviations: SUCRA, surface under the cumulative ranking curve; CM, Conventional Medicine; STDP, Shexiang Tongxin Dripping Pills; SSP, Shenxiang Suhe Pills; XC, Xinxuekang Capsules; YT, Yixinshu Tablets; YSG, Yangxin Shengmai Granules; XT, Xinkeshu Tablets; TC, Tongxinluo Capsules; SBP, Shexiang Baoxin Pills

Figure S8.6: Cumulative ranking probability curves for hs-CRP levels of different Commercial Chinese polyherbal preparations combined with Conventional Medicine in the treatment of coronary microvascular disease, within the scope of network meta-analysis. A larger area under the curve indicates a more effective treatment regimen.

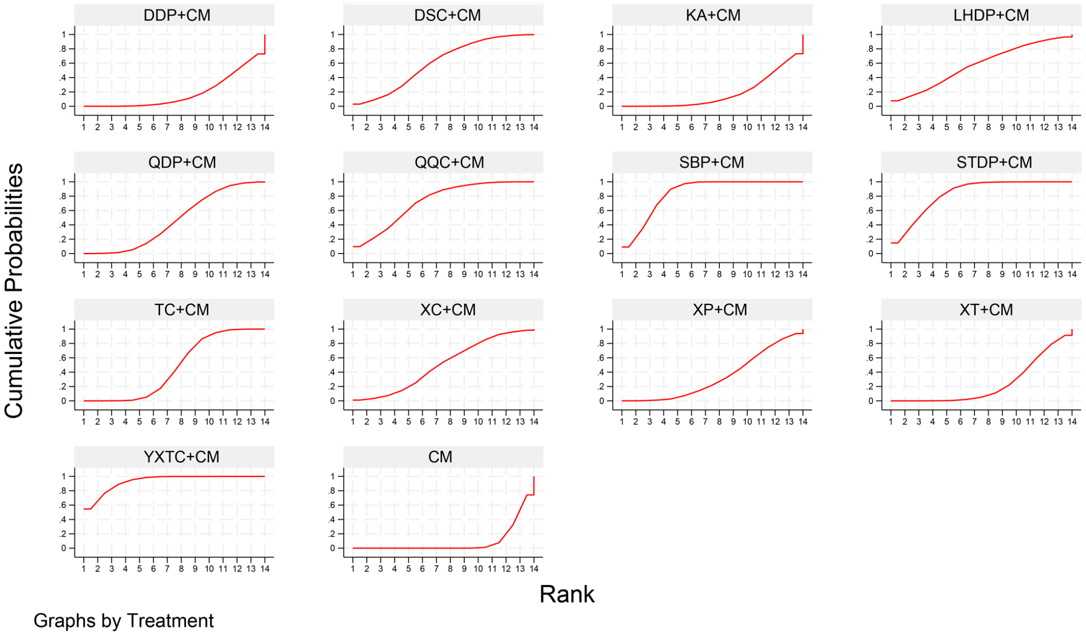

Table S8.6: SUCRA rankings of hs-CRP for different Commercial Chinese polyherbal preparations combined with Conventional Medicine in the treatment of coronary microvascular disease.

Treatment	SUCRA	PrBest	MeanRank
YXTC+CM	93.4	54.6	1.9
SBP+CM	84.5	9.2	3
STDP+CM	83	14.8	3.2
QQC+CM	72.8	9.7	4.5
DSC+CM	60.6	3	6.1
LHDP+CM	57.7	7.6	6.5
XC+CM	50.6	1.1	7.4
TC+CM	47.1	0	7.9
QDP+CM	46.7	0	7.9
XP+CM	33.8	0	9.6
XT+CM	23.9	0	10.9
DDP+CM	18.6	0	11.6
KA+CM	18.2	0	11.6
CM	8.9	0	12.8

Abbreviations: SUCRA, surface under the cumulative ranking curve; CM, Conventional Medicine; YXTC, Yindan Xinnao Tong Capsules; SBP, Shexiang Baoxin Pills; STDP, Shexiang Tongxin Dripping Pills; QQC, Qili Qiangxin Capsules; DSC, Dengzhan Shengmai Capsules; LHDP, Liqi Huoxue Dripping Pills; XC, Xinxuekang Capsules; TC, Tongxinluo Capsules; QDP, Qishen Dripping Pills; XP, Xinbao Pills; XT, Xinkeshu Tablets; DDP, Danshen Dripping Pills; KA, Kuanxiong Aerosol.

Figure S8.7: Cumulative ranking probability curves for ET-1 levels of different Commercial Chinese polyherbal preparations combined with Conventional Medicine in the treatment of coronary microvascular disease, within the scope of network meta-analysis. A larger area under the curve indicates a more effective treatment regimen.

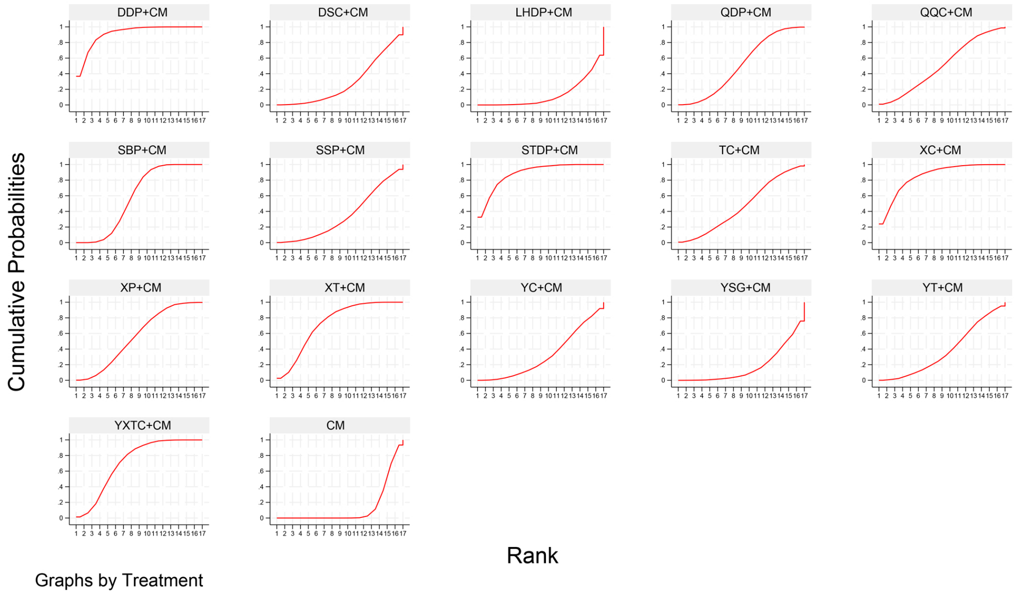

Table S8.7: SUCRA rankings of ET-1 for different Commercial Chinese polyherbal preparations combined with Conventional Medicine in the treatment of coronary microvascular disease.

Treatment	SUCRA	PrBest	MeanRank
DDP+CM	91.4	36.7	2.4
STDP+CM	88.5	32.6	2.8
XC+CM	85.1	23.9	3.4
XT+CM	73.1	2.7	5.3
YXTC+CM	71.8	1.4	5.5
SBP+CM	58.5	0	7.6
XP+CM	56.3	0.2	8
QDP+CM	50.8	0.3	8.9
QQC+CM	50.4	1	8.9
TC+CM	46.7	0.6	9.5
YT+CM	37.9	0.1	10.9
SSP+CM	34.7	0.2	11.5
YC+CM	32.1	0.1	11.9
DSC+CM	28.3	0.1	12.5
YSG+CM	18	0	14.1
LHDP+CM	13.3	0	14.9
CM	13.2	0	14.9

Abbreviations: SUCRA, surface under the cumulative ranking curve; CM, Conventional Medicine; DDP, Danshen Dripping Pills; STDP, Shexiang Tongxin Dripping Pills; XC, Xinxuekang Capsules; XT, Xinkeshu Tablets; YXTC, Yindan Xinnao Tong Capsules; SBP, Shexiang Baoxin Pills; XP, Xinbao Pills; QDP, Qishen Dripping Pills; QQC, Qili Qiangxin Capsules; TC, Tongxinluo Capsules; YT, Yixinshu Tablets; SSP, Shenxiang Suhe Pills; YC, Yuxintong Capsules; DSC, Dengzhan Shengmai Capsules; YSG, Yangxin Shengmai Granules; LHDP, Liqi Huoxue Dripping Pills.

Figure S8.8: Cumulative ranking probability curves for NO levels of different Commercial Chinese polyherbal preparations combined with Conventional Medicine in the treatment of coronary microvascular disease, within the scope of network meta-analysis. A larger area under the curve indicates a more effective treatment regimen.

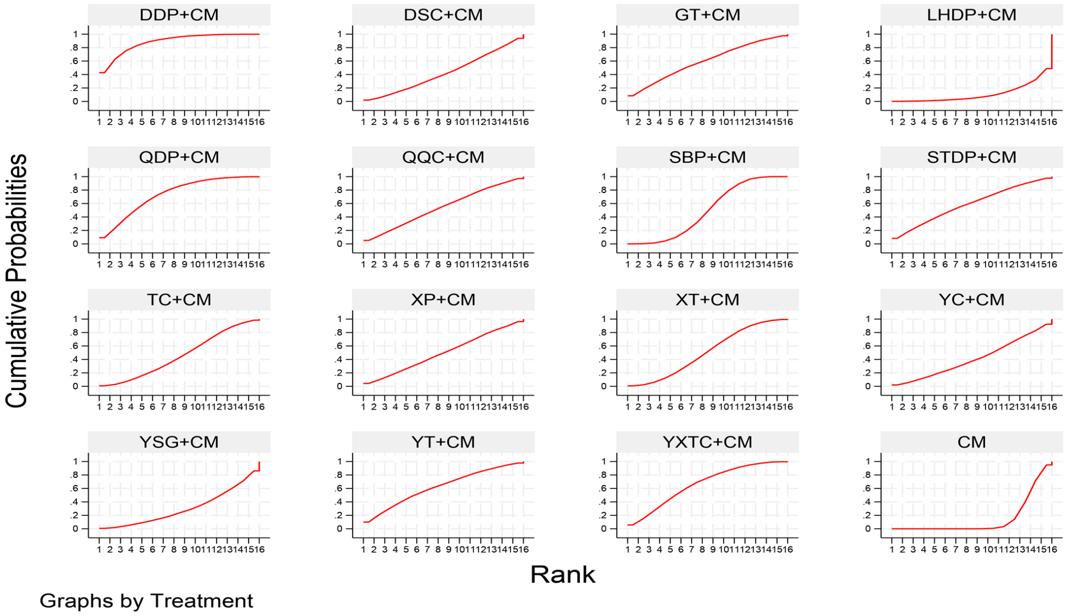

Table S8.8: SUCRA rankings of NO for different Commercial Chinese polyherbal preparations combined with Conventional Medicine in the treatment of coronary microvascular disease.

Treatment	SUCRA	PrBest	MeanRank
DDP+CM	88.5	42.8	2.7
QDP+CM	73.4	9.2	5
YXTC+CM	66.3	5.7	6.1
YT+CM	62.3	10	6.6
GT+CM	59.6	8.4	7.1
STDP+CM	58.7	8.2	7.2
QQC+CM	54.2	5.1	7.9
XT+CM	50.9	0.7	8.4
XP+CM	50	4.3	8.5
SBP+CM	49.7	0	8.5
TC+CM	46.1	0.8	9.1
DSC+CM	42.7	2	9.6
YC+CM	41.1	2.1	9.8
YSG+CM	30.4	0.6	11.4
CM	15	0	13.8
LHDP+CM	11.1	0.1	14.3

Abbreviations: SUCRA, surface under the cumulative ranking curve; CM, Conventional Medicine; DDP, Danshen Dripping Pills; QDP, Qishen Dripping Pills; YXTC, Yindan Xinnao Tong Capsules; YT, Yixinshu Tablets; GT, Guanxinning Tablets; STDP, Shexiang Tongxin Dripping Pills; QQC, Qili Qiangxin Capsules; XT, Xinkeshu Tablets; XP,Xinbao Pills; SBP, Shexiang Baoxin Pills; TC, Tongxinluo Capsules; DSC,Dengzhan Shengmai Capsules; YC,Yuxintong Capsules; YSG,Yangxin Shengmai Granules.

Figure S8.9: Cumulative ranking probability curves for Adverse events of different Commercial Chinese polyherbal preparations combined with Conventional Medicine in the treatment of coronary microvascular disease, within the scope of network meta-analysis.

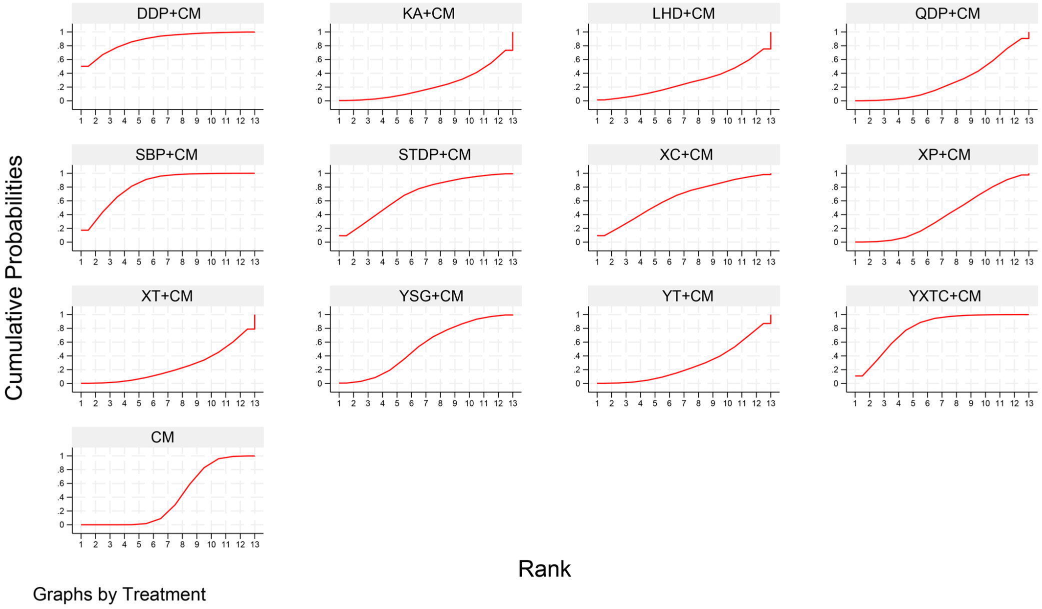

Table S8.9: SUCRA rankings of adverse events for different Commercial Chinese polyherbal preparations combined with Conventional Medicine in the treatment of coronary microvascular disease.

Treatment	SUCRA	PrBest	MeanRank
DDP+CM	88	50.1	2.4
SBP+CM	82.6	17.3	3.1
YXTC+CM	79.8	10.9	3.4
STDP+CM	69	9.3	4.7
XC+CM	63.5	9.4	5.4
YSG+CM	53.6	0.5	6.6
XP+CM	40.6	0.2	8.1
CM	39.7	0	8.2
QDP+CM	29.5	0.1	9.5
LHD+CM	28.4	1.4	9.6
YT+CM	27.9	0.1	9.7
XT+CM	24.5	0.2	10.1
KA+CM	23	0.4	10.2
DDP+CM	88	50.1	2.4
SBP+CM	82.6	17.3	3.1

Abbreviations: SUCRA, surface under the cumulative ranking curve; CM, Conventional Medicine; YXTC, Yindan Xinnao Tong Capsules; DDP, Danshen Dripping Pills; SBP, Shexiang Baoxin Pills; STDP, Shexiang Tongxin Dripping Pills; XC, Xinxuekang Capsules; YSG, Yangxin Shengmai Granules; XP, Xinbao Pills; QDP, Qishen Dripping Pills; LHD, Lingbao Huxin Dan; YT, Yixinshu Tablets; XT, Xinkeshu Tablets; KA, Kuanxiong Aerosol.

Appendix 9: League table of Summary Estimates for different Commercial Chinese polyherbal preparations combined with Conventional Medicine in the treatment of coronary microvascular disease, derived from the meta-analysis of 67 trials.

Table S9.1: IMR

The columns represent the comparison of the row drug class to the column drug class. The rows represent the comparison of the row drug class to the column drug class. The effect estimates are expressed as a mean difference and 95% confidence interval. For example, the standardized mean difference in IMR for Shexiang Baoxin Pill+CM compared to Shexiang Tongxin Dripping Pills+CM is -1.24 (95% confidence interval -10.03 to 7.55). Mean difference <0 favors the drug in the column, and mean difference >0 favors the drug in the row.

Shexiang Baoxin Pill+CM					
-1.24 (-10.03,7.55)	Shexiang Tongxin Dripping Pills+CM				
-1.36 (-9.84,7.13)	-0.12 (-11.27,11.03)	Xinkeshu Tablets+CM			
4.19 (-2.35,10.73)	5.43 (-4.33,15.18)	5.55 (-3.94,15.03)	Yixinshu Tablets+CM		
0.36 (-8.26,8.99)	1.60 (-9.66,12.86)	1.72 (-9.31,12.74)	-3.83 (-13.43,5.78)	Yindan Xinnao Tong Capsules+CM	
-4.74 (-8.27, -1.20)	-3.50 (-11.55,4.55)	-3.38 (-11.10,4.34)	-8.93 (-14.43, -3.42)	-5.10 (-12.97,2.77)	CM

Abbreviations: IMR, index of microcirculatory resistance; CM, Conventional Medicine.

Table S9.2: CFR

The columns represent the comparison of the row drug class to the column drug class. The rows represent the comparison of the row drug class to the column drug class. The effect estimates are expressed as a mean difference and 95% confidence interval. For example, the standardized mean difference in CFR levels for Danshen Dripping Pills+CM compared to Lingbao Huxin Dan+CM is -0.08 (95% confidence interval -1.10 to 0.94). Mean difference <0 favors the drug in the column, and mean difference >0 favors the drug in the row.

Danshen Dripping Pills+CM										
-0.08 (-1.10,0.94)	Lingbao Huxin Dan+CM									
-0.34 (-1.47,0.79)	-0.26 (-1.39,0.87)	Qishen Dripping Pills+CM								
-0.22 (-1.06,0.61)	-0.14 (-0.98,0.70)	0.12 (-0.85,1.09)	Shexiang Baoxin Pill+CM							
-0.28 (-1.17,0.60)	-0.20 (-1.10,0.69)	0.06 (-0.96,1.07)	-0.06 (-0.74,0.62)	Shexiang Tongxin Dripping Pills+CM						
-0.53 (-1.55,0.49)	-0.45 (-1.48,0.58)	-0.19 (-1.33,0.95)	-0.31 (-1.16,0.54)	-0.25 (-1.15,0.66)	Tongxinluo Capsules+CM					
-0.04 (-1.05,0.97)	0.04 (-0.97,1.05)	0.30 (-0.82,1.42)	0.18 (-0.65,1.02)	0.24 (-0.64,1.13)	0.49 (-0.53,1.51)	Xueshuantong Capsules+CM				
-0.53 (-1.42,0.35)	-0.45 (-1.34,0.44)	-0.19 (-1.21,0.82)	-0.31 (-0.99,0.36)	-0.25 (-0.99,0.49)	-0.00 (-0.90,0.89)	-0.49 (-1.37,0.39)	Xinbao Pills+CM			
-0.28 (-1.29,0.73)	-0.20 (-1.22,0.82)	0.06 (-1.07,1.19)	-0.06 (-0.89,0.78)	0.00 (-0.88,0.89)	0.25 (-0.78,1.28)	-0.24 (-1.25,0.77)	0.25 (-0.63,1.14)	Xinkeshu Tablets+CM		
-0.77 (-1.80,0.26)	-0.69 (-1.72,0.34)	-0.43 (-1.57,0.71)	-0.55 (-1.41,0.31)	-0.49 (-1.39,0.42)	-0.24 (-1.28,0.80)	-0.73 (-1.76,0.30)	-0.24 (-1.14,0.67)	-0.49 (-1.52,0.54)	Yixinshu Tablets+CM	
0.28 (-0.43,0.99)	0.36 (-0.36,1.08)	0.62 (-0.25,1.49)	0.50 (0.07,0.93)	0.56 (0.04,1.09)	0.81 (0.08,1.54)	0.32 (-0.39,1.03)	0.81 (0.29,1.33)	0.56 (-0.16,1.28)	1.05 (0.31,1.79)	CM

Abbreviations: CFR, coronary flow reserve; CM, Conventional Medicine.

Table S9.3: cTFC

The columns represent the comparison of the row drug class to the column drug class. The rows represent the comparison of the row drug class to the column drug class. The effect estimates are expressed as a mean difference and 95% confidence interval. For example, the standardized mean difference in cTFC levels for Danshen Dripping Pills+CM compared to Guanxinning Tablets+CM is -6.00 (95% confidence interval -19.33 to 7.33). Mean difference <0 favors the drug in the column, and mean difference >0 favors the drug in the row.

Danshen Dripping Pills+CM								
-6.00 (-19.33,7.33)	Guanxinning Tablets+CM							
-3.61 (-14.99,7.77)	2.39 (-9.29,14.08)	Kuanxiong Aerosol+CM						
-0.87 (-14.13,12.39)	5.13 (-8.39,18.65)	2.74 (-8.87,14.34)	Qishen Dripping Pills+CM					
-2.40 (-15.65,10.84)	3.60 (-9.90,17.10)	1.21 (-10.38,12.79)	-1.53 (-14.96,11.90)	Qili Qiangxin Capsules+CM				
-2.18 (-12.44,8.09)	3.82 (-6.77,14.42)	1.43 (-6.58,9.44)	-1.31 (-11.81,9.20)	0.22 (-10.26,10.71)	Shexiang Baoxin Pill+CM			
0.24 (-10.56,11.04)	6.24 (-4.89,17.38)	3.85 (-4.86,12.56)	1.12 (-9.93,12.16)	2.64 (-8.38,13.67)	2.42 (-4.76,9.60)	Shexiang Tongxin Dripping Pills+CM		
1.44 (-8.60,11.48)	7.44 (-2.94,17.82)	5.05 (-2.67,12.77)	2.31 (-7.98,12.60)	3.84 (-6.42,14.11)	3.62 (-2.32,9.56)	1.20 (-5.67,8.06)	Tongxinluo Capsules+CM	
-8.50 (-17.75,0.74)	-2.50 (-12.11,7.11)	-4.90 (-11.54,1.75)	-7.63 (-17.14,1.88)	-6.10 (-15.59,3.38)	-6.33 (-10.79, -1.86)	-8.75 (-14.37, -3.12)	-9.94 (-13.87, -6.01)	CM

Abbreviations: cTFC, corrected Thrombolysis in Myocardial Infarction Frame Count; CM, Conventional Medicine.

Table S9.4: Total effective rate

The columns represent the comparison of the row drug class to the column drug class. The rows represent the comparison of the row drug class to the column drug class. The effect estimates are expressed as a mean difference and 95% confidence interval. For example, the relative risks for the total effective rate of Danshen Dripping Pills+CM compared to Guanxinning Tablets+CM are 0.87 (95% confidence interval 0.56 to 1.18). Relative risks <1 favor the drug in the row, and odds ratio >1 favors the drug in the column.

Danshen Dripping Pills+CM														
0.87 (0.65,1.18)	Guanxinning Tablets+CM													
0.94 (0.73,1.22)	1.08 (0.76,1.53)	Lingbao Huxin Dan+CM												
0.99 (0.82,1.19)	1.13 (0.84,1.53)	1.05 (0.81,1.35)	Qishen Dripping Pills+CM											
0.98 (0.84,1.13)	1.12 (0.85,1.47)	1.03 (0.82,1.30)	0.99 (0.86,1.13)	Shexiang Baoxin Pill+CM										
1.09 (0.90,1.32)	1.25 (0.92,1.69)	1.15 (0.89,1.50)	1.10 (0.92,1.33)	1.12 (0.97,1.29)	Shenxiang Suhe Pills+CM									
1.00 (0.82,1.22)	1.15 (0.84,1.56)	1.06 (0.81,1.39)	1.01 (0.83,1.23)	1.03 (0.87,1.21)	0.92 (0.75,1.12)	Shexiang Tongxin Dripping Pills+CM								
0.84 (0.69,1.02)	0.96 (0.70,1.31)	0.89 (0.68,1.16)	0.85 (0.70,1.03)	0.86 (0.73,1.01)	0.77 (0.63,0.94)	0.84 (0.68,1.03)	Tongxinluo Capsules+CM							
0.96 (0.76,1.22)	1.10 (0.79,1.54)	1.02 (0.76,1.37)	0.98 (0.77,1.23)	0.99 (0.81,1.21)	0.89 (0.70,1.12)	0.96 (0.75,1.23)	1.15 (0.90,1.47)	Xueshuantong Capsules+CM						
1.02 (0.88,1.19)	1.17 (0.88,1.55)	1.08 (0.86,1.37)	1.03 (0.89,1.20)	1.05 (0.95,1.16)	0.94 (0.80,1.10)	1.02 (0.86,1.21)	1.22 (1.03,1.45)	1.06 (0.86,1.30)	Xinbao Pills+CM					
0.96 (0.81,1.13)	1.10 (0.82,1.46)	1.02 (0.79,1.30)	0.97 (0.82,1.14)	0.98 (0.87,1.11)	0.88 (0.74,1.04)	0.96 (0.80,1.15)	1.14 (0.95,1.37)	0.99 (0.80,1.23)	0.94 (0.83,1.07)	Xinkeshu Tablets+CM				
1.01 (0.82,1.24)	1.15 (0.84,1.58)	1.07 (0.81,1.41)	1.02 (0.83,1.25)	1.04 (0.87,1.23)	0.93 (0.75,1.14)	1.01 (0.81,1.25)	1.21 (0.97,1.50)	1.05 (0.82,1.34)	0.99 (0.83,1.18)	1.05 (0.87,1.27)	Yuxintong Capsules+CM			
1.01 (0.81,1.25)	1.15 (0.84,1.59)	1.07 (0.81,1.42)	1.02 (0.82,1.26)	1.04 (0.86,1.24)	0.93 (0.74,1.15)	1.01 (0.80,1.27)	1.21 (0.96,1.51)	1.05 (0.81,1.35)	0.99 (0.82,1.19)	1.05 (0.86,1.29)	1.00 (0.79,1.26)	Yixinshu Tablets+CM		
1.06 (0.91,1.24)	1.22 (0.92,1.62)	1.13 (0.89,1.43)	1.08 (0.92,1.25)	1.09 (0.98,1.21)	0.98 (0.83,1.14)	1.06 (0.89,1.26)	1.27 (1.07,1.51)	1.10 (0.89,1.36)	1.04 (0.93,1.17)	1.11 (0.97,1.27)	1.05 (0.88,1.26)	1.05 (0.87,1.28)	Yindan Xinnao Tong Capsules+CM	
1.22 (1.07,1.40)	1.40 (1.07,1.83)	1.30 (1.04,1.62)	1.24 (1.09,1.40)	1.25 (1.18,1.33)	1.12 (0.98,1.28)	1.22 (1.05,1.42)	1.46 (1.26,1.70)	1.27 (1.05,1.54)	1.20 (1.11,1.29)	1.28 (1.15,1.41)	1.21 (1.03,1.42)	1.21 (1.02,1.44)	1.15 (1.06,1.25)	CM

Abbreviations: CM, Conventional Medicine.

Table S9.5: LVEF

The columns represent the comparison of the row drug class to the column drug class. The rows represent the comparison of the row drug class to the column drug class. The effect estimates are expressed as a mean difference and 95% confidence interval. For example, the standardized mean difference in LVEF levels for Shexiang Baoxin Pill+CM compared to Shenxiang Suhe Pills+CM is -3.56 (95% confidence interval -9.60 to 2.48). Mean difference <0 favors the drug in the column, and mean difference >0 favors the drug in the row.

Shexiang Baoxin Pill+CM								
-3.56 (-9.60,2.48)	Shenxiang Suhe Pills+CM							
-8.64 (-14.56,-2.72)	-5.08 (-13.04,2.88)	Shexiang Tongxin Dripping Pills+CM						
-1.12 (-7.94,5.69)	2.44 (-6.21,11.09)	7.52 (-1.05,16.08)	Tongxinluo Capsules+CM					
-3.40 (-10.47,3.67)	0.16 (-8.69,9.01)	5.24 (-3.53,14.00)	-2.28 (-11.67,7.12)	Xueshuantong Capsules+CM				
-1.11 (-5.88,3.67)	2.46 (-4.70,9.61)	7.53 (0.48,14.59)	0.02 (-7.80,7.84)	2.29 (-5.75,10.33)	Xinkeshu Tablets+CM			
-2.15 (-8.24,3.93)	1.41 (-6.68,9.49)	6.49 (-1.51,14.48)	-1.03 (-9.71,7.65)	1.25 (-7.63,10.13)	-1.05 (-8.24,6.14)	Yangxin Shengmai Granules+CM		
-2.31 (-8.29,3.66)	1.25 (-6.75,9.25)	6.33 (-1.58,14.24)	-1.19 (-9.79,7.41)	1.09 (-7.72,9.89)	-1.21 (-8.30,5.89)	-0.16 (-8.20,7.88)	Yixinshu Tablets+CM	
2.35 (0.33,4.36)	5.91 (0.21,11.60)	10.99 (5.42,16.55)	3.47 (-3.04,9.98)	5.75 (-1.03,12.52)	3.45 (-0.88,7.78)	4.50 (-1.24,10.24)	4.66 (-0.96,10.28)	CM

Abbreviations: LVEF, Left Ventricular Ejection Fraction; CM, Conventional Medicine.

Table S9.6: hs-CRP

The columns represent the comparison of the row drug class to the column drug class. The rows represent the comparison of the row drug class to the column drug class. The effect estimates are expressed as a mean difference and 95% confidence interval. For example, the standardized mean difference in hs-CRP levels for Danshen Dripping Pills+CM compared to Dengzhan Shengmai Capsules+CM is 1.60 (95% confidence interval -0.62 to 3.82). Mean difference <0 favors the drug in the column, and mean difference >0 favors the drug in the row.

Danshen Dripping Pills+CM													
1.60 (-0.62,3.82)	Dengzhan Shengmai Capsules+CM												
-0.04 (-2.19,2.10)	-1.64 (-3.85,0.57)	Kuanxiong Aerosol+CM											
1.57 (-1.21,4.34)	-0.03 (-2.86,2.80)	1.61 (-1.16,4.38)	Liqi Huoxue Dripping Pills+CM										
1.05 (-0.86,2.95)	-0.55 (-2.53,1.43)	1.09 (-0.81,2.99)	-0.52 (-3.11,2.07)	Qishen Dripping Pills+CM									
2.09 (-0.08,4.25)	0.49 (-1.74,2.72)	2.13 (-0.03,4.29)	0.52 (-2.27,3.31)	1.04 (-0.88,2.96)	Qili Qiangxin Capsules+CM								
2.55 (0.89,4.20)	0.95 (-0.79,2.69)	2.59 (0.94,4.24)	0.98 (-1.43,3.39)	1.50 (0.18,2.82)	0.46 (-1.21,2.13)	Shexiang Baoxin Pill+CM							
2.47 (0.58,4.37)	0.88 (-1.09,2.85)	2.52 (0.63,4.41)	0.91 (-1.68,3.49)	1.43 (-0.18,3.03)	0.39 (-1.52,2.30)	-0.07 (-1.38,1.23)	Shexiang Tongxin Dripping Pills+CM						
1.09 (-0.55,2.72)	-0.51 (-2.23,1.21)	1.13 (-0.50,2.75)	-0.48 (-2.88,1.91)	0.04 (-1.25,1.32)	-1.00 (-2.65,0.65)	-1.46 (-2.34, -0.59)	-1.39 (-2.66, -0.12)	Tongxinluo Capsules+CM					
1.22 (-0.96,3.40)	-0.38 (-2.63,1.87)	1.26 (-0.92,3.44)	-0.35 (-3.15,2.45)	0.17 (-1.77,2.10)	-0.87 (-3.06,1.32)	-1.33 (-3.02,0.36)	-1.26 (-3.18,0.67)	0.13 (-1.54,1.80)	Xueshuantong Capsules+CM				
0.61 (-1.54,2.75)	-0.99 (-3.20,1.22)	0.65 (-1.49,2.79)	-0.96 (-3.73,1.81)	-0.44 (-2.33,1.45)	-1.48 (-3.64,0.68)	-1.94 (-3.58, -0.30)	-1.87 (-3.75,0.02)	-0.48 (-2.10,1.14)	-0.61 (-2.78,1.56)	Xinbao Pills+CM			
0.26 (-1.61,2.14)	-1.33 (-3.29,0.62)	0.31 (-1.56,2.18)	-1.30 (-3.87,1.27)	-0.78 (-2.37,0.80)	-1.82 (-3.72,0.07)	-2.28 (-3.56, -1.01)	-2.21 (-3.79, -0.63)	-0.82 (-2.07,0.42)	-0.95 (-2.86,0.96)	-0.34 (-2.21,1.52)	Xinkeshu Tablets+CM		
3.09 (1.15,5.04)	1.49 (-0.52,3.51)	3.13 (1.20,5.07)	1.52 (-1.09,4.14)	2.04 (0.38,3.71)	1.00 (-0.95,2.96)	0.54 (-0.83,1.92)	0.62 (-1.04,2.27)	2.01 (0.66,3.35)	1.87 (-0.10,3.85)	2.48 (0.55,4.42)	2.83 (1.19,4.46)	Yindan Xinnao Tong Capsules+CM	
-0.28 (-1.81,1.24)	-1.88 (-3.49, -0.27)	-0.24 (-1.75,1.27)	-1.85 (-4.17,0.47)	-1.33 (-2.4, -0.19)	-2.37 (-3.91, -0.83)	-2.83 (-3.48, -2.18)	-2.76 (-3.89, -1.63)	-1.37 (-1.95, -0.78)	-1.50 (-3.06,0.06)	-0.89 (-2.40,0.62)	-0.55 (-1.65,0.55)	-3.37 (-4.58, -2.17)	CM

Abbreviations: hs-CRP, high-sensitivity C-reactive Protein; CM, Conventional Medicine.

Table S9.7: ET-1

The columns represent the comparison of the row drug class to the column drug class. The rows represent the comparison of the row drug class to the column drug class. The effect estimates are expressed as a mean difference and 95% confidence interval. For example, the standardized mean difference in ET-1 levels for Danshen Dripping Pills+CM compared to Dengzhan Shengmai Capsules+CM is -30.71 (95% confidence interval -59.69 to -4.74). Mean difference <0 favors the drug in the column, and mean difference >0 favors the drug in the row.

Danshen Dripping Pills+CM																
-30.71 (-56.69, -4.74)	Dengzhan Shengmai Capsules+CM															
-38.96 (-64.84, -13.07)	-8.24 (-35.38,18.89)	Liqi Huoxue Dripping Pills+CM														
-21.19 (-43.38,0.99)	9.52 (-14.13,33.17)	17.76 (-5.78,41.31)	Qishen Dripping Pills+CM													
-21.42 (-47.46,4.63)	9.30 (-18.00,36.59)	17.54 (-9.67,44.75)	-0.22 (-23.95,23.51)	Qili Qiangxin Capsules+CM												
-18.41 (-36.93,0.11)	12.30 (-7.93,32.52)	20.54 (0.44,40.65)	2.78 (-12.29,17.85)	3.00 (-17.32,23.32)	Shexiang Baoxin Pill+CM											
-28.04 (-54.04, -2.05)	2.67 (-24.58,29.91)	10.91 (-16.24,38.07)	-6.85 (-30.52,16.82)	-6.63 (-33.94,20.69)	-9.63 (-29.88,10.62)	Shenxiang Suhe Pills+CM										
-1.82 (-28.19,24.55)	28.89 (1.27,56.51)	37.13 (9.60,64.67)	19.37 (-4.73,43.47)	19.59 (-8.10,47.28)	16.59 (-4.16,37.35)	26.22 (-1.42,53.86)	Shexiang Tongxin Dripping Pills+CM									
-22.56 (-48.46,3.33)	8.15 (-19.00,35.30)	16.39 (-10.67,43.45)	-1.37 (-24.93,22.19)	-1.15 (-28.37,26.07)	-4.15 (-24.28,15.97)	5.48 (-21.69,32.65)	-20.74 (-48.29,6.81)	Tongxinluo Capsules+CM								
-4.51 (-31.30,22.28)	26.20 (-1.83,54.23)	34.44 (6.50,62.38)	16.68 (-7.89,41.25)	16.90 (-11.20,45.00)	13.90 (-7.40,35.19)	23.53 (-4.52,51.58)	-2.69 (-31.11,25.72)	18.05 (-9.91,46.01)	Xueshuantong Capsules+CM							
-19.27 (-41.69,3.16)	11.44 (-12.44,35.33)	19.69 (-4.09,43.47)	1.93 (-17.78,21.64)	2.15 (-21.82,26.12)	-0.85 (-16.29,14.58)	8.78 (-15.13,32.68)	-17.45 (-41.78,6.89)	3.30 (-20.50,27.10)	-14.75 (-39.55,10.04)	Xinbao Pills+CM						
-12.20 (-34.41,10.02)	18.52 (-5.13,42.16)	26.76 (3.22,50.30)	9.00 (-10.42,28.42)	9.22 (-14.51,32.95)	6.22 (-8.85,21.28)	15.85 (-7.82,39.52)	-10.37 (-34.47,13.73)	10.37 (-13.19,33.93)	-7.68 (-32.25,16.89)	7.07 (-12.64,26.78)	Xinkeshu Tablets+CM					
-28.95 (-54.88, -3.03)	1.76 (-25.42,28.94)	10.00 (-17.08,37.09)	-7.76 (-31.35,15.84)	-7.54 (-34.79,19.71)	-10.54 (-30.70,9.62)	-0.91 (-28.11,26.29)	-27.13 (-54.71,0.44)	-6.39 (-33.49,20.72)	-24.44 (-52.42,3.55)	-9.69 (-33.52,14.15)	-16.76 (-40.35,6.84)	Yuxintong Capsules+CM				
-35.80 (-61.68, -9.92)	-5.09 (-32.22,22.04)	3.15 (-23.89,30.20)	-14.61 (-38.15,8.93)	-14.39 (-41.59,12.82)	-17.39 (-37.49,2.71)	-7.76 (-34.91,19.39)	-33.98 (-61.51, -6.45)	-13.24 (-40.30,13.82)	-31.29 (-59.23, -3.35)	-16.53 (-40.31,7.25)	-23.61 (-47.15, -0.07)	-6.85 (-33.94,20.24)	Yangxin Shengmai Granules+CM			
-26.43 (-52.47, -0.40)	4.28 (-23.01,31.57)	12.53 (-14.67,39.72)	-5.24 (-28.95,18.48)	-5.02 (-32.37,22.34)	-8.02 (-28.32,12.29)	1.61 (-25.69,28.92)	-24.61 (-52.29,3.07)	-3.87 (-31.08,23.35)	-21.92 (-50.00,6.17)	-7.16 (-31.12,16.79)	-14.24 (-37.95,9.48)	2.52 (-24.72,29.76)	9.37 (-17.82,36.57)	Yixinshu Tablets+CM		
-13.18 (-34.12,7.75)	17.53 (-4.98,40.04)	25.77 (3.37,48.18)	8.01 (-10.01,26.03)	8.23 (-14.37,30.83)	5.23 (-7.99,18.45)	14.86 (-7.67,37.39)	-11.36 (-34.35,11.63)	9.38 (-13.04,31.80)	-8.67 (-32.15,14.81)	6.08 (-12.25,24.42)	-0.99 (-19.01,17.03)	15.77 (-6.69,38.23)	22.62 (0.22,45.02)	13.25 (-9.34,35.83)	Yindan Xinnao Tong Capsules+CM	
-36.33 (-53.83, -18.83)	-5.62 (-24.87,13.63)	2.62 (-16.50,21.75)	-15.14 (-28.87, -1.41)	-14.92 (-34.27,4.44)	-17.92 (-24.12, -11.72)	-8.29 (-27.56,10.99)	-34.51 (-54.32, -14.70)	-13.77 (-32.91,5.38)	-31.82 (-52.19, -11.45)	-17.06 (-31.20, -2.93)	-24.14 (-37.87, -10.41)	-7.38 (-26.57,11.81)	-0.53 (-19.65,18.59)	-9.90 (-29.24,9.44)	-23.15 (-34.82, -11.48)	CM

Abbreviations: ET-1, endothelin-1; CM, Conventional Medicine.

Table S9.8: NO

The columns represent the comparison of the row drug class to the column drug class. The rows represent the comparison of the row drug class to the column drug class. The effect estimates are expressed as a mean difference and 95% confidence interval. For example, the standardized mean difference in NO levels for Danshen Dripping Pills+CM compared to Dengzhan Shengmai Capsules+CM is 22.05 (95% confidence interval -9.96 to 54.07). Mean difference <0 favors the drug in the column, and mean difference >0 favors the drug in the row.

Danshen Dripping Pills+CM															
22.05 (-9.96,54.07)	Dengzhan Shengmai Capsules+CM														
14.65 (-18.21,47.51)	-7.40 (-42.49,27.68)	Guanxinning Tablets+CM													
38.99 (7.01,70.97)	16.93 (-17.29,51.16)	24.34 (-10.70,59.38)	Liqi Huoxue Dripping Pills+CM												
9.08 (-18.03,36.19)	-12.98 (-42.73,16.77)	-5.57 (-36.26,25.11)	-29.91 (-59.61,-0.21)	Qishen Dripping Pills+CM											
16.48 (-15.70,48.66)	-5.57 (-40.01,28.86)	1.83 (-33.41,37.08)	-22.50 (-56.90,11.89)	7.41 (-22.54,37.35)	Qili Qiangxin Capsules+CM										
18.85 (-3.57,41.28)	-3.20 (-28.75,22.35)	4.20 (-22.43,30.84)	-20.14 (-45.63,5.36)	9.78 (-9.30,28.85)	2.37 (-23.41,28.14)	Shexiang Baoxin Pill+CM									
14.41 (-18.40,47.23)	-7.64 (-42.68,27.40)	-0.24 (-36.08,35.60)	-24.57 (-59.58,10.43)	5.34 (-25.31,35.98)	-2.07 (-37.28,33.14)	-4.44 (-31.02,22.15)	Shexiang Tongxin Dripping Pills+CM								
20.23 (-6.78,47.23)	-1.83 (-31.47,27.82)	5.58 (-25.01,36.16)	-18.76 (-48.36,10.84)	11.15 (-13.13,35.44)	3.74 (-26.09,33.58)	1.38 (-17.53,20.28)	5.81 (-24.73,36.35)	Tongxinluo Capsules+CM							
18.24 (-13.85,50.33)	-3.81 (-38.16,30.53)	3.59 (-31.57,38.75)	-20.75 (-55.05,13.56)	9.17 (-20.68,39.01)	1.76 (-32.75,36.27)	-0.61 (-26.27,25.05)	3.83 (-31.29,38.95)	-1.99 (-31.72,27.75)	Xinbao Pills+CM						
18.38 (-6.80,43.56)	-3.68 (-31.68,24.33)	3.73 (-25.27,32.73)	-20.61 (-48.56,7.35)	9.30 (-12.95,31.56)	1.90 (-26.31,30.11)	-0.47 (-16.69,15.74)	3.97 (-24.98,32.92)	-1.85 (-23.96,20.26)	0.14 (-27.96,28.24)	Xinkeshu Tablets+CM					
22.85 (-9.11,54.82)	0.80 (-33.43,35.02)	8.20 (-26.84,43.24)	-16.14 (-50.32,18.04)	13.77 (-15.93,43.48)	6.37 (-28.02,40.76)	4.00 (-21.49,29.49)	8.44 (-26.56,43.44)	2.62 (-26.97,32.22)	4.61 (-29.69,38.91)	4.47 (-23.48,32.42)	Yuxintong Capsules+CM				
27.63 (-4.38,59.64)	5.58 (-28.68,39.84)	12.98 (-22.09,48.06)	-11.35 (-45.57,22.86)	18.56 (-11.19,48.30)	11.15 (-23.28,45.58)	8.78 (-16.76,34.32)	13.22 (-21.82,48.26)	7.40 (-22.23,37.04)	9.39 (-24.95,43.73)	9.25 (-18.74,37.25)	4.78 (-29.44,39.00)	Yangxin Shengmai Granules+CM			
13.39 (-19.55,46.33)	-8.66 (-43.83,26.50)	-1.26 (-37.22,34.70)	-25.60 (-60.72,9.52)	4.31 (-26.47,35.09)	-3.09 (-38.42,32.23)	-5.46 (-32.20,21.28)	-1.02 (-36.94,34.90)	-6.84 (-37.51,23.84)	-4.85 (-40.09,30.39)	-4.99 (-34.08,24.11)	-9.46 (-44.58,25.66)	-14.24 (-49.40,20.92)	Yixinshu Tablets+CM		
12.30 (-15.31,39.91)	-9.75 (-39.88,20.37)	-2.35 (-33.40,28.70)	-26.69 (-56.76,3.39)	3.23 (-21.65,28.10)	-4.18 (-34.50,26.13)	-6.55 (-26.20,13.10)	-2.11 (-33.12,28.90)	-7.93 (-32.67,16.82)	-5.94 (-36.16,24.28)	-6.08 (-28.83,16.68)	-10.55 (-40.62,19.53)	-15.33 (-45.45,14.79)	-1.09 (-32.23,30.05)	Yindan Xinnao Tong Capsules+CM	
31.55 (10.49,52.60)	9.49 (-14.74,33.72)	16.89 (-8.48,42.27)	-7.44 (-31.61,16.73)	22.47 (5.20,39.74)	15.06 (-9.40,39.53)	12.69 (4.58,20.80)	17.13 (-8.19,42.45)	11.32 (-5.76,28.40)	13.30 (-11.04,37.65)	13.17 (-0.88,27.21)	8.69 (-15.47,32.86)	3.91 (-20.31,28.14)	18.16 (-7.33,43.64)	19.24 (1.34,37.15)	CM

Abbreviations: NO, nitric oxide; CM, Conventional Medicine.

Table S9.9: Adverse events

The columns represent the comparison of the row drug class to the column drug class. The rows represent the comparison of the row drug class to the column drug class. The effect estimates are expressed as a mean difference and 95% confidence interval. For example, the odds ratios for adverse events of Danshen Dripping Pills+CM compared to Kuanxiong Aerosol+CM are 0.13 (95% confidence interval 0.01 to 1.13). Odds ratio <1 favors the drug in the row, and odds ratio >1 favors the drug in the column.

Danshen Dripping Pills+CM												
0.13 (0.01,1.13)	Kuanxiong Aerosol+CM											
0.14 (0.01,1.65)	1.13 (0.11,12.07)	Lingbao Huxin Dan+CM										
0.17 (0.02,1.13)	1.31 (0.21,8.14)	1.16 (0.14,9.81)	Qishen Dripping Pills+CM									
0.66 (0.10,4.21)	5.22 (0.90,30.11)	4.63 (0.59,36.68)	3.99 (0.97,16.35)	Shexiang Baoxin Pill+CM								
0.45 (0.06,3.43)	3.52 (0.50,24.78)	3.13 (0.33,29.36)	2.69 (0.52,14.03)	0.67 (0.14,3.24)	Shexiang Tongxin Dripping Pills+CM							
0.40 (0.04,3.58)	3.14 (0.38,26.03)	2.79 (0.26,30.24)	2.40 (0.38,15.14)	0.60 (0.10,3.53)	0.89 (0.13,6.36)	Xueshuantong Capsules+CM						
0.22 (0.04,1.38)	1.75 (0.31,9.83)	1.55 (0.20,12.02)	1.34 (0.34,5.30)	0.34 (0.09,1.20)	0.50 (0.11,2.31)	0.56 (0.10,3.17)	Xinbao Pills+CM					
0.14 (0.02,1.13)	1.10 (0.15,8.14)	0.97 (0.10,9.58)	0.84 (0.15,4.65)	0.21 (0.04,1.08)	0.31 (0.05,1.97)	0.35 (0.05,2.62)	0.63 (0.13,3.12)	Xinkeshu Tablets+CM				
0.30 (0.05,1.83)	2.33 (0.42,13.04)	2.07 (0.27,15.96)	1.78 (0.45,7.03)	0.45 (0.12,1.60)	0.66 (0.14,3.07)	0.74 (0.13,4.21)	1.33 (0.39,4.58)	2.12 (0.43,10.54)	Yangxin Shengmai Granules+CM			
0.16 (0.02,1.13)	1.23 (0.19,8.10)	1.09 (0.12,9.68)	0.94 (0.20,4.53)	0.24 (0.05,1.04)	0.35 (0.06,1.94)	0.39 (0.06,2.61)	0.70 (0.16,3.00)	1.12 (0.19,6.61)	0.53 (0.12,2.25)	Yixinshu Tablets+CM		
0.58 (0.09,3.63)	4.61 (0.82,25.90)	4.09 (0.53,31.68)	3.52 (0.89,13.97)	0.88 (0.25,3.17)	1.31 (0.28,6.10)	1.47 (0.26,8.36)	2.63 (0.76,9.11)	4.20 (0.84,20.93)	1.98 (0.57,6.82)	3.75 (0.88,16.01)	Yindan Xinnao Tong Capsules+CM	
0.22 (0.04,1.09)	1.74 (0.39,7.68)	1.54 (0.24,9.80)	1.33 (0.46,3.84)	0.33 (0.13,0.84)	0.49 (0.14,1.75)	0.55 (0.12,2.48)	0.99 (0.41,2.39)	1.58 (0.41,6.07)	0.75 (0.31,1.79)	1.41 (0.44,4.49)	0.38 (0.16,0.91)	CM

Abbreviations: CM, Conventional Medicine.

Appendix 10: CINeMA Assessment

We use the CINeMA framework to evidence certainty, assessing it for each network estimate based on the following criteria:

A: Within-study bias: We classified the overall risk of bias for each study as low risk of bias, the risk of bias as moderate when none of the four assessed risk of bias items were rated as high risk, and the risk of bias as high when one or both items were rated as high risk. See Appendix 4 for the bias assessment. The risk of bias for a pairwise comparison of each drug is shown in Figure S9.1-9.8.

Figure S10.1: Risk of bias contribution by intervention group in IMR.

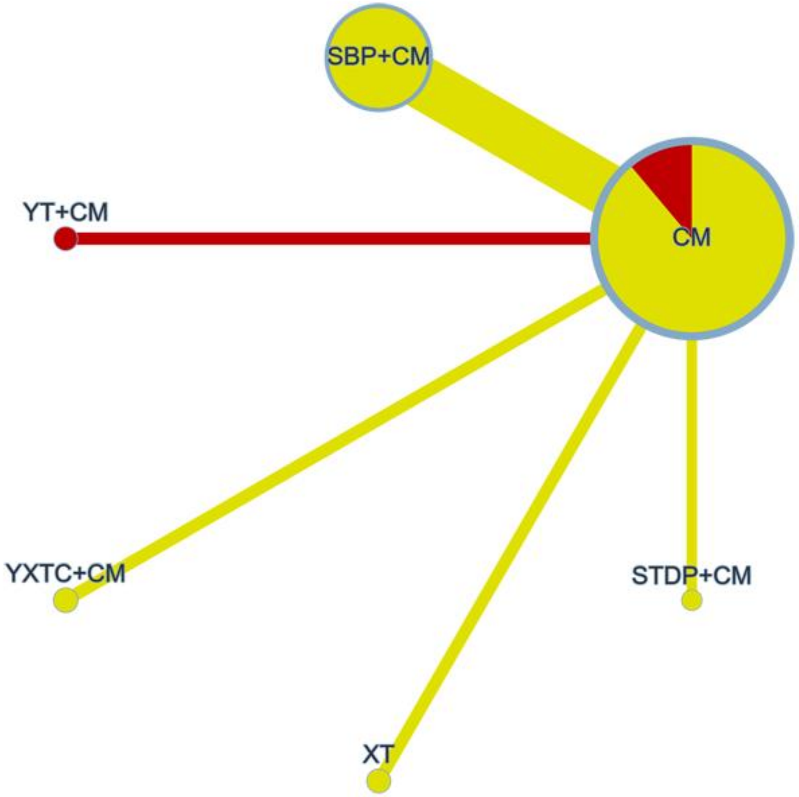

Figure S10.2: Overall risk of bias by treatment comparison in IMR.

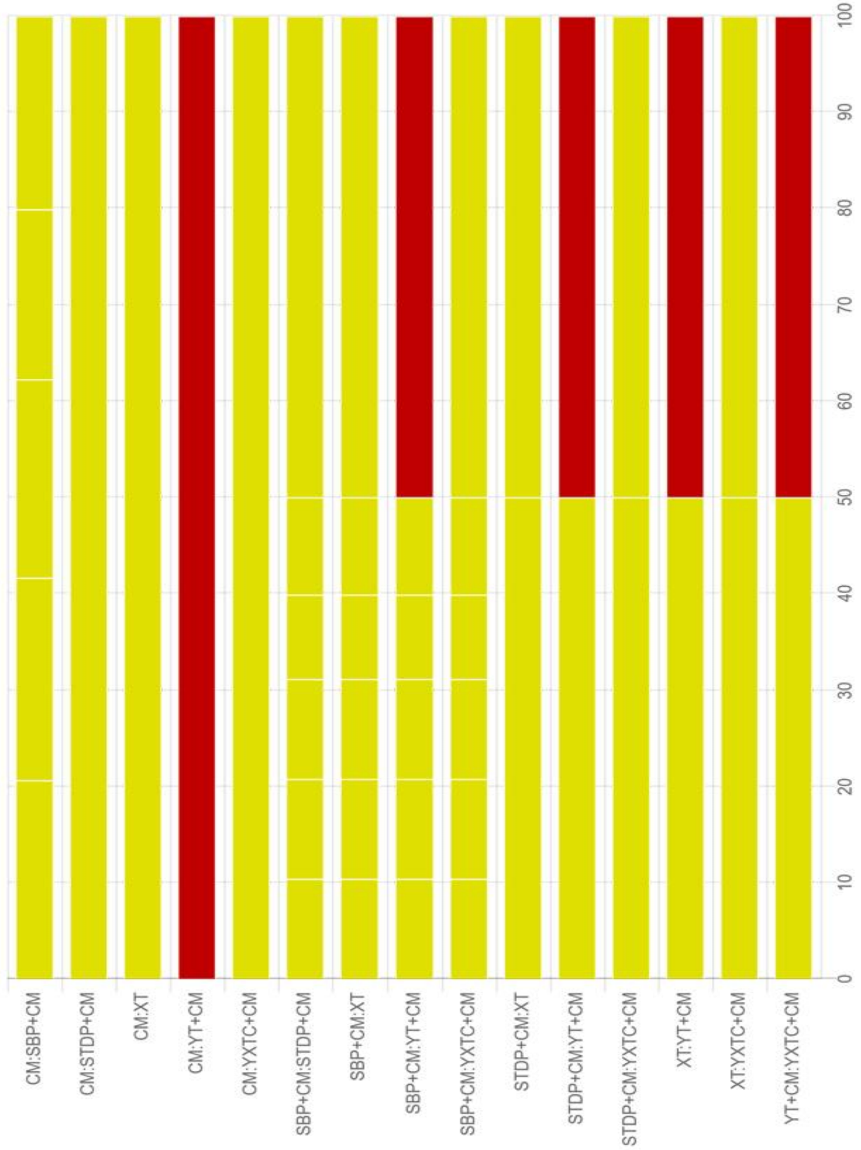

Abbreviations: CM, Conventional Medicine; YT, Yixinshu Tablets; YXTC, Yindan Xinnao Tong Capsules; SBP, Shexiang Baoxin Pills; STDP, Shexiang Tongxin Dripping Pills; XT, Xinkeshu Tablets.

Figure S10.3: Risk of bias contribution by intervention group in CFR.

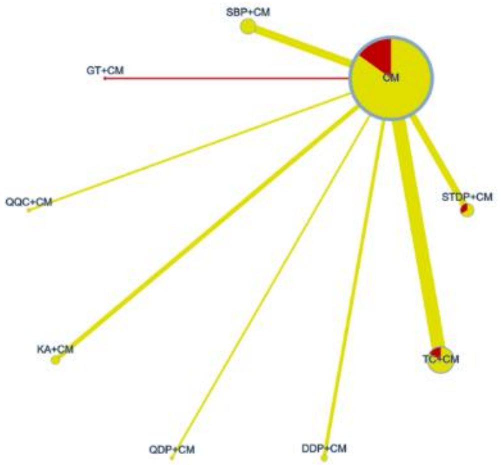

Figure S10.4: Overall risk of bias by treatment comparison in CFR.

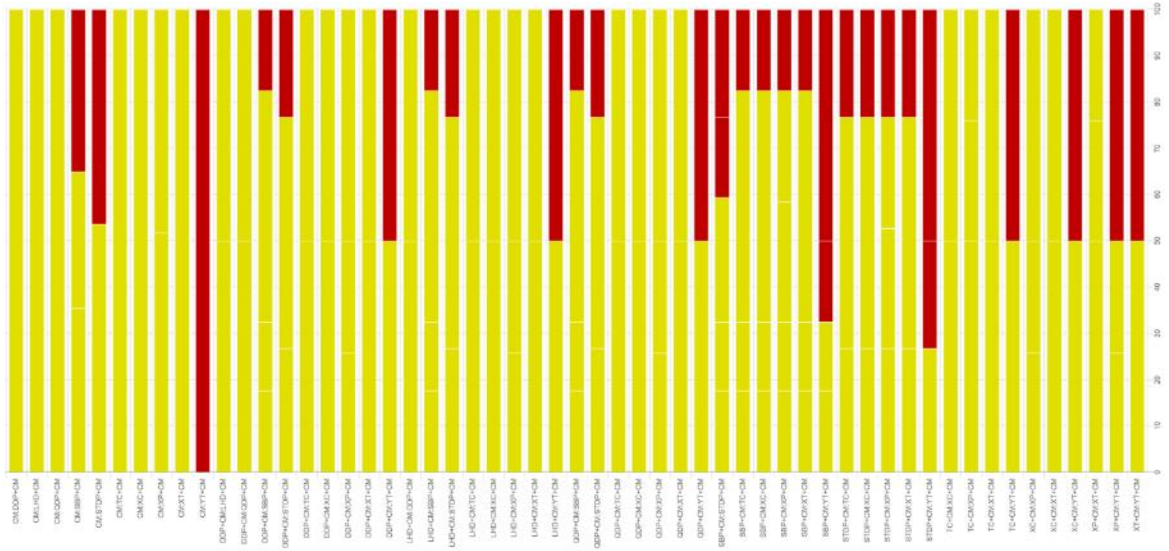

Abbreviations: CFR, coronary flow reserve; CM, Conventional Medicine; YT, Yixinshu Tablets; XP, Xinbao Pills; TC, Tongxinluo Capsules; QDP, Qishen Dripping Pills; STDP, Shexiang Tongxin Dripping Pills; XT, Xinkeshu Tablets; SBP, Shexiang Baoxin Pills; LHD, Lingbao Huxin Dan; XC, Xinxuekang Capsules; DDP, Danshen Dripping Pills.

Figure S10.5: Risk of bias contribution by intervention group in cTFC sneezing

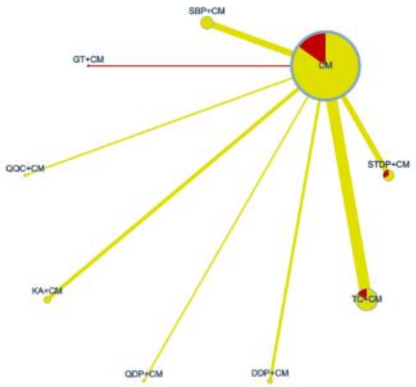

Figure S10.6: Overall risk of bias by treatment comparison in cTFC sneezing

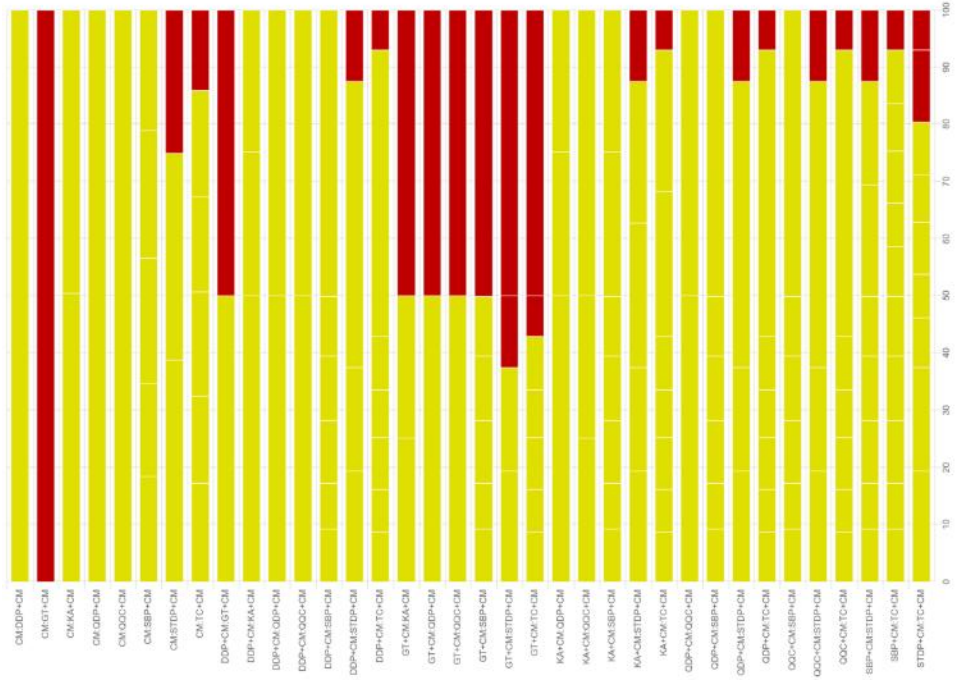

Abbreviations: cTFC, corrected thrombolysis in myocardial infarction frame count; CM, Conventional Medicine; TC, Tongxinluo Capsules; STDP, Shexiang Tongxin Dripping Pills; DDP, Danshen Dripping Pills; QDP, Qishen Dripping Pills; SBP, Shexiang Baoxin Pills; QQC, Qili Qiangxin Capsules; KA, Kuanxiong Aerosol; GT, Guanxinning Tablets.

Figure S10.7: Risk of bias contribution by intervention group in the total effective rate

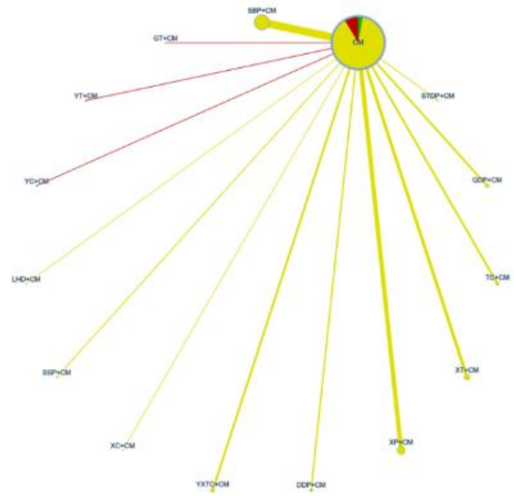

Figure S10.8: Overall risk of bias by treatment comparison in the total effective rate

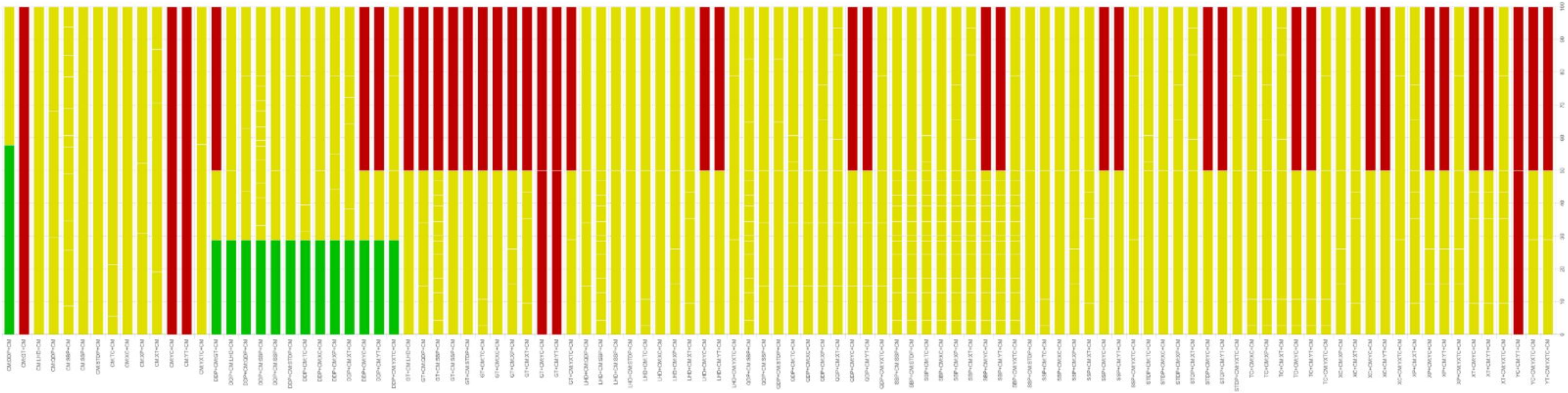

Abbreviations: CM, Conventional Medicine; TC, Tongxinluo Capsules; GT, Guanxinning Tablets; LHD, Lingbao Huxin Dan; XT, Xinkeshu Tablets; SBP, Shexiang Baoxin Pills; XC, Xinxuekang Capsules; QDP, Qishen Dripping Pills; DDP, Danshen Dripping Pills; STDP, Shexiang Tongxin Dripping Pills; YC, Yuxintong Capsules; YT, Yixinshu Tablets; XP, Xinbao Pills; YXTC, Yindan Xinnao Tong Capsules; SSP, Shenxiang Suhe Pills.

Reporting bias: We judged it visually by a funnel plot (Appendix 10).

Indirectness: Indirectness was evaluated by comparing key clinical and methodological characteristics across interventions to ensure the validity of transitivity assumptions. For each Commercial Chinese polyherbal preparation (CCPP), we summarized in Table S10.1 the number of randomized controlled trials (RCTs), total participants, mean age, average disease duration, and overall risk of bias among the included studies.

Table S10.1: Transitivity (Indirectness) Assessment

CPM	Number of RCTs	Total participants	Mean age (years)	Mean disease duration (years)	Overall risk of bias (RoB 2.0)
Shexiang Baoxin Pill	20	1594	55.84±10.44	3.68±1.41	Some concerns 19， High 1
Shexiang Tongxin Dripping Pills	6	541	59.88±7.96	6.6±1.1	Some concerns 5， High 1
Tongxinluo Capsules	9	794	58.87±10.35	-	Some concerns 8， High 1
Xinkeshu Tablets	5	451	62.31±9.84	3.77±1.23	Some concerns 5
Xinbao Pills	3	520	58.59±7.46	5.69±3.70	Some concerns 3
Danshen Dripping Pills	3	308	55.23±11.55	7.30±2.48	Low 1， Some concerns 1， High 1
Yindan Xinnao Tong Capsules	3	343	58.24±11.05	4.19±2.93	Some concerns 3
Qishen Dripping Pills	5	442	58.93±8.05	1.23±0.35	Some concerns 5
Kuanxiong Aerosol	2	164	58.90±9.74	-	Some concerns 2
Xueshuantong Capsules	1	81	60.58 ± 8.11	4.99 ± 1.22	High 1
Yangxin Shengmai Granules	1	92	68.73±6.90	-	High 1
Shenxiang Suhe Pills	1	118	66.94 ± 7.85	-	Some concerns 1
Lingbao Huxin Dan	1	76	62.30 ± 6.22	-	Some concerns 1
Xinxuekang Capsules	1	87	60.6 ± 9.6	-	Some concerns 1
Qili Qiangxin Capsules	1	80	58.0±6.6	-	Some concerns 1
Liqi Huoxue Dripping Pills	1	102	53.40 ± 8.38	-	Some concerns 1
Dengzhan Shengmai Capsules	1	120	57.65 ± 9.90	-	Some concerns 1
Yuxintong Capsules	1	82	53.24±1.32	5.38±0.44	High 1
Yixinshu Tablets	1	84	63.93±3.30	4.89±1.06	High 1
Guanxinning Tablets	1	60	58.15 ± 6.38	-	High 1

Imprecision: We use the CINeMA website to grade the accuracy of each comparison.

Heterogeneity: We assessed the degree of worry by comparing clinical reasoning based on 95% confidence intervals (CIs) while applying the same clinical reasoning framework as for inaccuracy. In particular, we judged the consistency of our findings based on the confidence and prediction intervals associated with clinically important effect sizes. And we used the same thresholds of clinical significance as described above and followed the recommendations automatically provided by CINeMA (https://cinema.ispm.unibe.ch/).

Inconsistency: For inconsistency, we looked at the results for node splitting (Appendix 6), and we saw major problems when p<0.10, but otherwise no problems.

Table S10.2: CINeMA Results of IMR

Comparison	Within-study bias	Reporting bias	Indirectness	Imprecision	Heterogeneity	Incoherence	Confidence rating
CM: SBP+CM	Some concerns	Low risk	No concerns	No concerns	Major concerns	Major concerns	Low
CM: STDP+CM	Some concerns	Low risk	No concerns	Major concerns	No concerns	Major concerns	Low
CM: XT+CM	Some concerns	Low risk	No concerns	Major concerns	No concerns	Major concerns	Low
CM: YT+CM	Major concerns	Low risk	No concerns	No concerns	Major concerns	Major concerns	Low
CM: YXTC+CM	Some concerns	Low risk	No concerns	Major concerns	No concerns	Major concerns	Low
SBP+CM: STDP+CM	Some concerns	Low risk	No concerns	Major concerns	No concerns	Major concerns	Low
SBP+CM: XT+CM	Some concerns	Low risk	No concerns	Major concerns	No concerns	Major concerns	Low
SBP+CM: YT+CM	Some concerns	Low risk	No concerns	Major concerns	No concerns	Major concerns	Low
SBP+CM: YXTC+CM	Some concerns	Low risk	No concerns	Major concerns	No concerns	Major concerns	Low
STDP+CM: XT+CM	Some concerns	Low risk	No concerns	Major concerns	No concerns	Major concerns	Low
STDP+CM:YT+CM	Some concerns	Low risk	No concerns	Major concerns	No concerns	Major concerns	Low
STDP+CM: YXTC+CM	Some concerns	Low risk	No concerns	Major concerns	No concerns	Major concerns	Low
XT+CM:YT+CM	Some concerns	Low risk	No concerns	Major concerns	No concerns	Major concerns	Low
XT+CM: YXTC+CM	Some concerns	Low risk	No concerns	Major concerns	No concerns	Major concerns	Low
YT+CM: YXTC+CM	Some concerns	Low risk	No concerns	Major concerns	No concerns	Major concerns	Low

Abbreviations: IMR, index of microcirculatory resistance; CM, Conventional Medicine; YT, Yixinshu Tablets; YXTC, Yindan Xinnao Tong Capsules; SBP, Shexiang Baoxin Pills; STDP, Shexiang Tongxin Dripping Pills; XT, Xinkeshu Tablets.

Table S10.3: CINeMA Results of CFR

Comparison	Within-study bias	Reporting bias	Indirectness	Imprecision	Heterogeneity	Incoherence	Confidence rating
CM: DDP+CM	Some concerns	Low risk	No concerns	Major concerns	No concerns	Major concerns	Low
CM: LHD+CM	Some concerns	Low risk	No concerns	Major concerns	No concerns	Major concerns	Low
CM: QDP+CM	Some concerns	Low risk	No concerns	Major concerns	No concerns	Major concerns	Low
CM: SBP+CM	Some concerns	Low risk	No concerns	No concerns	Major concerns	Major concerns	Low
CM: STDP+CM	Some concerns	Low risk	No concerns	No concerns	Major concerns	Major concerns	Low
CM: TC+CM	Some concerns	Low risk	No concerns	No concerns	Major concerns	Major concerns	Low
CM: XC+CM	Some concerns	Low risk	No concerns	Major concerns	No concerns	Major concerns	Low
CM: XP+CM	Some concerns	Low risk	No concerns	No concerns	Major concerns	Major concerns	Low
CM: XT+CM	Some concerns	Low risk	No concerns	Major concerns	No concerns	Major concerns	Low
CM: YT+CM	Major concerns	Low risk	No concerns	No concerns	No concerns	Major concerns	Low
DDP+CM: LHD+CM	Some concerns	Low risk	No concerns	Major concerns	No concerns	Major concerns	Low
DDP+CM: QDP+CM	Some concerns	Low risk	No concerns	Major concerns	No concerns	Major concerns	Low
DDP+CM: SBP+CM	Some concerns	Low risk	No concerns	Major concerns	No concerns	Major concerns	Low
DDP+CM: STDP+CM	Some concerns	Low risk	No concerns	Major concerns	No concerns	Major concerns	Low
DDP+CM:TC+CM	Some concerns	Low risk	No concerns	Major concerns	No concerns	Major concerns	Low
DDP+CM: XC+CM	Some concerns	Low risk	No concerns	Major concerns	No concerns	Major concerns	Low
DDP+CM: XP+CM	Some concerns	Low risk	No concerns	Major concerns	No concerns	Major concerns	Low
DDP+CM: XT+CM	Some concerns	Low risk	No concerns	Major concerns	No concerns	Major concerns	Low
DDP+CM: YT+CM	Some concerns	Low risk	No concerns	Major concerns	No concerns	Major concerns	Low
LHD+CM: QDP+CM	Some concerns	Low risk	No concerns	Major concerns	No concerns	Major concerns	Low
LHD+CM: SBP+CM	Some concerns	Low risk	No concerns	Major concerns	No concerns	Major concerns	Low
LHD+CM: STDP+CM	Some concerns	Low risk	No concerns	Major concerns	No concerns	Major concerns	Low
LHD+CM:TC+CM	Some concerns	Low risk	No concerns	Major concerns	No concerns	Major concerns	Low
LHD+CM: XC+CM	Some concerns	Low risk	No concerns	Major concerns	No concerns	Major concerns	Low
LHD+CM: XP+CM	Some concerns	Low risk	No concerns	Major concerns	No concerns	Major concerns	Low
LHD+CM: XT+CM	Some concerns	Low risk	No concerns	Major concerns	No concerns	Major concerns	Low
LHD+CM:YT+CM	Some concerns	Low risk	No concerns	Major concerns	No concerns	Major concerns	Low
QDP+CM: SBP+CM	Some concerns	Low risk	No concerns	Major concerns	No concerns	Major concerns	Low
QDP+CM: STDP+CM	Some concerns	Low risk	No concerns	Major concerns	No concerns	Major concerns	Low
QDP+CM:TC+CM	Some concerns	Low risk	No concerns	Major concerns	No concerns	Major concerns	Low
QDP+CM: XC+CM	Some concerns	Low risk	No concerns	Major concerns	No concerns	Major concerns	Low
QDP+CM: XP+CM	Some concerns	Low risk	No concerns	Major concerns	No concerns	Major concerns	Low
QDP+CM: XT+CM	Some concerns	Low risk	No concerns	Major concerns	No concerns	Major concerns	Low

QDP+CM:YT+CM	Some concerns	Low risk	No concerns	Major concerns	No concerns	Major concerns	Low
SBP+CM: STDP+CM	Some concerns	Low risk	No concerns	Major concerns	No concerns	Major concerns	Low
SBP+CM:TC+CM	Some concerns	Low risk	No concerns	Major concerns	No concerns	Major concerns	Low
SBP+CM: XC+CM	Some concerns	Low risk	No concerns	Major concerns	No concerns	Major concerns	Low
SBP+CM: XP+CM	Some concerns	Low risk	No concerns	Major concerns	No concerns	Major concerns	Low
SBP+CM: XT+CM	Some concerns	Low risk	No concerns	Major concerns	No concerns	Major concerns	Low
SBP+CM:YT+CM	Major concerns	Low risk	No concerns	Major concerns	No concerns	Major concerns	Very low
STDP+CM:TC+CM	Some concerns	Low risk	No concerns	Major concerns	No concerns	Major concerns	Low
STDP+CM: XC+CM	Some concerns	Low risk	No concerns	Major concerns	No concerns	Major concerns	Low
STDP+CM: XP+CM	Some concerns	Low risk	No concerns	Major concerns	No concerns	Major concerns	Low
STDP+CM: XT+CM	Some concerns	Low risk	No concerns	Major concerns	No concerns	Major concerns	Low
STDP+CM:YT+CM	Major concerns	Low risk	No concerns	Major concerns	No concerns	Major concerns	Very low
TC+CM: XC+CM	Some concerns	Low risk	No concerns	Major concerns	No concerns	Major concerns	Low
TC+CM: XP+CM	Some concerns	Low risk	No concerns	Major concerns	No concerns	Major concerns	Low
TC+CM: XT+CM	Some concerns	Low risk	No concerns	Major concerns	No concerns	Major concerns	Low
TC+CM:YT+CM	Some concerns	Low risk	No concerns	Major concerns	No concerns	Major concerns	Low
XC+CM: XP+CM	Some concerns	Low risk	No concerns	Major concerns	No concerns	Major concerns	Low
XC+CM: XT+CM	Some concerns	Low risk	No concerns	Major concerns	No concerns	Major concerns	Low
XC+CM:YT+CM	Some concerns	Low risk	No concerns	Major concerns	No concerns	Major concerns	Low
XP+CM: XT+CM	Some concerns	Low risk	No concerns	Major concerns	No concerns	Major concerns	Low
XP+CM:YT+CM	Some concerns	Low risk	No concerns	Major concerns	No concerns	Major concerns	Low
XT+CM:YT+CM	Some concerns	Low risk	No concerns	Major concerns	No concerns	Major concerns	Low

Abbreviations: CFR, coronary flow reserve; CM, Conventional Medicine; YT, Yixinshu Tablets; XP, Xinbao Pills; TC, Tongxinluo Capsules; QDP, Qishen Dripping Pills; STDP, Shexiang Tongxin Dripping Pills; XT, Xinkeshu Tablets; SBP, Shexiang Baoxin Pills; LHD, Lingbao Huxin Dan; XC, Xinxuekang Capsules; DDP, Danshen Dripping Pills.

Table S10.4: CINeMA Results of cTFC

Comparison	Within-study bias	Reporting bias	Indirectness	Imprecision	Heterogeneity	Incoherence	Confidence rating
CM: DDP+CM	Some concerns	Low risk	No concerns	No concerns	Major concerns	Major concerns	Low
CM: GT+CM	Major concerns	Low risk	No concerns	Major concerns	No concerns	Major concerns	Very low
CM: KA+CM	Some concerns	Low risk	No concerns	Major concerns	No concerns	Major concerns	Low
CM: QDP+CM	Some concerns	Low risk	No concerns	Major concerns	No concerns	Major concerns	Low
CM: QQC+CM	Some concerns	Low risk	No concerns	Major concerns	No concerns	Major concerns	Low
CM: SBP+CM	Some concerns	Low risk	No concerns	No concerns	Major concerns	Major concerns	Low
CM: STDP+CM	Some concerns	Low risk	No concerns	No concerns	Major concerns	Major concerns	Low
CM: TC+CM	Some concerns	Low risk	No concerns	No concerns	Major concerns	Major concerns	Low
DDP+CM:GT+CM	Some concerns	Low risk	No concerns	Major concerns	No concerns	Major concerns	Low
DDP+CM: KA+CM	Some concerns	Low risk	No concerns	Major concerns	No concerns	Major concerns	Low
DDP+CM: QDP+CM	Some concerns	Low risk	No concerns	Major concerns	No concerns	Major concerns	Low
DDP+CM: QQC+CM	Some concerns	Low risk	No concerns	Major concerns	No concerns	Major concerns	Low
DDP+CM: SBP+CM	Some concerns	Low risk	No concerns	Major concerns	No concerns	Major concerns	Low
DDP+CM: STDP+CM	Some concerns	Low risk	No concerns	Major concerns	No concerns	Major concerns	Low
DDP+CM:TC+CM	Some concerns	Low risk	No concerns	Major concerns	No concerns	Major concerns	Low
GT+CM: KA+CM	Some concerns	Low risk	No concerns	Major concerns	No concerns	Major concerns	Low
GT+CM: QDP+CM	Some concerns	Low risk	No concerns	Major concerns	No concerns	Major concerns	Low
GT+CM: QQC+CM	Some concerns	Low risk	No concerns	Major concerns	No concerns	Major concerns	Low
GT+CM:S BP+CM	Major concerns	Low risk	No concerns	Major concerns	No concerns	Major concerns	Very low
GT+CM: STDP+CM	Major concerns	Low risk	No concerns	Major concerns	No concerns	Major concerns	Very low
GT+CM:TC+CM	Major concerns	Low risk	No concerns	Major concerns	No concerns	Major concerns	Very low
KA+CM: QDP+CM	Some concerns	Low risk	No concerns	Major concerns	No concerns	Major concerns	Low
KA+CM: QQC+CM	Some concerns	Low risk	No concerns	Major concerns	No concerns	Major concerns	Low
KA+CM: SBP+CM	Some concerns	Low risk	No concerns	Major concerns	No concerns	Major concerns	Low
KA+CM: STDP+CM	Some concerns	Low risk	No concerns	Major concerns	No concerns	Major concerns	Low
KA+CM:TC+CM	Some concerns	Low risk	No concerns	Major concerns	No concerns	Major concerns	Low
QDP+CM: QQC+CM	Some concerns	Low risk	No concerns	Major concerns	No concerns	Major concerns	Low
QDP+CM: SBP+CM	Some concerns	Low risk	No concerns	Major concerns	No concerns	Major concerns	Low
QDP+CM: STDP+CM	Some concerns	Low risk	No concerns	Major concerns	No concerns	Major concerns	Low
QDP+CM:TC+CM	Some concerns	Low risk	No concerns	Major concerns	No concerns	Major concerns	Low
QQC+CM: SBP+CM	Some concerns	Low risk	No concerns	Major concerns	No concerns	Major concerns	Low

QQC+CM: STDP+CM	Some concerns	Low risk	No concerns	Major concerns	No concerns	Major concerns	Low
QQC+CM:TC+CM	Some concerns	Low risk	No concerns	Major concerns	No concerns	Major concerns	Low
SBP+CM: STDP+CM	Some concerns	Low risk	No concerns	Major concerns	No concerns	Major concerns	Low
SBP+CM:TC+CM	Some concerns	Low risk	No concerns	Major concerns	No concerns	Major concerns	Low
STDP+CM:TC+CM	Some concerns	Low risk	No concerns	Major concerns	No concerns	Major concerns	Low

Abbreviations: cTFC, corrected thrombolysis in myocardial infarction frame count; CM, Conventional Medicine; TC, Tongxinluo Capsules; STDP, Shexiang Tongxin Dripping Pills; DDP, Danshen Dripping Pills; QDP, Qishen Dripping Pills; SBP, Shexiang Baoxin Pills; QQC, Qili Qiangxin Capsules; KA, Kuanxiong Aerosol; GT, Guanxinning Tablets.

Table S10.5: CINeMA Results of Total effective rate

Comparison	Within-study bias	Reporting bias	Indirectness	Imprecision	Heterogeneity	Incoherence	Confidence rating
CM: DDP+CM	Some concerns	Low risk	No concerns	No concerns	No concerns	Major concerns	Low
CM: GT+CM	Some concerns	Low risk	No concerns	No concerns	No concerns	Major concerns	Moderate
CM: LHD+CM	Some concerns	Low risk	No concerns	No concerns	No concerns	Major concerns	Moderate
CM: QDP+CM	Some concerns	Low risk	No concerns	No concerns	No concerns	Major concerns	Low
CM: SBP+CM	Some concerns	Low risk	No concerns	No concerns	No concerns	Major concerns	Moderate
CM: SSP+CM	Some concerns	Low risk	No concerns	Major concerns	No concerns	Major concerns	Low
CM: STDP+CM	Some concerns	Low risk	No concerns	No concerns	No concerns	Major concerns	Low
CM: TC+CM	Some concerns	Low risk	No concerns	No concerns	No concerns	Major concerns	Moderate
CM: XC+CM	Some concerns	Low risk	No concerns	No concerns	No concerns	Major concerns	Low
CM: XP+CM	Some concerns	Low risk	No concerns	No concerns	No concerns	Major concerns	Low
CM: XT+CM	Some concerns	Low risk	No concerns	No concerns	No concerns	Major concerns	Low
CM: YC+CM	Major concerns	Low risk	No concerns	No concerns	No concerns	Major concerns	Low
CM: YT+CM	Major concerns	Low risk	No concerns	No concerns	No concerns	Major concerns	Low
CM: YXTC+CM	Some concerns	Low risk	No concerns	No concerns	No concerns	Major concerns	Low
DDP+CM:GT+CM	Major concerns	Low risk	No concerns	Major concerns	No concerns	Major concerns	Very low
DDP+CM: LHD+CM	Some concerns	Low risk	No concerns	Major concerns	No concerns	Major concerns	Low
DDP+CM: QDP+CM	Some concerns	Low risk	No concerns	Major concerns	No concerns	Major concerns	Low
DDP+CM: SBP+CM	Some concerns	Low risk	No concerns	Major concerns	No concerns	Major concerns	Low
DDP+CM: SSP+CM	Some concerns	Low risk	No concerns	Major concerns	No concerns	Major concerns	Low
DDP+CM: STDP+CM	Some concerns	Low risk	No concerns	Major concerns	No concerns	Major concerns	Low
DDP+CM:TC+CM	Some concerns	Low risk	No concerns	Major concerns	No concerns	Major concerns	Low
DDP+CM: XC+CM	Some concerns	Low risk	No concerns	Major concerns	No concerns	Major concerns	Low
DDP+CM: XP+CM	Some concerns	Low risk	No concerns	Major concerns	No concerns	Major concerns	Low
DDP+CM: XT+CM	Some concerns	Low risk	No concerns	Major concerns	No concerns	Major concerns	Low
DDP+CM: YC+CM	Major concerns	Low risk	No concerns	Major concerns	No concerns	Major concerns	Very low
DDP+CM:YT+CM	Major concerns	Low risk	No concerns	Major concerns	No concerns	Major concerns	Very low
DDP+CM: YXTC+CM	Some concerns	Low risk	No concerns	Major concerns	No concerns	Major concerns	Low
GT+CM: LHD+CM	Some concerns	Low risk	No concerns	Major concerns	No concerns	Major concerns	Low
GT+CM: QDP+CM	Some concerns	Low risk	No concerns	Major concerns	No concerns	Major concerns	Low
GT+CM: SBP+CM	Some concerns	Low risk	No concerns	Major concerns	No concerns	Major concerns	Low
GT+CM: SSP+CM	Some concerns	Low risk	No concerns	Major concerns	No concerns	Major concerns	Low

GT+CM: STDP+CM	Some concerns	Low risk	No concerns	Major concerns	No concerns	Major concerns	Low
GT+CM:TC+CM	Some concerns	Low risk	No concerns	Major concerns	No concerns	Major concerns	Low
GT+CM: XC+CM	Some concerns	Low risk	No concerns	Major concerns	No concerns	Major concerns	Low
GT+CM: XP+CM	Some concerns	Low risk	No concerns	Major concerns	No concerns	Major concerns	Low
GT+CM: XT+CM	Some concerns	Low risk	No concerns	Major concerns	No concerns	Major concerns	Low
GT+CM: YC+CM	Major concerns	Low risk	No concerns	Major concerns	No concerns	Major concerns	Very low
GT+CM:YT+CM	Major concerns	Low risk	No concerns	Major concerns	No concerns	Major concerns	Very low
GT+CM: YXTC+CM	Some concerns	Low risk	No concerns	Major concerns	No concerns	Major concerns	Low
LHD+CM: QDP+CM	Some concerns	Low risk	No concerns	Major concerns	No concerns	Major concerns	Low
LHD+CM: SBP+CM	Some concerns	Low risk	No concerns	Major concerns	No concerns	Major concerns	Low
LHD+CM: SSP+CM	Some concerns	Low risk	No concerns	Major concerns	No concerns	Major concerns	Low
LHD+CM: STDP+CM	Some concerns	Low risk	No concerns	Major concerns	No concerns	Major concerns	Low
LHD+CM:TC+CM	Some concerns	Low risk	No concerns	Major concerns	No concerns	Major concerns	Low
LHD+CM: XC+CM	Some concerns	Low risk	No concerns	Major concerns	No concerns	Major concerns	Low
LHD+CM: XP+CM	Some concerns	Low risk	No concerns	Major concerns	No concerns	Major concerns	Low
LHD+CM: XT+CM	Some concerns	Low risk	No concerns	Major concerns	No concerns	Major concerns	Low
LHD+CM: YC+CM	Some concerns	Low risk	No concerns	Major concerns	No concerns	Major concerns	Low
LHD+CM:YT+CM	Some concerns	Low risk	No concerns	Major concerns	No concerns	Major concerns	Low
LHD+CM: YXTC+CM	Some concerns	Low risk	No concerns	Major concerns	No concerns	Major concerns	Low
QDP+CM: SBP+CM	Some concerns	Low risk	No concerns	Major concerns	No concerns	Major concerns	Low
QDP+CM: SSP+CM	Some concerns	Low risk	No concerns	Major concerns	No concerns	Major concerns	Low
QDP+CM: STDP+CM	Some concerns	Low risk	No concerns	Major concerns	No concerns	Major concerns	Low
QDP+CM:TC+CM	Some concerns	Low risk	No concerns	Major concerns	No concerns	Major concerns	Low
QDP+CM: XC+CM	Some concerns	Low risk	No concerns	Major concerns	No concerns	Major concerns	Low
QDP+CM: XP+CM	Some concerns	Low risk	No concerns	Major concerns	No concerns	Major concerns	Low
QDP+CM: XT+CM	Some concerns	Low risk	No concerns	Major concerns	No concerns	Major concerns	Low
QDP+CM: YC+CM	Some concerns	Low risk	No concerns	Major concerns	No concerns	Major concerns	Low
QDP+CM:YT+CM	Some concerns	Low risk	No concerns	Major concerns	No concerns	Major concerns	Low
QDP+CM: YXTC+CM	Some concerns	Low risk	No concerns	Major concerns	No concerns	Major concerns	Low
SBP+CM: SSP+CM	Some concerns	Low risk	No concerns	Major concerns	No concerns	Major concerns	Low
SBP+CM: STDP+CM	Some concerns	Low risk	No concerns	Major concerns	No concerns	Major concerns	Low
SBP+CM:TC+CM	Some concerns	Low risk	No concerns	Major concerns	No concerns	Major concerns	Low
SBP+CM: XC+CM	Some concerns	Low risk	No concerns	Major concerns	No concerns	Major concerns	Low
SBP+CM: XP+CM	Some concerns	Low risk	No concerns	Major concerns	No concerns	Major concerns	Low

SBP+CM: XT+CM	Some concerns	Low risk	No concerns	Major concerns	No concerns	Major concerns	Low
SBP+CM: YC+CM	Some concerns	Low risk	No concerns	Major concerns	No concerns	Major concerns	Low
SBP+CM:YT+CM	Some concerns	Low risk	No concerns	Major concerns	No concerns	Major concerns	Low
SBP+CM: YXTC+CM	Some concerns	Low risk	No concerns	Major concerns	No concerns	Major concerns	Low
SSP+CM: STDP+CM	Some concerns	Low risk	No concerns	Major concerns	No concerns	Major concerns	Low
SSP+CM:TC+CM	Some concerns	Low risk	No concerns	No concerns	No concerns	Major concerns	Moderate
SSP+CM: XC+CM	Some concerns	Low risk	No concerns	Major concerns	No concerns	Major concerns	Low
SSP+CM: XP+CM	Some concerns	Low risk	No concerns	Major concerns	No concerns	Major concerns	Low
SSP+CM: XT+CM	Some concerns	Low risk	No concerns	Major concerns	No concerns	Major concerns	Low
SSP+CM: YC+CM	Some concerns	Low risk	No concerns	Major concerns	No concerns	Major concerns	Low
SSP+CM:YT+CM	Some concerns	Low risk	No concerns	Major concerns	No concerns	Major concerns	Low
SSP+CM: YXTC+CM	Some concerns	Low risk	No concerns	Major concerns	No concerns	Major concerns	Low
STDP+CM:TC+CM	Some concerns	Low risk	No concerns	Major concerns	No concerns	Major concerns	Low
STDP+CM: XC+CM	Some concerns	Low risk	No concerns	Major concerns	No concerns	Major concerns	Low
STDP+CM: XP+CM	Some concerns	Low risk	No concerns	Major concerns	No concerns	Major concerns	Low
STDP+CM: XT+CM	Some concerns	Low risk	No concerns	Major concerns	No concerns	Major concerns	Low
STDP+CM: YC+CM	Some concerns	Low risk	No concerns	Major concerns	No concerns	Major concerns	Low
STDP+CM:YT+CM	Some concerns	Low risk	No concerns	Major concerns	No concerns	Major concerns	Low
STDP+CM:YXTC+CM	Some concerns	Low risk	No concerns	Major concerns	No concerns	Major concerns	Low
TC+CM: XC+CM	Some concerns	Low risk	No concerns	Major concerns	No concerns	Major concerns	Low
TC+CM: XP+CM	Some concerns	Low risk	No concerns	No concerns	No concerns	Major concerns	Moderate
TC+CM: XT+CM	Some concerns	Low risk	No concerns	Major concerns	No concerns	Major concerns	Low
TC+CM: YC+CM	Some concerns	Low risk	No concerns	Major concerns	No concerns	Major concerns	Low
TC+CM:YT+CM	Some concerns	Low risk	No concerns	Major concerns	No concerns	Major concerns	Low
TC+CM: YXTC+CM	Some concerns	Low risk	No concerns	No concerns	No concerns	Major concerns	Moderate
XC+CM: XP+CM	Some concerns	Low risk	No concerns	Major concerns	No concerns	Major concerns	Low
XC+CM: XT+CM	Some concerns	Low risk	No concerns	Major concerns	No concerns	Major concerns	Low
XC+CM: YC+CM	Some concerns	Low risk	No concerns	Major concerns	No concerns	Major concerns	Low
XC+CM:YT+CM	Some concerns	Low risk	No concerns	Major concerns	No concerns	Major concerns	Low
XC+CM: YXTC+CM	Some concerns	Low risk	No concerns	Major concerns	No concerns	Major concerns	Low
XP+CM: XT+CM	Some concerns	Low risk	No concerns	Major concerns	No concerns	Major concerns	Low
XP+CM: YC+CM	Some concerns	Low risk	No concerns	Major concerns	No concerns	Major concerns	Low
XP+CM:YT+CM	Some concerns	Low risk	No concerns	Major concerns	No concerns	Major concerns	Low
XP+CM: YXTC+CM	Some concerns	Low risk	No concerns	Major concerns	No concerns	Major concerns	Low

XT+CM: YC+CM	Some concerns	Low risk	No concerns	Major concerns	No concerns	Major concerns	Low
XT+CM:YT+CM	Some concerns	Low risk	No concerns	Major concerns	No concerns	Major concerns	Low
XT+CM: YXTC+CM	Some concerns	Low risk	No concerns	Major concerns	No concerns	Major concerns	Low
YC+CM:YT+CM	Major concerns	Low risk	No concerns	Major concerns	No concerns	Major concerns	Very low
YC+CM: YXTC+CM	Some concerns	Low risk	No concerns	Major concerns	No concerns	Major concerns	Low
YT+CM: YXTC+CM	Some concerns	Low risk	No concerns	Major concerns	No concerns	Major concerns	Low

Abbreviations: CM, Conventional Medicine; TC, Tongxinluo Capsules; GT, Guanxinning Tablets; LHD, Lingbao Huxin Dan; XT, Xinkeshu Tablets; SBP, Shexiang Baoxin Pills; XC, Xinxuekang Capsules; QDP, Qishen Dripping Pills; DDP, Danshen Dripping Pills; STDP, Shexiang Tongxin Dripping Pills; YC, Yuxintong Capsules; YT, Yixinshu Tablets; XP, Xinbao Pills; YXTC, Yindan Xinnao Tong Capsules; SSP, Shenxiang Suhe Pills.

Appendix 11: Funnel plots

The funnel plot illustrates the assessment of small study effect bias in studies investigating the effects of various Commercial Chinese polyherbal preparations combined with conventional Western drugs versus conventional Western drug monotherapy on coronary microvascular disease. The figure encompasses all included trials that compared at least one combination therapy with a conventional Western drug alone as the control.

Figure S11.1: Funnel plot of IMR

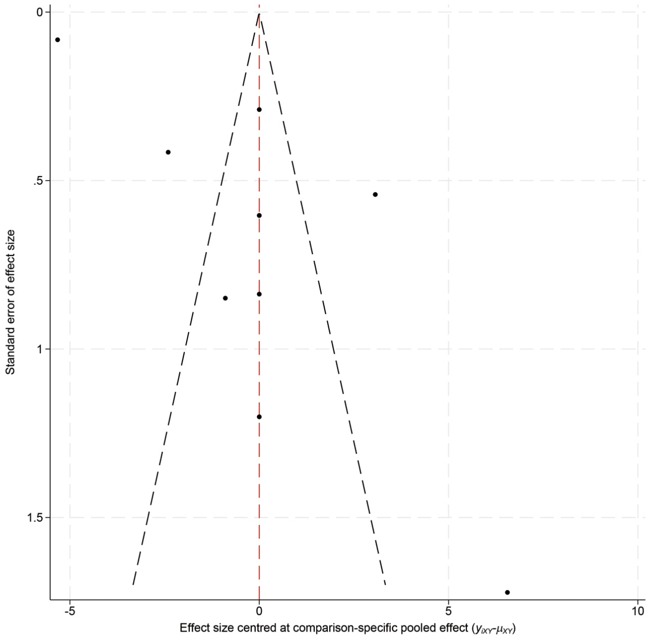

Figure S11.2: Funnel plot of CFR

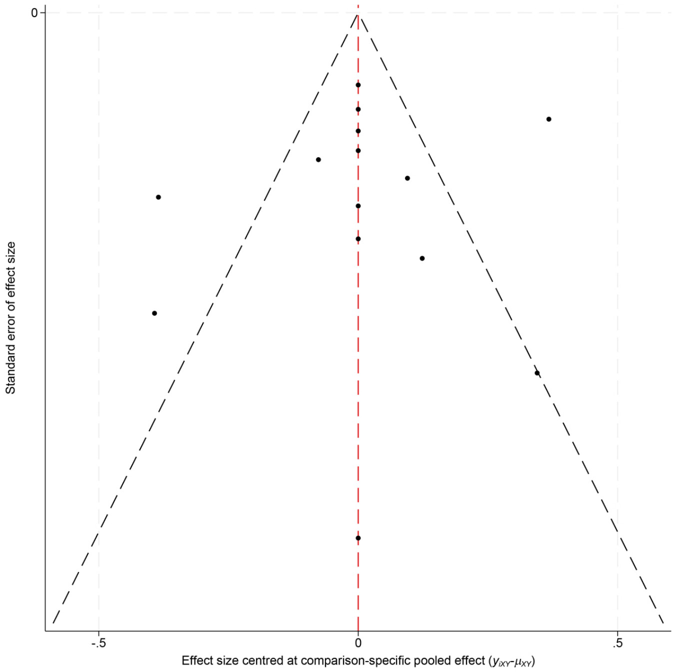

Figure S11.3: Funnel plot of cTFC

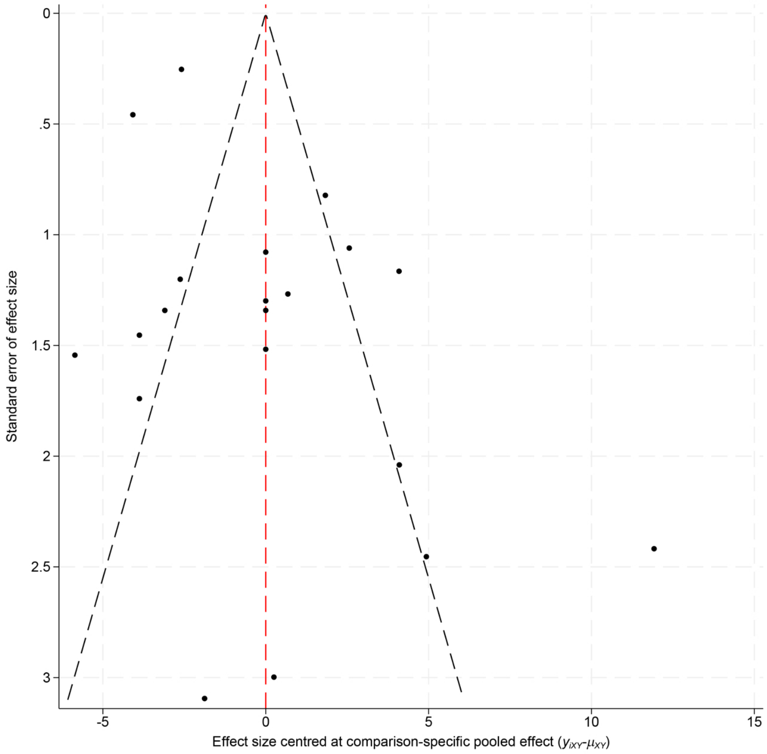

Figure S11.4: Funnel plot of the total effective rate

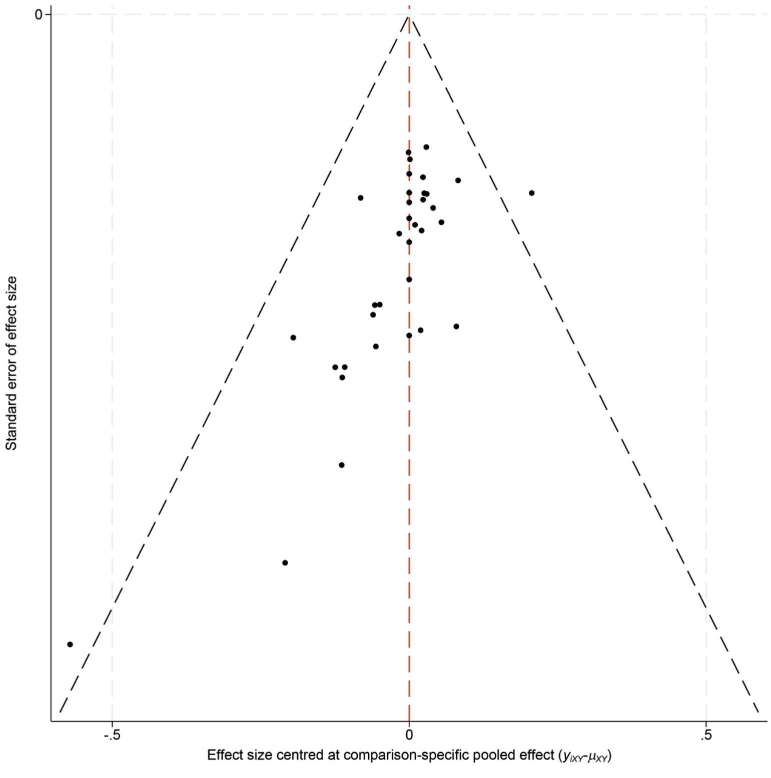

Figure S11.5: Funnel plot of LVEF

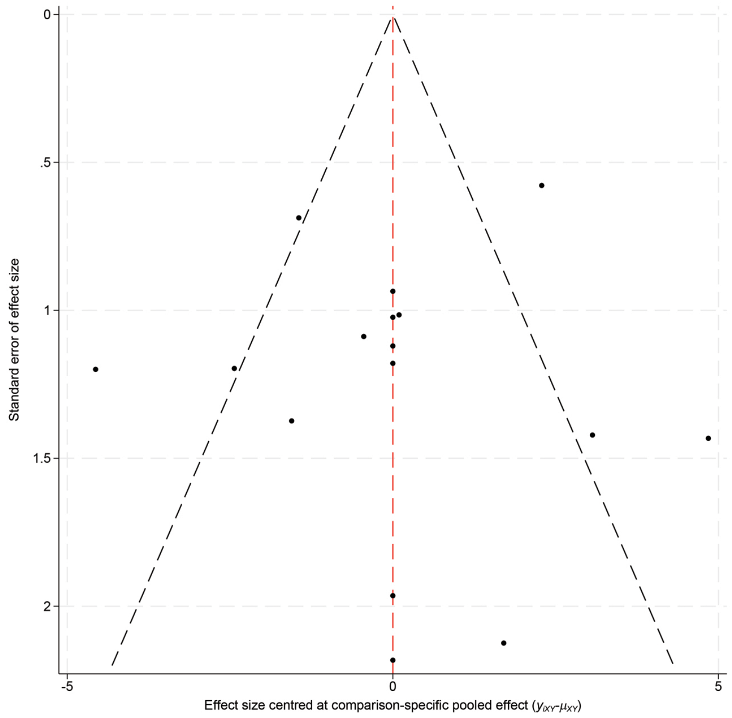

Figure S11.6: Funnel plot of hs-CRP

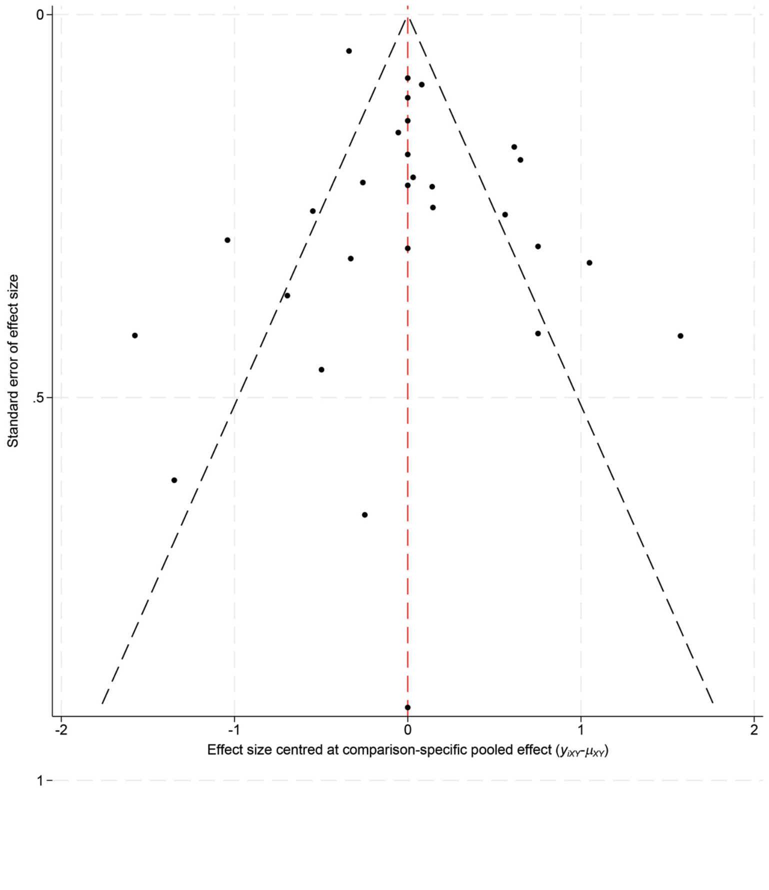

Figure S11.7: Funnel plot of ET-1

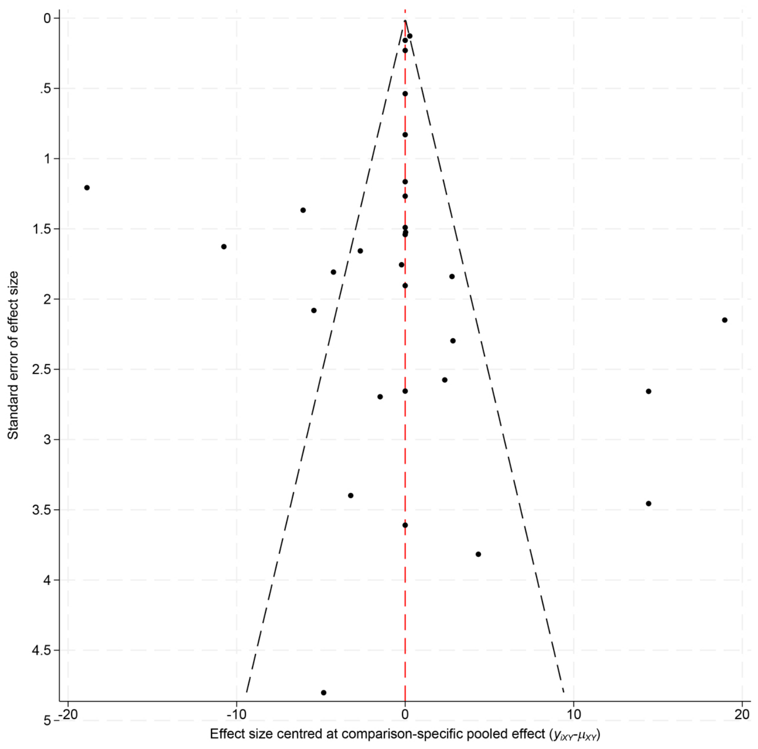

Figure S11.8: Funnel plot of NO

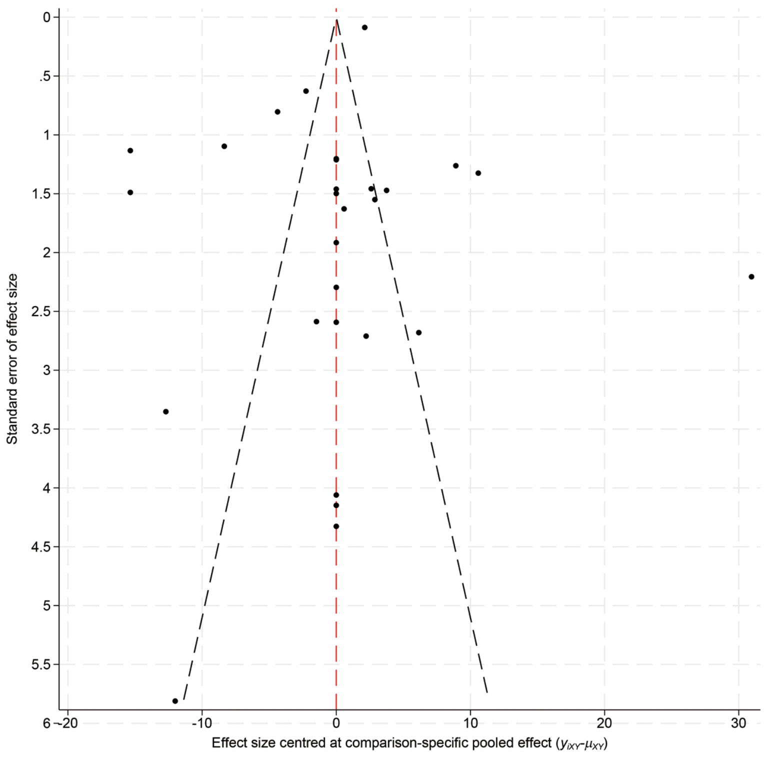

Figure S11.9: Funnel plot of Adverse events

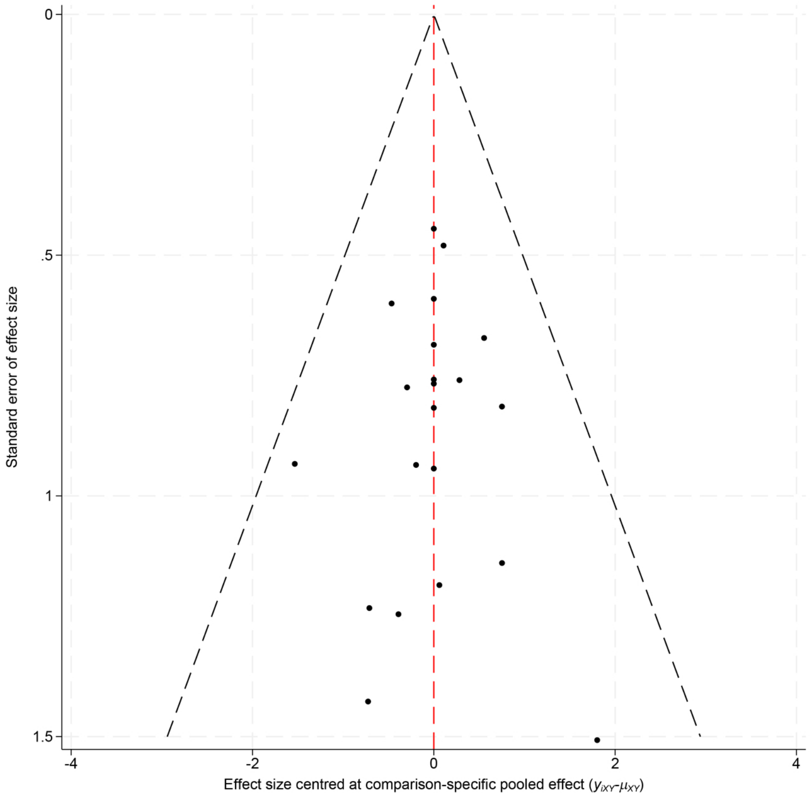

Appendix 12: Table S12 Sensitivity analyses of primary outcomes

Treatment	IMR		CFR		cTFC	
	Main estimate	Sensitivity analyses	Main estimate	Sensitivity analyses	Main estimate	Sensitivity analyses
YT+CM	-8.93 (-14.43, -3.42)	-8.93 (-16.38, -1.47)	1.05 (0.31,1.79)	1.05 (0.31,1.79)	NA	NA
STDP+CM	-3.50 (-11.55,4.55)	-3.50 (-11.23,4.23)	0.56 (0.04,1.09)	0.56 (0.04,1.09)	-8.75 (-14.37, -3.12)	-9.42 (-16.87, -1.98)
XT+CM	-3.38 (-11.10,4.34)	-3.38 (-10.77,4.00)	0.56 (-0.16,1.28)	0.56 (-0.16,1.28)	NA	NA
YXTC+CM	-5.10 (-12.64,2.44)	-5.10 (-12.64,2.44)	NA	NA	NA	NA
SBP+CM	-4.74 (-8.27, -1.20)	-6.13 (-9.94, -2.32)	0.50 (0.07,0.93)	0.50 (0.07,0.93)	-6.33 (-10.79, -1.86)	-7.62 (-13.97, -1.28)
DDP+CM	NA	NA	0.28 (-0.43,0.99)	0.28 (-0.43,0.99)	-8.50 (-17.75,0.74)	-8.57 (-18.98,1.84)
LHD+CM	NA	NA	0.36 (-0.36,1.08)	0.36 (-0.36,1.08)		
QDP+CM	NA	NA	0.62 (-0.25,1.49)	NA	-7.63 (-17.14,1.88)	-7.63 (-18.36,3.10)
TC+CM	NA	NA	0.81 (0.08,1.54)	0.81 (0.08,1.54)	-9.94 (-13.87, -6.01)	-8.04 (-13.35, -2.73)
XC+CM	NA	NA	0.32 (-0.39,1.03)	0.32 (-0.39,1.03)	NA	NA
XP+CM	NA	NA	0.81 (0.29,1.33)	0.81 (0.29,1.33)	NA	NA
GT+CM	NA	NA	NA	NA	-2.50 (-12.11,7.11)	-2.51 (-13.33,8.31)
KA+CM	NA	NA	NA	NA	-4.90 (-11.54,1.75)	-4.89 (-12.42,2.63)
QQC+CM	NA	NA	NA	NA	-6.10 (-15.59,3.38)	-6.10 (-16.81,4.61)

Appendix 13: Table S13. Safety and adverse events summary

Study ID (Year)	Intervention	Intervention group	Specific manifestations	Control group	Specific manifestations
Li Mengjie (2024)	SBP+CM	3/55	NA	2/55	NA
Yang Yangyang (2023)	SBP+CM	0/20	NA	6/20	NA
Wu Caiyun (2019)	SBP+CM	2/38	Rash (n=1), palpitations (n=1)	10/38	Headache (n=2), rash (n=2), poor appetite (n=2), palpitations (n=4)
Feng Haoli (2019)	SBP+CM	1/30	Recurrent angina (n=1)	3/30	Acute myocardial infarction (n=2) Recurrent angina (n=3)
Wang Shixun (2015)	SBP+CM	1/40	Headache (n=1)	2/40	Headache (n=2)
Zheng Wenhui (2024)	STDP+CM	2/52	NA	3/48	NA
Qin Xiaofei (2023)	STDP+CM	1/55	NA	1/56	NA
Han Yong (2024)	STDP+CM	1/44	Subcutaneous ecchymosis (n=1)	4/44	rash (n=1), abdominal pain (n=2), gingival bleeding (n=1)
Lai Haiqing (2025)	XT+CM	6/42	Hypotension (n=2), dizziness (n=2), nausea (n=2)	4/42	Hypotension (n=1), dizziness (n=1), nausea (n=2)
Zhao Danhua (2024)	XP+CM	2/100	NA	1/100	NA
Zhao Danhua (2021)	XP+CM	10/61	NA	11/61	NA
Wang Caige (2022)	YXTC+CM	3/65	NA	12/65	NA

Peng Cha'an (2024)	YXTC+CM	5/63	NA	8/63	NA
Kang Lirui (2021)	QDP+CM	4/30	NA	4/30	NA
Shen Xia (2021)	QDP+CM	5/34	NA	3/34	NA
Lai Peiwen (2024)	KA+SM	5/52	NA	3/52	NA
Liu Rui (2025)	XC+CM	3/41	NA	5/40	NA
Li Na (2025)	DDP+CM	2/52	NA	8/52	NA
Kong Xiao (2025)	YSG+CM	13/43	NA	18/49	NA
Wang Haiyang (2024)	LHD+CM	3/38	NA	2/38	NA
Li Chao (2025)	YT+CM	8/42	Nausea and vomiting (n=2), diarrhea (n=2), dizziness (n=2), headache (n=2)	6/42	Nausea and vomiting (n=1), diarrhea (n=3), hypotension (n=2)

Note: Intervention denotes the evaluated combination regimen (CCPP + conventional medicine, CM). Intervention group/Control group columns present the number of participants with adverse events divided by the total number of participants in each group. Specific manifestations list reported adverse event types and their corresponding counts (n). NA indicates that no specific manifestations of adverse events were described in the trial.

Abbreviations: CM, Conventional Medicine; YXTC, Yindan Xinnao Tong Capsules; DDP, Danshen Dripping Pills; SBP, Shexiang Baoxin Pills; STDP, Shexiang Tongxin Dripping Pills; XC, Xinxuekang Capsules; YSG, Yangxin Shengmai Granules; XP, Xinbao Pills; QDP, Qishen Dripping Pills; LHD, Lingbao Huxin Dan; YT, Yixinshu Tablets; XT, Xinkeshu Tablets; KA, Kuanxiong Aerosol.

Appendix 14: Table S14. Summary of top three interventions ranked by SUCRA for each clinical outcome

Outcome	Ranking	Intervention Regimen	SUCRA (%)	PrBest (%)	Mean Rank	Evidence Remark
IMR	1	YT+CM	88.4	61.8	1.6	Low certainty
IMR	2	YXTC+CM	57.3	17.3	3.1	Low certainty
IMR	3	SBP+CM	56.3	4.0	3.2	Insufficient evidence (wide confidence interval)
CFR	1	YT+CM	85.0	44.4	2.5	Low certainty
CFR	2	XP+CM	72.7	11.8	3.7	Low certainty
CFR	3	TC+CM	70.2	18.0	4.0	Low certainty
cTFC	1	TC+CM	79.5	22.2	2.6	Low certainty
cTFC	2	STDP+CM	69.5	15.3	3.4	Insufficient evidence (small sample size)
cTFC	3	DDP+CM	65.4	24.3	3.8	Insufficient evidence (limited studies)
Total effective rate	1	TC+CM	92.2	44.5	2.1	Moderate certainty
Total effective rate	2	GT+CM	79.8	34.6	3.8	Moderate certainty

Outcome	Ranking	Intervention Regimen	SUCRA (%)	PrBest (%)	Mean Rank	Evidence Remark
Total effective rate	3	LHD+CM	64.7	10.2	5.9	Moderate certainty
LVEF	1	STDP+CM	94.9	73.9	1.4	Insufficient evidence (limited studies)
LVEF	2	SSP+CM	65.0	7.7	3.8	Insufficient evidence (wide 95%CI)
LVEF	3	XC+CM	62.6	9.4	4.0	Low certainty
hs-CRP	1	YXTC+CM	93.4	54.6	1.9	Low certainty
hs-CRP	2	SBP+CM	84.5	9.2	3.0	Low certainty
hs-CRP	3	STDP+CM	83.0	14.8	3.2	Low certainty
ET-1	1	DDP+CM	91.4	36.7	2.4	Insufficient evidence (limited studies)
ET-1	2	STDP+CM	88.5	32.6	2.8	Low certainty
ET-1	3	XC+CM	85.1	23.9	3.4	Low certainty
NO	1	DDP+CM	88.5	42.8	2.7	Low certainty
NO	2	QDP+CM	73.4	9.2	5.0	Insufficient evidence (small sample size)

Outcome	Ranking	Intervention Regimen	SUCRA (%)	PrBest (%)	Mean Rank	Evidence Remark
NO	3	YXTC+CM	66.3	5.7	6.1	Insufficient evidence (limited data)
Adverse events	1	DDP+CM	88.0	50.1	2.4	Low certainty
Adverse events	2	SBP+CM	82.6	17.3	3.1	Insufficient evidence (limited safety data)
Adverse events	3	YXTC+CM	79.8	10.9	3.4	Low certainty
